# Preparation and Use of a General Solid-Phase Intermediate to Biomimetic Scaffolds and Peptide Condensations

**DOI:** 10.3390/molecules23071762

**Published:** 2018-07-18

**Authors:** J. Geno Samaritoni, Jacek G. Martynow, Martin J. O’Donnell, William L. Scott

**Affiliations:** 1 Department of Chemistry and Chemical Biology, Indiana University Purdue University Indianapolis, 402 N. Blackford St., IndiaFnapolis, IN 46202, USA; modonnel@iupui.edu (M.J.O.); wscott@iupui.edu (W.L.S.); 2 Melinta Therapeutics, Inc., 300 George Street, S 301, New Haven, CT 06511, USA; jmartynow@melinta.com

**Keywords:** distributed drug discovery, peptide fragment condensation, biomimetic scaffolds, bicyclic thiazolidine lactams, cyclitive cleavage, homoserine lactones, diastereomers, acetal, orthogonal protection, nuclear Overhauser enhancement

## Abstract

The Distributed Drug Discovery (D3) program develops simple, powerful, and reproducible procedures to enable the distributed synthesis of large numbers of potential drugs for neglected diseases. The synthetic protocols are solid-phase based and inspired by published work. One promising article reported that many biomimetic molecules based on diverse scaffolds with three or more sites of variable substitution can be synthesized in one or two steps from a common key aldehyde intermediate. This intermediate was prepared by the ozonolysis of a precursor functionalized at two variable sites, restricting their presence in the subsequently formed scaffolds to ozone compatible functional groups. To broaden the scope of the groups available at one of these variable sites, we developed a synthetic route to an alternative, orthogonally protected key intermediate that allows the incorporation of ozone sensitive groups after the ozonolysis step. The utility of this orthogonally protected intermediate is demonstrated in the synthesis of several representative biomimetic scaffolds containing ozonolytically labile functional groups. It is compatible with traditional Fmoc peptide chemistry, permitting it to incorporate peptide fragments for use in fragment condensations with peptides containing cysteine at the N-terminus. Overall yields for its synthesis and utilization (as many as 13 steps) indicate good conversions at each step.

## 1. Introduction

**Scheme 1:**
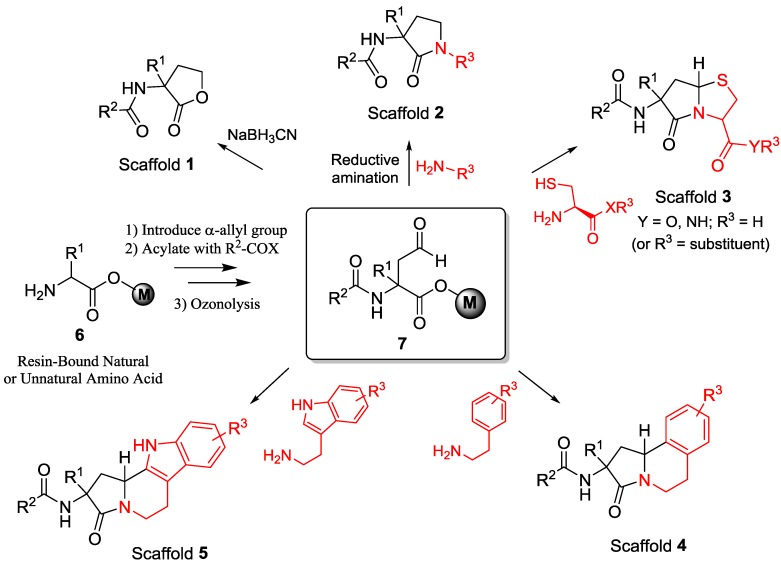
Synthesis and utilization of key intermediate **7** to multiple biomimetic scaffolds.

Our Distributed Drug Discovery (D3) program seeks simple and powerful synthetic methodologies to make large numbers of biomimetic molecules from diverse scaffolds. Solid-phase synthetic protocols are ideal, because they enable multi-step syntheses to be carried out efficiently, on a small or large scale, with quick, simple work-ups and a minimal loss of material. They are the most powerful when a single protocol allows access through more than one variable step to multiple molecules with potential for biological activity. The first dramatic example was Merrifield’s solid-phase synthesis of peptides [1,2], where each variable position in the growing peptide chain provided an opportunity to substitute any of 20 amino acids, and the ultimate peptide could be many amino acids long. We developed a variation on solid-phase peptide synthesis termed “unnatural peptide synthesis” [3,4,5], in which at a particular step of a small peptide synthesis, an N-terminal glycine could be converted on-resin into an unnatural amino acid that then became part of the growing sequence. Later, we extended this work with the solid-phase conversion of natural amino acids (including glycine) into the key Merrifield resin intermediate **7** [6], which was subsequently transformed into multiple peptidomimetic and biomimetic scaffolds **1**–**5** (Scheme 1). The final release of product in all of these cases was by cyclitive cleavage. This permits the use of acidic or basic conditions at any intermediate step [6].

**Scheme 2:**
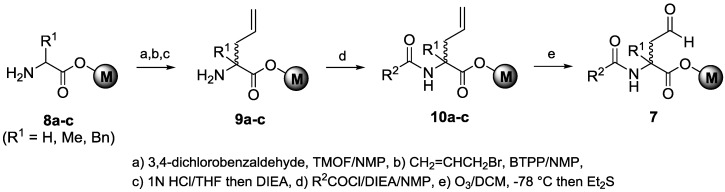
Previous synthesis of **7** (route is incompatible with unstable R^2^ during ozonolysis).

**Scheme 3:**
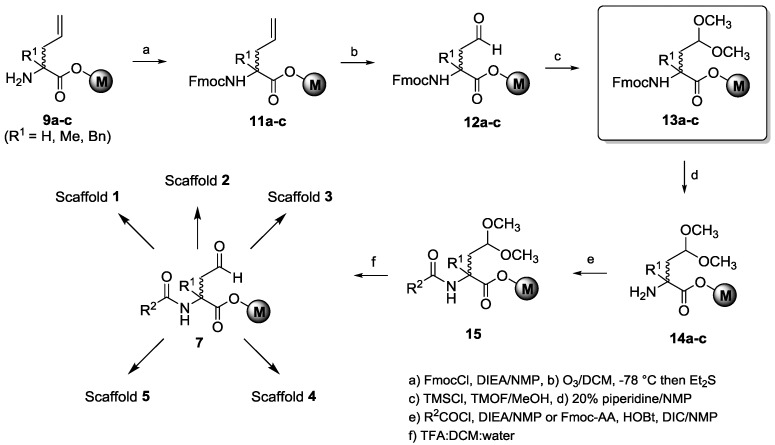
Preparation of alternative key intermediate **13** compatible with many R^2^.

To make these scaffolds, our previously reported [6] synthesis of **7** (Scheme 2) required an ozonolysis of precursor **10** that already contained the acylated residue R^2^. A major limitation of this route is its incompatibility with any R^2^ groups that are ozonolytically labile, such as electron-rich (hetero)aromatics, alkenes, and a number of amino acid side chains. In this report, we describe the synthesis and utilization of the modified key intermediate **13** (Scheme 3), which is compatible with both the introduction of ozone sensitive R^2^ (for example, the side chains on some amino acids such as tyrosine or tryptophan) and classic solid-phase peptide synthesis. In addition to giving broader access to the biomimetic scaffolds shown in Scheme 1, it also permits peptide fragments to be coupled with N-terminal cysteine peptides, resulting in thiazolidine lactam scaffolds **3**, in which either or both R^2^ and R^3^ are amino acids or peptide derived. The latter case represents an example of peptide fragment condensation.

The strategy used to overcome the limitation represented by Scheme 2 was to perform the ozonolysis on the N-protected fluorenylmethyloxycarbonyl (Fmoc) derivative **11** (Scheme 3) prior to introduction of the ozone-sensitive acyl group R^2^CO. The newly formed aldehyde **12** could then be orthogonally protected as the key acetal intermediate **13** [7]. Now, the latent amino and aldehyde groups present in **13** can be selectively unmasked and functionalized, providing access to **7** with ozonolytically labile compounds, and by a process compatible with Fmoc-based solid-phase peptide synthesis (SPPS), a route to diverse unnatural peptides and peptidomimetics **1**–**5** (Scheme 1), as well as peptide fragment condensations. The potential for this methodology is illustrated in this report for representative scaffolds **1** and **3**.

Scaffold **1** compounds, N-acyl homoserine lactones (AHLs), have received considerable attention over the last 10–15 years. Certain natural Scaffold **1** compounds, such as N-acyl homoserine lactones, are known to be used by Gram-negative bacteria as signaling molecules to initiate quorum sensing [8,9]. In early efforts designed to mimic endogenous signaling lactones, non-native AHLs have been identified as inhibitors of quorum sensing [9,10], and this has stimulated a growing search for inhibitors of biofilm formation in recent years [11,12,13].

Scaffold **3** compounds, γ-bicyclic thiazolidine lactams, have been of particular interest and can be traced back to the years of the Second World War when the various structure–activity relationships associated with the penicillins were being defined [14]. Subsequently, it was recognized that fused bicyclic structures such as **3** can mimic conformationally-restricted peptides [15,16,17,18,19]. Often, they adopt a β-turn conformation [20,21,22]: a secondary, reverse-turn structure that has been associated with various biological activities [17,18,19,23,24]. The replacement of dipeptide residues with a conformationally-restricting bicyclic thiazolidine lactam core has found application in its incorporation into the antimicrobial gramicidin S [25] and the hypertensive angiotensin II [26].

## 2. Results and Discussion

### 2.1. Preparation of Fmoc Acetal Intermediate Resins ***13***

Resin **9c** (R^1^ = Bn) [6] was converted to its Fmoc derivative **11c** using Fmoc-Cl according to Scheme 3. Although amine **9c** represents a potentially problematic or difficult coupling [27,28,29,30] by virtue of its quaternary α-carbon, there was no evidence in the subsequently released products of incomplete acylation with the reactive Fmoc-Cl. The Fmoc functional group did not present any complications during ozonolysis of **11c** followed by reductive work-up [31]. The resultant aldehyde functional group in **12c** was then converted to its dimethylacetal **13c** using methanol, trimethylsilyl chloride, and trimethylorthoformate [7,32]. Similarly, **9a**,**b** were converted to **13a**,**b**.

### 2.2. Diverse Applications of Fmoc Acetal Intermediate Resins ***13***

#### 2.2.1. Demonstrating Compatibility with Ozonolytically Labile R^2^

As a demonstration of the usefulness of resins **13a**–**c**, examples of scaffolds **1** and **3** bearing ozone-labile substructures are presented.

##### Scaffold **1**

**Scheme 4:**
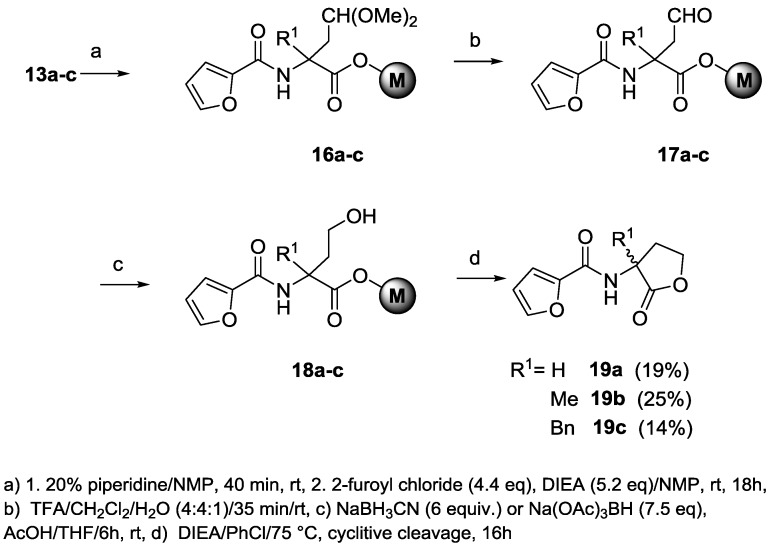
Introduction of the ozone-labile furan ring.

The approach to the preparation of homoserine lactone scaffolds **1** is illustrated with the furanyl derivatives **19a**–**c** (Scheme 4). Treatment of **13a**–**c** with 20% piperidine followed by acylation of the deprotected amine resin with 2-furoyl chloride gave the acylated acetal resins **16a**–**c**. The latent aldehyde was regenerated by a brief exposure (35 min) to aqueous trifluoroacetic acid, providing resins **17a**–**c**. Reduction to the alcohol resins **18a**–**c** was accomplished with sodium triacetoxyborohydride in acetic acid, and this was followed by cyclitive cleavage at elevated temperatures in the presence of diisopropylethylamine (DIEA, Hünig’s base) to give the desired lactones **19a**–**c** bearing the ozone-labile furan ring [33,34] in 14–25% overall yield (80–86% average yield per step over 11 steps from starting substituted Merrifield resins).

##### Scaffold **3**

**Scheme 5:**
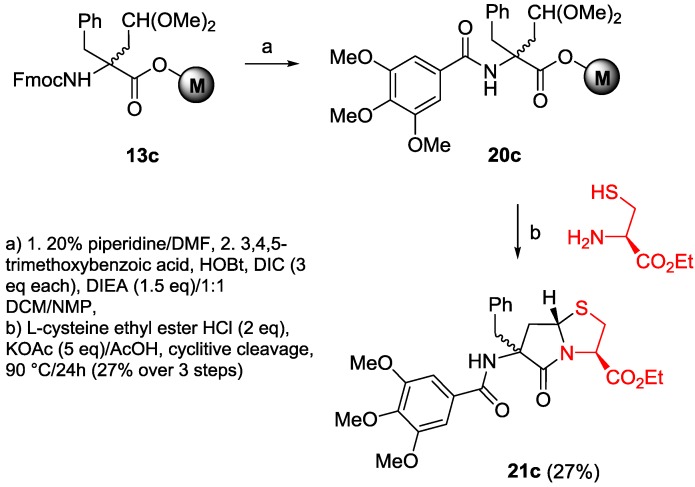
Introduction of an electron-rich aromatic ring.

The synthesis of a scaffold **3** example featuring an ozone-sensitive group is illustrated in Scheme 5. Fmoc acetal resin **13c** was deprotected and acylated with the electron-rich, ozone-sensitive, activated ester of the trimethoxybenzoic acid generated with 1-hydroxybenzotriazole (HOBt) and diisopropylcarbodiimide (DIC) to give acetal resin **20c**. Cyclitive cleavage (step b) at room temperature failed, resulting in the recovery of starting material, but did proceed at elevated temperature to give a 27% overall purified yield of **21c** as a 2:1 mixture of diastereomers. The required thiazolidine intermediate was formed through in situ acetic acid activation of the dimethylacetal and reaction with cysteine ethyl ester.

#### 2.2.2. Demonstrating Compatibility with Peptide Chemistry

As a demonstration of the usefulness of resins **13a**–**c** as advanced intermediates in solid-phase peptide synthesis and fragment condensation, examples of scaffolds **1** and **3** bearing amino acid residues are presented.

##### Scaffold **1**

**Scheme 6:**
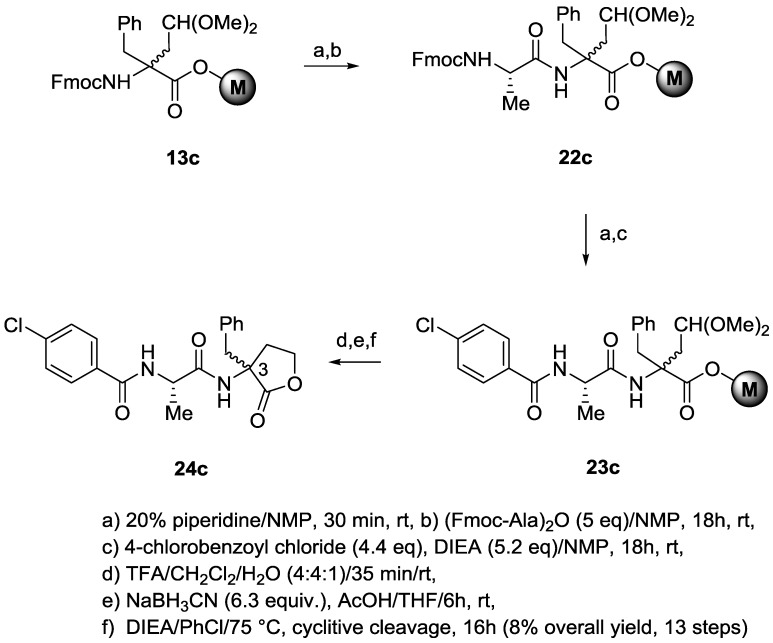
Introduction of amino acid residue into Scaffold **1**.

Using standard Fmoc-based peptide synthetic procedures, advanced resins **13a**–**c** also provide the opportunity to install amino acid or peptide residues into scaffold **1** structures. Scheme 6 depicts the successful incorporation of an N-capped amino acid residue at the N-terminus of the homoserine lactone scaffold **1**. After deprotection of **13c**, the amine was acylated using the anhydride of Fmoc-Ala-OH. Subsequent deprotection and acylation with 4-chlorobenzoyl chloride gave the *N*-capped, *N*-terminal alanine acetal resin **23c**. Acetal hydrolysis, cyanoborohydride reduction, and cyclitive cleavage then gave, in nearly equal quantities, the stereoisomers of **24c** (54:46 ratio), which were separated by reverse-phase chromatography to give the *3R* and *3S* diastereomers.

##### Scaffold **3**

**Scheme 7:**
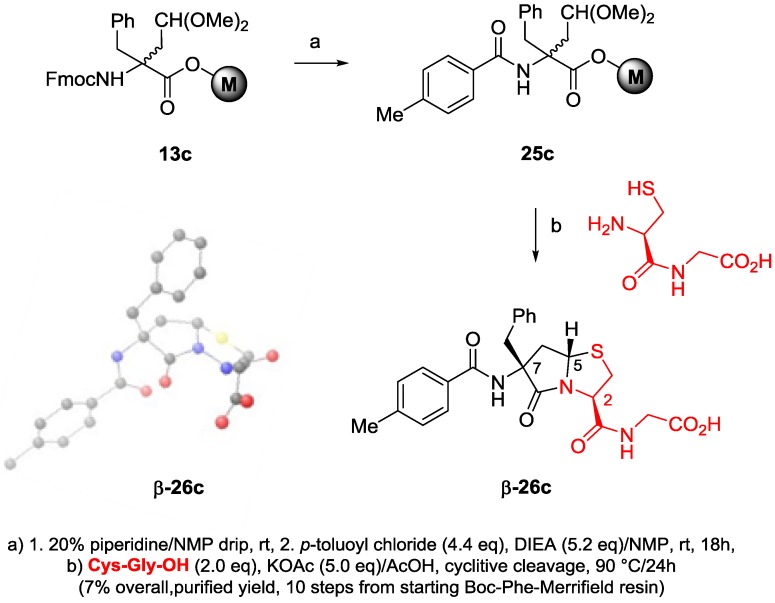
Introduction of glycine residue into Scaffold **3**.

**Figure 1:**
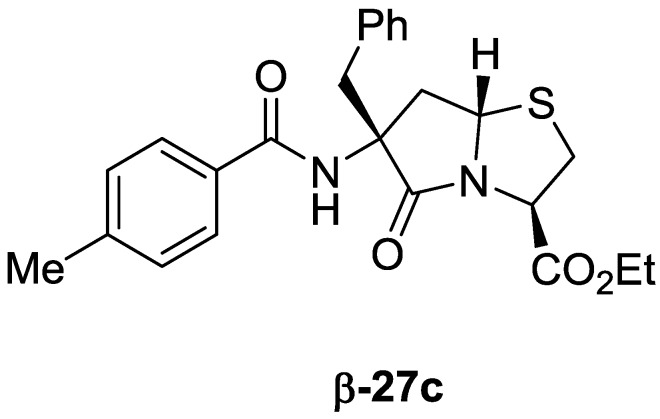
Previously established stereochemistry of **β**-**27c**.

Again, using standard Fmoc-based peptide synthetic procedures, amino acid or peptide residues can be incorporated into advanced resins **13a**–**c** at (1) the N-terminus of the bicyclic framework of **3**, and at (2) the C-terminus of the bicyclic framework of **3** during the bicyclization using N-terminal cysteine peptides. This second opportunity was first fulfilled with the incorporation of a glycine residue at the C-terminus of scaffold **3** (Scheme 7). Following deprotection of **13c**, the amine resin was acylated with *p*-toluoyl chloride. Cyclitive cleavage using commercially available Cys-Gly-OH at elevated temperature then afforded a 3:1 mixture of diastereomers, which when triturated with dichloromethane and recrystallized from ethanol resulted in the isolation of a single diastereomer, as evidenced by proton NMR spectroscopy. The structure of this stereoisomer, which was solved by x-ray crystallography (Table 1), was found to be the *2R,5S,7S* isomer **β**-**26c**; this bears the identical configuration pattern as the previously reported **β**-**27c** (Figure 1) [6]. Compound **β**-**26c** represents our first example of a fragment condensation [35,36,37,38] in which the C-terminal fragment is Cys-Gly, and the N-terminal fragment is an N-acylated modified, resin-bound phenylalanine.

As demonstrated with the two examples in Scheme 5 and Scheme 7, cyclitive cleavage of dimethylacetal resins can be executed without initial conversion to the aldehyde resins. However, elevated temperatures (90 °C) were required. We later sought milder cyclitive cleavage conditions in an effort to improve upon the diastereoselectivity. Consequently, all of the subsequent examples reported below illustrate the cyclitive cleavage to Scaffold **3** compounds occurring from the aldehyde resins.

**Scheme 8:**
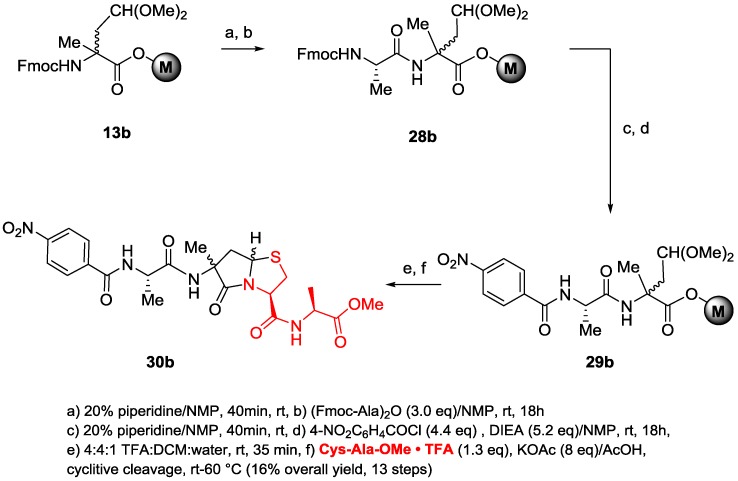
Fragment condensation of an Fmoc acetal resin with Cys-Ala-OMe.

**Scheme 9:**
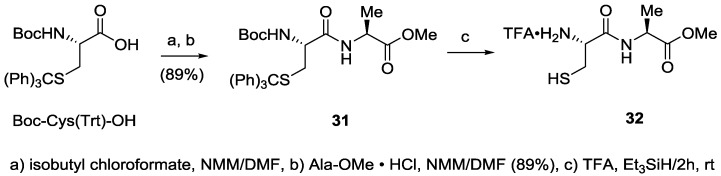
Preparation of stereochemically pure Cys-Ala-OMe.

Compound **30b** (Scheme 8) serves as an example of a scaffold **3** structure bearing alanine residues at both the R^2^ and R^3^ positions, and represents another fragment coupling illustration (dipeptide + dipeptide). The required Cys-Ala-OMe **32** was prepared using the mixed anhydride method (Scheme 9). Originally **31**, which is the precursor to **32**, was prepared by a solid-phase synthetic route in which cleavage from Wang resin was carried out in methanol/triethylamine at 55–60 °C for 42 h. However, concern for epimerization resulting from prolonged exposure to mild base at elevated temperature was substantiated as **31**, which when prepared by this method was found to be a 95/5 mixture of diastereomers. When **31** was prepared by the mixed anhydride method (Scheme 9), it was stereochemically pure.

#### 2.2.3. Variation of R^1^, R^2^, and YR^3^ of Scaffold **3** Structures

**Scheme 10:**
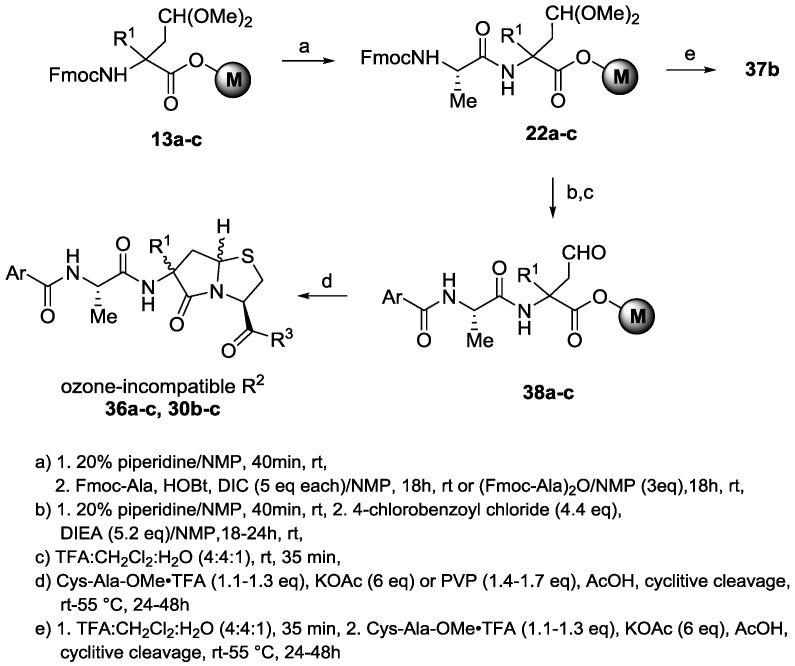
Synthesis of Scaffold **3** compounds with ozone-incompatible groups and amino acid residues.

**Scheme 11:**
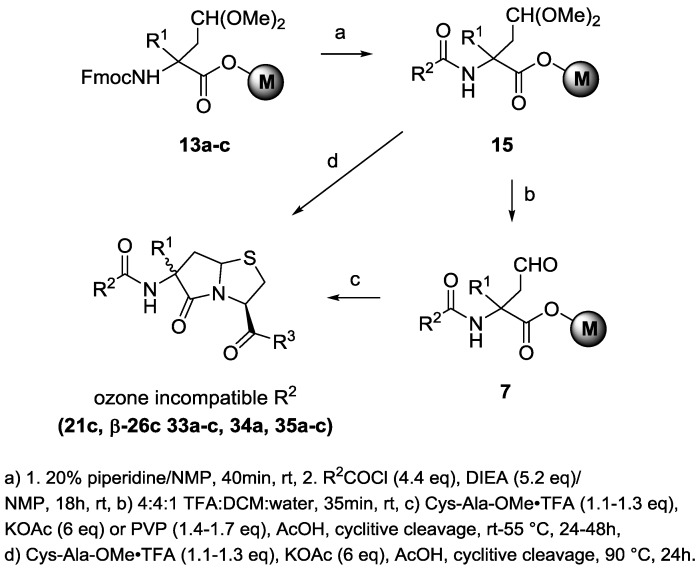
Synthesis of Scaffold **3** compounds with ozone-incompatible groups and R^2^ ≠ amino acid residues.

**Scheme 12:**
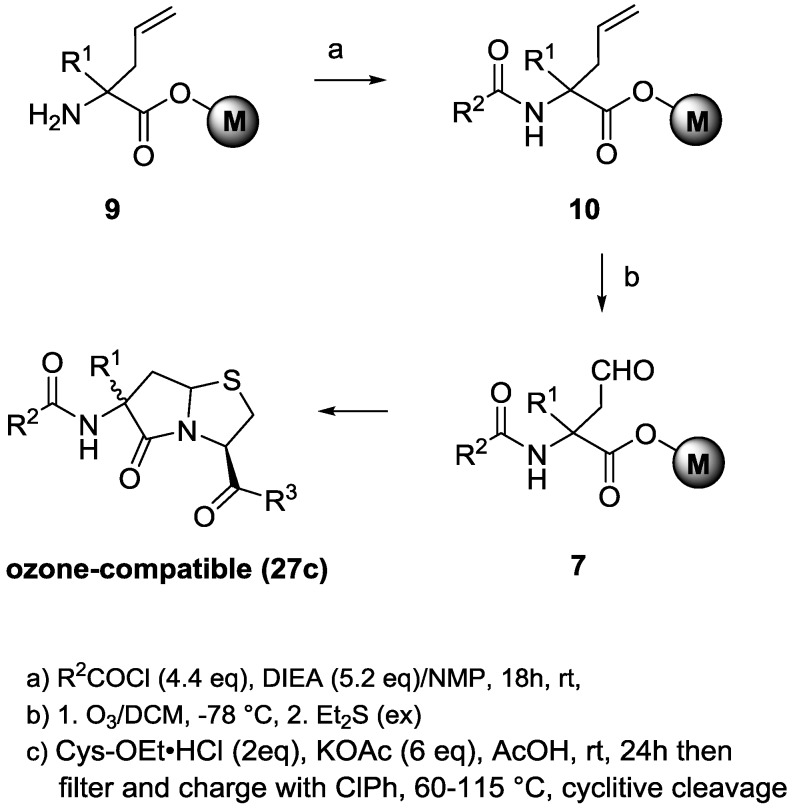
Synthesis of Scaffold **3** compounds with ozone-compatible R^2^.

Variations of the three substituents R^1^, R^2^, and YR^3^ of scaffold **3** are defined in Table 2 and include R^1^ = H, Me, and Bn; R^2^ = aryl and aroyl-Ala; YR^3^ = NH-Gly-OH, NH-Leu-OMe, NH-Ala-OMe, and OEt. The synthetic routes used to prepare these compounds are described in Scheme 10, Scheme 11 and Scheme 12. Bicyclic thiazolidine lactams **3** with ozone-incompatible groups are represented in Scheme 10 (amino acid residues present) and Scheme 11 (acyl groups R^2^CO present), whereas **3**, with ozone-compatible R^2^CO, was prepared using Scheme 12. As Scheme 11 indicates, the bicyclic thiazolidine lactam targets can be accessed in a single step (**step d**) from acetal resin **15** (vide supra). If milder conditions are required, the two-step process (steps b and c) allows cyclitive cleavage to proceed at slightly lower temperatures. Table 2 compounds are listed below each target structure in the three schemes.

**Scheme 13:**
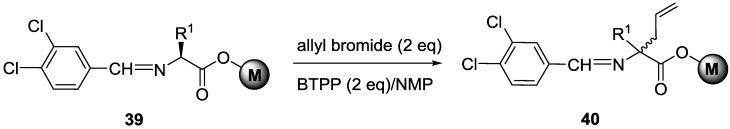
Origin of racemic stereochemistry at C-terminal α-carbon of **13**.

Preparation of all the above scaffold **3** compounds and the others presented in Table 2 from Fmoc acetal resins **13a**–**c** resulted in the formation of two predominating diastereomers in each case, and their diastereomeric ratios (dr) are given. The isolation of two major stereoisomers is a consequence of the achiral alkylation of **39**; this introduces the allyl side chain, which is the precursor to the acetal moiety of **13a**–**c** (Scheme 13). Although two additional diastereomers were observed in cases where R^1^ = H and Me, they accounted for less than 10% of the product mixture. These two additional diastereomers are not represented in Table 2, and no effort was made to isolate and characterize them. Standard reverse-phase chromatography was used to separate the diastereomers. The overall yields (1–29%) from the commercially available starting resins, based on advertised loadings, are also provided with the number of steps (5–13) recorded in parentheses. The overall yields represent an average of 76–87% per step.

**Scheme 14:**
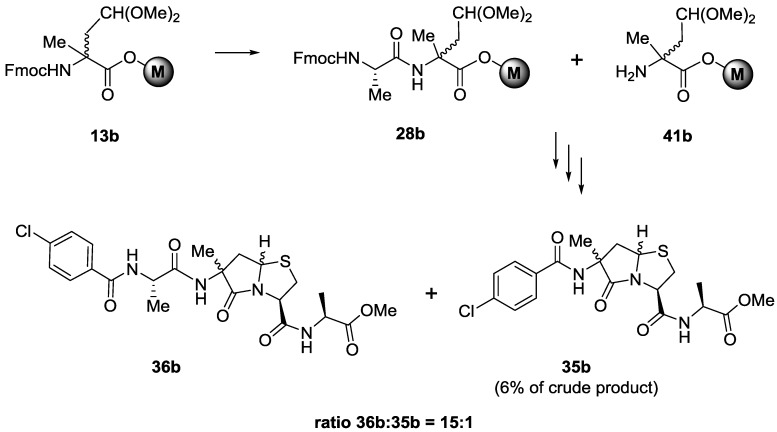
Problematic coupling leading to the deletion of peptide **35b**.

Similar to the case with the preparation of **22c** (Scheme 6), when the R^1^ of **13** is methyl, coupling with Fmoc-Ala-OH using hexafluorophosphate benzotriazole tetramethyl uronium (HBTU) was found to be incomplete, resulting in the isolation of a small amount of the deletion peptidomimetic **35b** along with the desired **36b** (Scheme 14). Problematic coupling was also observed with **13c** (R^1^ = Bn). These two examples (R^1^ = Me and Ph) represent difficult couplings [27,28,29,30] in peptide synthesis in which one or both fragments possess a dialkylated α-carbon adjacent to their amine and carboxyl reaction centers such as the aminoisobutyryl (Aib) moiety [28,29,30]. Such couplings, for example, have been carried out most efficiently using the highly-reactive Fmoc acid fluorides [39], urethane-protected amino acid *N*-carboxyanhydrides (UNCAs) [40], and symmetrical anhydrides [41,42,43,44] as the activated carboxyl contributor. Use of the coupling reagents hexafluorophosphate azabenzotriazole tetramethyl uronium (HATU) [29], the benzotriazole (1H-1,2,3-benzotriazol-1-yloxy)-tris(pyrrolidino)-phosphonium hexafluorophosphate (PyBOP) [30], and the phosphonium bromide bromo-tris(pyrrolidino)-phosphonium hexafluorophosphate (PyBroP) [30] have also led to efficient coupling involving the highly-hindered Aib. Four methods were explored in the search for a more efficient method to couple Fmoc-Ala-OH with **13b**,**c**: (a) Fmoc-Ala-OH/HBTU/diisopropylethylamine [41,42]; (b) Fmoc-Ala-OH/HOBt/DIC [41,43]; (c) the symmetrical anhydride [41,44] (Fmoc-Ala)_2_O and (d) the acid chloride [41] Fmoc-Ala-Cl [45]. Use of the symmetrical anhydride (method c) was found to be the superior method in this application.

### 2.3. Aspects of Bicyclic Thiazolidine Lactam NMR Spectra

#### 2.3.1. Use of the Nuclear Overhauser Enhancement to Assign Stereochemistry

**Figure 2:**
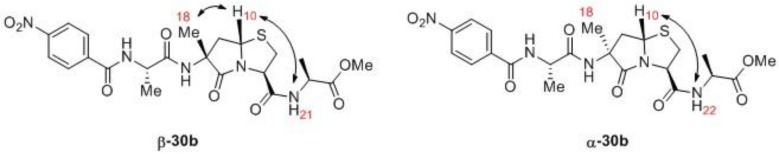
Selected Nuclear Overhauser enhancement (nOe) results from irradiation of diastereomeric **30b**.

It is assumed that epimerization at the alpha carbon originating with the cysteine reagent had not taken place under the mildly acidic conditions for cyclitive cleavage [46]. To determine the configurations at the other two stereocenters of **3**, both one-dimensional and two-dimensional (2D-NOESY) nuclear Overhauser enhancement (nOe) experiments were performed. The results from diastereomers **α**-**30b** and **β**-**30b** are illustrative. Irradiation of the methyl protons (H-18) of **β**-**30b** resulted in the enhancement of the bridgehead proton H-10, and the enhancement was reciprocated upon irradiation of H-10 (Figure 2). In addition, the irradiation of H-10 gave an enhancement at amide proton H-21. These enhancements were observed in both the one-dimensional and two-dimensional experiments, and are consistent with the methyl group H-18, the bridgehead proton H-10, and the amide proton H-21 oriented *syn* to one another. In contrast, no nOe to H-10 is observed upon irradiation of the methyl group of **α**-**30b**, nor in the reverse direction. Also, for **α**-**30b**, reciprocal enhancements are observed between protons H-10 and H-22 upon irradiation of either proton establishing their *syn* relationship to one another.

#### 2.3.2. Identification of Chemical Shift Differences to Assign Stereochemistry 

**Figure 3:**
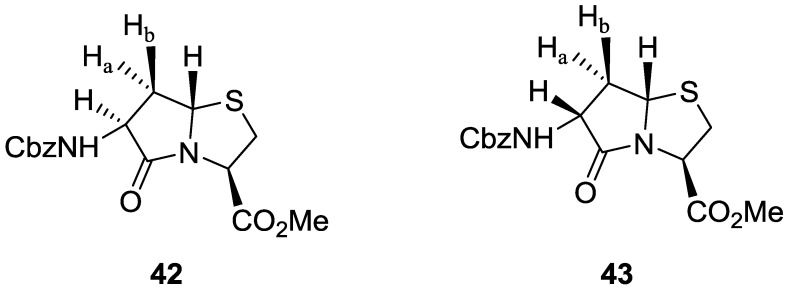
α and β diastereomers **42** and **43**.

Table 3 compiles the chemical shifts for the ring fusion proton and the two diastereotopic protons on the adjacent carbon atom (labeled as H_x_, H_y_, and H_z_, respectively) for the Scaffold **3** compounds described in Table 2, and illustrates a predictive aspect of stereoisomer assignment. The compounds are arranged in three groups according to their R^1^ substituent (H, Me, or Bn). Also provided as ∆δ_yz_ (ppm) is the difference in chemical shift between the two diastereotopic protons of each pair of diastereomers. Listed next to this column is the nOe-assigned stereochemistry of the R^1^ group, which is described as α (R^1^ *anti* to cysteine carbonyl group or down) or β (R^1^ *syn* to cysteine carbonyl group or up). Comparison of the R^1^ = benzyl series shows without exception that the ∆δ is significantly larger for the β isomer. The ∆δ is even larger with the β isomer for the R^1^ = H series; however, it is the α isomer in the R^1^ = Me series that displays the larger ∆δ values. Baldwin et al. [47] observed the same trend in the R^1^ = H series with diastereomers **42** and **43** (Figure 3), with the β isomer **43** showing the larger ∆δ value between the chemical shifts of H_a_ and H_b_.

#### 2.3.3. Use of the Nuclear Overhauser Enhancement to Assess Molecular Shape

**Figure 4:**
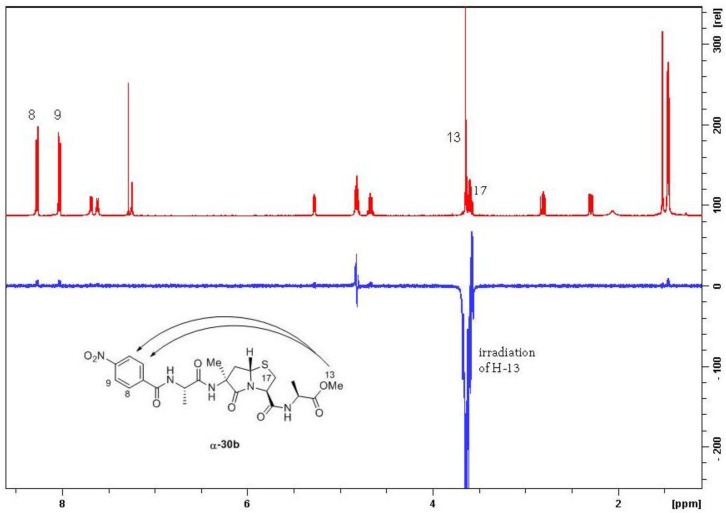
Remote nOe at H-8 and H-9 from irradiation at H-13 of **α-30b**.

The γ-bicyclic thiazolidine lactam core imparts conformational restriction [15,16,17,18,19] and when suitably substituted with amino acid residues, the resulting peptidomimetic can adopt a β-turn secondary structure [20,21,22]. Typically, this reverse-turn involves a tetrapeptide segment and is observed in optical rotatory dispersion (ORD) and circular dichroism (CD) spectra. From one-dimensional nOe analyses of the diastereomers of **30b**, a small enhancement (0.1%) was observed at the aromatic protons H-8 and H-9 upon irradiation of the methyl protons, H-13, of the α-isomer, **α**-**30b** (Figure 4). A reciprocated enhancement was observed at the ester methyl protons upon irradiation of H-8. No such enhancements were observed with **β**-**30b**. These results are consistent with an approach of the termini of **α**-**30b**, and may reflect the adoption of a β-turn secondary structure by this stereoisomer in chloroform solution.

## 3. Materials and Methods

All of the reagents were purchased from Acros Organics (Geel, Belgium), Advanced Chemtech (Louisville, KY, USA), EMD Millipore (Burlington, MA, USA), or Sigma-Aldrich Chemical (St. Louis, MO, USA), and were used without further purification. Boc-protected amino acids on Merrifield resin were purchased from Polymer Laboratories, Amherst, MA, USA (now Agilent Technologies, Santa Clara, California, USA). Solid-phase peptide synthesis (SPPS) vessels were purchased from Chemglass (Vineland, NJ, USA). ^1^H-NMR and ^13^C-NMR spectra were recorded on a Bruker Avance III 500 and 400 spectrometers (^1^H, 500.13 MHz; ^13^C, 125.76 MHz) (Billerica, MA, USA) using tetramethylsilane as internal standard in CDCl_3_ or unless otherwise indicated. Chemical shifts are reported in parts per million (ppm, δ units). Coupling constants, *J*, are reported in Hertz (Hz). Splitting patterns are designated as s: singlet, br s: broad singlet, d: doublet, dd: doublet of doublets, ddd: doublet of doublets of doublets, q: quartet, and m: multiplet. High-resolution mass spectra (HRMS) were obtained using a Thermo Electron Corporation MAT 95XP-Trap (Waltham, MA, USA) or an Agilent 1200 HPLC-6130 MSD Mass Spectrometer (Santa Clara, CA, USA) in the electrospray ionization (ESI) or in the chemical ionization (CI) modes at the Mass Spectrometry Facility of Indiana University, Bloomington, IN. High performance liquid chromatography-mass spectrometry (HPLC/MS) was performed on an Agilent 1100 Series LC/MSD (Santa Clara, CA, USA) in the electrospray ionization positive mode using a 4.6 × 150 mm Agilent Eclipse XDB five-micron C18 reverse-phase column. Nuclear Overhauser enhancement (nOe) 1-D difference spectroscopy was conducted using the Bruker pulse sequence SELNOGP (Billerica, MA, USA) with the following acquisition parameter changes: Spectral width, SW = 10.0 ppm, Transmitter frequency offset, O1P = 4.0 ppm, Duration delay, D[8] = 0.6 s.

**α-Allyl-α-R^1^-*N-*(fluorenylmethyloxycarbonyl)glycine on Merrifield resins (11a–c, R^1^ = H, Me, Bn).** The amine resin **9a**–**c** (R^1^ = H, Me, Bn, 7.60–8.50 mmol) [3], contained in a 500-mL SPPS vessel, was washed with 4 × 10 mL of *N*-methyl pyrrolidinone (NMP). To the resin was then added 40 mL of NMP, followed by 3.8 equivalents of diisopropylethylamine (DIEA). The vessel was swirled gently to mix the contents, and 3.0 equivalents of Fmoc chloride in 60–70 mL of NMP was added in one portion. The vessel was rocked on an orbital shaker and after 24 h the vessel was drained, and the resin was washed 3 × 45 mL each with NMP, 1:1 THF:EtOH, THF, and dichloromethane (DCM) to give resins **11a**–**c** (R^1^ = H, Me, Bn). The resin was then dried under a slow stream of dry nitrogen gas overnight.

**α-(2-Oxoethyl)-α-R^1^-*N-*(fluorenylmethyloxycarbonyl)glycine on Merrifield resins (12a–c, R^1^ = H, Me, Bn).** A 250-mL, three-neck, round-bottomed flask was charged with 1.80–3.20 mmol of resin **11a**–**c** (R^1^ = H, Me, Bn), a trace of Sudan III red dye, and 40 mL of DCM under dry argon gas. The contents were cooled in a dry ice/acetone bath, and the argon flow was replaced with a subsurface flow of oxygen gas at a rate of 0.6–0.8 L/min. Ozonolysis using an ozone generator was performed at a current of 1.0 ampere until the red dye was rendered colorless (2–3 h). The current was then reduced to zero while oxygen bubbling continued for 10 min. Diethyl sulfide (1.0 mL, 9.3 mmol) was added, and the solution was allowed to gradually warm to room temperature and stir overnight. The resin was collected by filtration into a 50-mL SPPS vessel rinsing over with DCM and THF. The resin was dried under a slow stream of dry argon gas and then under high vacuum to give **12a**–**c** (R^1^ = H, Me, Bn).

**α-(2,2-Dimethoxyethyl)-α-R^1^-*N-*(fluorenylmethyloxycarbonyl)glycine on Merrifield resins (13a–c, R^1^ = H, Me, Bn).** To 1.80–3.20 mmol of resin **12a**–**c** (R^1^ = H, Me, Bn) contained in a 50-mL SPPS vessel, 12–28 mL of absolute methanol, 4.4 equivalents of 1.0 M trimethylsilyl chloride in THF, and 25 equivalent of trimethylorthoformate were added under dry argon. The vessel was rocked overnight, and was then drained to leave a small volume of liquid over the resin. Diisopropylethylamine in absolute methanol (30 mL of a 3% *v*/*v* solution) was added. After shaking for 15 min, the vessel was drained, and the resin was washed with 3 × 20 mL of 3% DIEA in MeOH, 3 × 15 mL of NMP, and 8 × 15 mL of DCM and was dried overnight under a slow stream of dry nitrogen gas and then under high vacuum to give resin **13a**–**c** (R^1^ = H, Me, Bn).

**2-(Furan-2-carboxamido)-4,4-dimethoxy-2-R^1^-butanoic acid on Merrifield resins (16a–c, R^1^ = H, Me, Bn).** Fmoc acetal resin **13a**–**c** (R^1^ = H, Me, Bn, 100–157 μmol) contained in a 3.5-mL or 5-mL SPPS vessel was treated with 20% piperidine in NMP, and the vessel was gently agitated for 40–50 minutes at room temperature. The vessel was drained, and the resin was washed with 5 × 2–3 mL of NMP. To the deprotected resin was then added 5.2 eq of a 0.50-M solution of diisopropylethylamine (DIEA) in NMP, followed by 4.4 eq of a 0.50 M solution of 2-furoyl chloride in NMP. After 16–18 h, the vessel was drained, and the resin was washed twice each with NMP, 1:1 THF/MeOH (EtOH), and THF, and four times with DCM to give resin **16a**–**c** (R^1^ = H, Me, Bn).

**2-(Furan-2-carboxamido)-2-R^1^-4-oxobutanoic acid on Merrifield resins (17a–c, R^1^ = H, Me, Bn).** To 100–157 μmol of DCM-swelled resin **16a**–**c** (R^1^ = H, Me, Bn), 3 mL of TFA:DCM:water (4:4:1) was added. The mixture was gently agitated for 35 min at room temperature. The SPPS vessel was drained, and the resin was washed with 3 × 2 mL each with DCM and then THF to give aldehyde resin **17a**–**c** (R^1^ = H, Me, Bn).

**2-(Furan-2-carboxamido)-4-hydroxy-2-R^1^-butanoic acid on Merrifield resins (18a–c, R^1^ = H, Me, Bn).** To 100–157 μmol of resin **17a**–**c** (R^1^ = H, Me, Bn), 6 eq of a 0.90-M solution of sodium cyanoborohydride in 0.50 M of acetic acid in THF (for R^1^ = H, Me) or 7.5 eq of a suspension of sodium triacetoxyborohydride in 0.50 M of acetic acid in THF (for R^1^ = Bn) was added. The mixture was gently agitated for 6 h at room temperature. The vessel was drained, and the resin was washed with 3 × 2 mL each with THF, 30% aqueous THF, and THF to give the hydroxyl resin (**18a**–**c**, R^1^ = H, Me, Bn).

***N*-(3-R^1^-2-oxotetrahydrofuran-3-yl)furan-2-carboxamide (19a–c, R^1^ = H, Me, Bn).** Resin **18a**–**c** (100–157 μmol, R^1^ = H, Me, Bn) was washed with 2 × 2 mL of chlorobenzene, and was then treated with 2 mL of chlorobenzene followed by 8 eq of DIEA. The mixture was heated at 75 °C for 16 h. After cooling, the vessel was drained, and the resin was washed with 2 × 2 mL of DCM. The combined filtrates were evaporated to dryness to give a residue that was purified by silica gel chromatography: 

***N*-(2-oxotetrahydrofuran-3-yl)furan-2-carboxamide (19a, R^1^ = H)**, dichloromethane and dichloromethane/ethyl acetate mobile phases (94/6, 9/1); 5.7 mg (19% over nine steps) of **19a** as a white solid; ^1^H-NMR δ 2.28 (dq, *J* = 11.5 and 8.9 Hz, 1H), 2.92 (dddd, *J* = 12.6, 8.6, 5.8, and 1.0 Hz, 1H), 4.34 (ddd, *J* = 11.2, 9.2, and 5.8 Hz, 1H), 4.52 (dt, *J* = 9.7 and 0.8 Hz, 1H), 4.74 (ddd, *J* = 11.7, 8.6, and 6.3 Hz, 1H), 6.52 (dd, *J* = 3.5 and 1.8 Hz, 1H), 6.89 (br d, *J* = 3.9 Hz, 1H), 7.16 (dd, *J* = 3.5 and 0.5 Hz, 1H), 7.47 (dd, *J* = 1.6 and 0.6 Hz, 1H); ^13^C-NMR δ 30.6, 48.1, 66.1, 112.3, 115.3, 144.6, 146.9, 158.6, 175.1; HRMS (TOF ES^+^) *m*/*z* calculated for C_9_H_9_NO_4_ (M + Na) 218.0429; found 218.0429 (As shown in the Supplementary Materials).

***N*-(3-methyl-2-oxotetrahydrofuran-3-yl)furan-2-carboxamide (19b, R^1^ = Me)**, dichloromethane and dichloromethane/ethyl acetate mobile phases (9/1, 8/2) as mobile phases; 5.2 mg (25% over nine steps) of **19b** as a white solid; ^1^H-NMR δ 1.63 (s, 3H), 2.54 (ddd, *J* = 13.0, 7.0, and 2.3 Hz, 1H), 2.83 (dt, *J* = 12.9 and 9.7 Hz, 1H), 4.32 (dt, *J* = 9.6 and 7.0 Hz, 1H), 4.56 (dt, *J* = 9.3 and 2.3 Hz, 1H), 6.51 (dd, *J* = 3.5 and 1.7 Hz, 1H), 6.75 (br s, 1H), 7.13 (d, *J* = 3.5 Hz, 1H), 7.46 (d, *J* = 1.6 Hz, 1H); ^13^C-NMR δ 22.5, 35.1, 55.7, 65.6, 112.3, 115.1, 144.3, 147.2, 157.7, 177.3; HRMS (TOF ES^+^) *m*/*z* calculated for C_10_H_12_NO_4_ (M + H) 210.0761; found 210.0762.

***N*-(3-benzyl-2-oxotetrahydrofuran-3-yl)furan-2-carboxamide (19c, R^1^ = Bn)**, dichloromethane and dichloromethane/ethyl acetate (96/4, 9/1) as mobile phases; 4.3 mg (14% over nine steps) of **19c** as a white film; ^1^H-NMR δ 2.74–2.79 (m, 2H), 3.23 (d, *J* = 13.2 Hz, 1H), 3.29 (d, *J* = 13.2 Hz, 1H), 3.49 (dt, *J* = 9.2 and 7.6 Hz, 1H), 4.31 (dt, *J* = 8.9 and 3.2 Hz, 1H), 6.51 (dd, *J* = 3.5 and 1.7 Hz, 1H), 6.83 (br s, 1H), 7.14 (dd, *J* = 3.5 and 0.7 Hz, 1H), 7.26–7.28 (m, 2H), 7.32–7.35 (m, 3H), 7.45 (dd, *J* = 1.6 and 0.7 Hz, 1H), ^13^C-NMR δ 33.0, 42.0, 59.8, 65.9, 112.3, 115.2, 127.9, 128.9, 130.0, 133.6, 144.4, 147.2, 157.7, 179.8; HRMS (TOF ES^+^) *m*/*z* calculated for C_16_H_15_NO_4_Na (M + Na) 308.0899; found 308.0907.

**2-Benzyl-4,4-dimethoxy-2-(3,4,5-trimethoxybenzamido)butanoic acid on Merrifield resin (20c).** The Fmoc acetal resin **13c** (320 mg, 0.298 mmol) was placed in a 5-mL SPPS vessel, washed with DMF (2 × 3 mL), and then slowly washed with 20% piperidine/DMF (6 × 4 mL × 5 min), then with DMF (4 × 4 mL), with CH_2_Cl_2_ (3 × 4mL), and again with DMF (3 × 4 mL). The vessel was drained with a stream of argon, and the resin was treated with a freshly prepared solution of 3,4,5-trimethoxybenzoic acid (191 mg, 0.9 mmol, 3 eq) and HOBt-H_2_O (122 mg, 0.9 mmol, 3 eq) in 3 mL of a 1:1 CH_2_Cl_2_-DMF mixture. This was followed by the addition of diisopropylcarbodiimide (DIC, 140 μL, 114 mg, 0.9 mmol) and DIEA (78 μL). The vessel was capped and rotated at room temperature (RT) for 24 h. The solution was then drained from the vessel, and the resin was washed with DMF (3 × 4 mL), and CH_2_Cl_2_ (4 × 4 mL), and then dried with a stream of argon for 5 min to afford resin **20c**.

**Ethyl(*3R,6R,S,7aS*)-6-benzyl-5-oxo-6-(3,4,5-trimethoxybenzamido)hexahydropyrrolo[2,1-b]-thiazole-3-carboxylate (21c).** To 0.298 mmol of resin **20c**, a solution of KOAc (147.5 mg, 1.5 mmol, 5 eq) and L-Cys-OEt-HCl (111.4 mg, 0.6 mmol, 2 eq) in glacial AcOH (3 mL) was added. The vessel was capped, rotated for 15 min, and then transferred to an oven preheated to 90 °C. After 24 h, the mixture was cooled to RT, the solution was drained from the resin, and the resin was washed with THF (2 × 3 mL) and then with CH_2_Cl_2_ (3 × 3 mL). The combined filtrates were transferred to a separatory funnel containing brine (50 mL), water (50 mL), and CH_2_Cl_2_ (60 mL). After extraction, the phases were separated, and the organic phase was extracted with 10% KHCO_3_/H_2_O (80 mL). The organic layer was dried over Na_2_SO_4_, filtered, and concentrated in vacuo to afford a yellow oil (206 mg). This sample was purified by flash chromatography on silica gel (4.5 g; 45% EtOAc/hexanes). This gave the product diastereomeric thiazolidines **21c** as a colorless solidifying oil (41 mg; 27%). A portion (12.5 mg) was chromatographed on a 500-mg HyperSep SI column of silica gel to give 4.6 mg of **21c** as a 2:1 mixture of β:α; ^1^H-NMR (CDCl_3_) δ 1.31 and 1.32 (2t, 3H, *J* = 7.2 Hz and 7.2 Hz,), 2.64, (dd, 0.65H, *J* = 13.3 Hz and 7.3 Hz), 2.71 (dd, 0.37H, *J* = 14.6 Hz and 2.2 Hz), 2.93 (dd, 0.39H, *J* = 14.6 Hz and 7.7 Hz ), 3.21–3.49 (m, 5.14H), 3.86–3.87 (s, 3H), 3.86–3.87 (s, 2 × 3H), 4.20–4.31 (m, 2H), 4.78 (t, 0.68H, *J* = 6.9 Hz), 5.10 (dd, 0.38H, *J* = 8.1 Hz and 3.7 Hz 1H), 5.14 (dd, 0.64H, *J* = 7.2 Hz and 3.7 Hz), 5.41 (dd, 0.36H, *J* = 7.7 Hz and 2.2 Hz), 6.44 (bs, 0.39H), 6.60 (bs, 0.60H), 6.90 (s, 0.73H), 6.92 (s, 1.26H), 7.23–7.39 (m, 5.05H); ^13^C-NMR (CDCl_3_) δ 14.1, 14.2, 33.7, 35.4, 36.5, 41.7, 42.7, 42.9, 56.3, 56.4, 58.2, 58.4, 60.9, 62.06, 62.09, 63.0, 63.1, 65.4, 77.2, 104.47, 104.53, 127.5, 127.8, 128.5, 128.9, 129.3, 130.4, 130.6, 134.6, 135.0, 141.4, 153.20, 153.24, 166.1, 166.4, 168.8, 169.3, 173.0, 174.6. The diastereomers were separated on a 2.1 × 100 mm Agilent Eclipse XDB-C18 column using the mobile phase 65% MeOH, 35% 0.1% formic acid, and 5% acetonitrile at a flow rate of 0.3 µL/min, and their accurate masses were determined: 5.19 min, calculated for C_26_H_31_N_2_O_7_S (M + H) 515.1846; found 515.1848; 6.07 min, calculated for C_26_H_31_N_2_O_7_S (M + H) 515.1846; found 515.1848. 

**2-((*S*)-2-((((9H-Fluoren-9-yl)methoxy)carbonyl)amino)propanamido)-2-benzyl-4,4-dimethoxybutanoic acid on Merrifield resin (22c).** Resin **13c** (87.1 μmol) was treated with 2.5 mL of 20% piperidine in NMP for 30 min with gentle agitation. The vessel was drained, and the deprotected resin was washed with 5 × 3 mL of NMP. To this resin 262 mg (5.00 eq) of Fmoc-Ala anhydride in NMP in 3 mL of NMP was added. After 48 h, the vessel was drained, and the resin was washed with 4 × 3 mL each of NMP, THF, and NMP to give resin **22c**.

**2-Benzyl-2-((S)-2-(4-chlorobenzamido)propanamido)-4,4-dimethoxybutanoic acid on Merrifield resin (23c**)**.** Resin **22c** (87.1 μmol) was treated with 2.5 mL of 20% piperidine in NMP for 30 min with gentle agitation. The vessel was drained, and the deprotected resin was washed with 6 × 3 mL of NMP. The resin was then treated with 4.4 eq of 1.0 M solutions of 4-chlorobenzoyl chloride followed by 5.2 eq of DIEA. After 18 h, the vessels were drained, and the resin was washed with 4 × 3 mL each of NMP, 1:1 THF:MeOH, THF, and DCM to give resin **23c**.

**N-((*2S*)-1-((3-Benzyl-2-oxotetrahydrofuran-3-yl)amino)-1-oxopropan-2-yl)-4-chlorobenz-amide (24c).** Resin **23c** (87.1 μmol) was treated with 3 mL of 4:4:1 TFA:DCM:water for 35 min with gentle agitation. The vessel was drained, and the resin was washed with 5 × 3 mL of DCM and 3 × 3 mL of THF to afford the aldehyde resin. The resin was then treated with 3 mL of 0.50 M acetic acid in THF for 10–15 min. The vessel was drained, and the resin was treated with 1 mL of 0.50 M acetic acid in THF followed by 1.04 mL (6.3 eq) of a 0.53-M solution of sodium cyanoborohydride in 0.50 M of acetic acid in THF. The vessel was gently agitated for 6 h at room temperature; then, it was drained, and the resin was washed with 3 × 3 mL each of THF, 30% aqueous THF, and 4 × 3 mL of THF. The resin was dried under a stream of nitrogen gas, and was then washed with 3 mL of chlorobenzene. Chlorobenzene (3 mL) was then added, followed by 7.9 eq (690 μmol) of DIEA. The resin was then heated at 75 °C for 16 h. After cooling, the vessel was drained, and the filtrate was evaporated to dryness to yield 4.6 mg of crude **24c**. Separation of the diastereomers by reverse-phase HPLC chromatography gave 1.4 mg of the stereoisomer with the earlier retention time: ^1^H-NMR (methanol-*d*_4_) δ 1.49 (d, 3H, *J* = 7.2 Hz), 2.51 (ddd, 1H, *J* = 13.3 Hz, 8.2 Hz, and 3.0 Hz), 2.65–2.73 (m, 2H), 3.10 (d, 1H, *J* = 13.0 Hz), 3.15–3.21 (m, 1H), 3.20 (d, 1H, *J* = 13.0 Hz), 4.18 (ddd, 1H, *J* = 10.4 Hz, 8.9 Hz, and 3.0 Hz), 4.57 (q, 1H, *J* = 7.2 Hz), 7.30–7.35 (br m, 5H), 7.49 (d, 2H, *J* = 8.7 Hz), 7.87 (d, 2H, *J* = 8.8 Hz); ^13^C-NMR (methanol-*d*_4_) δ 16.2, 31.0, 41.5, 49.3, 60.0, 65.1, 127.4, 128.28, 128.33, 128.9, 130.0, 132.4, 133.6, 137.6, 167.6, 173.2, 177.7; HRMS (ES^+^) *m*/*z* calculated for C_21_H_21_ClN_3_O_4_S (M + Na) 423.1082; found 423.1085, and 1.5 mg of the stereoisomer with the later retention time: ^1^H-NMR (CDCl_3_) δ 1.46 (d, 3H, *J* = 7.0 Hz), 2.59 (ddd, 1H, *J* = 13.2 Hz, 7.6 Hz, and 4.9 Hz), 2.64–2.72 (m, 1H), 3.07 (d, 1H, *J* = 13.3 Hz), 3.16 (d, 1H, *J* = 13.2 Hz), 3.51 (m, 1H), 4.30 (dt, 1H, *J* = 9.5 Hz and 2.8 Hz), 4.67 (quintet, 1H, *J* = 7.1 Hz), 6.78 (br d, 1H, *J* = 7.3 Hz), 6.92 (br s, 1H), 7.21–7.24 (m, 2H), 7.28–7.31 (m, 3H), 7.42 (d, 2H, *J* = 8.6 Hz), 7.75 (d, 2H, *J* = 8.6 Hz); ^13^C-NMR (CDCl_3_) 17.6, 32.3, 41.9, 49.0, 59.8, 65.5, 128.0, 128.6, 128.90, 128.95, 130.1, 132.0, 133.4, 138.3, 166.6, 171.7, 176.7; HRMS (ES^+^) *m*/*z* calculated for C_21_H_21_ClN_3_O_4_S (M + Na) 423.1082; found 423.1085.

**2-Benzyl-4,4-dimethoxy-2-(4-methylbenzamido)butanoic acid on Merrifield resin (25c):** Resin **13c** (791 μmol) in a 50-mL SPPS vessel was washed with 3 × 20 mL of NMP, followed by 4 × 25 mL × five minutes of 20% piperidine in NMP and then with 3 × 20 mL of NMP. To the deprotected resin was added 20 mL of NMP, 722 μL (5.24 eq) of DIEA, and then 542 mg (4.4 eq) of *p*-toluoyl chloride. The vessel was placed on an orbital shaker, and after 40 h was drained, and the resin was washed with 3 × 20 mL of NMP, 2 × 20 mL of 1:1 THF:MeOH, 2 × 20 mL of THF, and 3 × 20 mL of DCM to give resin **25c**, which was dried under vacuum to afford a mass of 1.19 g.

**2-((*3R*)-6-Benzyl-6-(4-methylbenzamido)-5-oxohexahydropyrrolo[2,1-b]thiazole-3-carboxamido)-acetic acid (β-26c):** Resin **25c** (118 μmol), 56 mg (570 μmol, 4.8 eq) of potassium acetate and 42.9 mg (241 μmol, 2.0 eq) of Cys-Gly-OH in 1.5 mL of acetic acid contained in a 3.5-mL reaction vessel was heated at 90 °C for 24 h. After cooling, the vessel was drained, and the resin was washed with 3 × 2 mL of dichloromethane. The combined filtrates were evaporated, and the residue was partitioned between 25 mL of dichloromethane and 25 mL of 1.0 N HCl. After separation, the aqueous phase was extracted with 10 mL of dichloromethane, and the combined organics were washed with 20 mL of 1.0 N HCl and were dried (Na_2_SO_4_). Concentration gave 9.7 mg, which was triturated under 250 μL of dichloromethane to afford 3.7 mg (7% over 10 steps) of **β**-**26c** as a white solid; ^1^H-NMR (CD_3_OD) δ 2.64 (dd, *J* = 13.2 Hz and 6.6 Hz, 1H), δ 2.90 (dd, *J* = 13.3 Hz and 7.3 Hz, 1H), 3.15 (m, 2H), 3.28 (dd, *J* = 10.8 Hz and 3.4 Hz, 1H), 3.32 (10.8 Hz and 6.5 Hz, 1H), 3.76 (m, 2H), 4.10 (t, *J* = 7.0 Hz, 1H), 4.91 (dd, *J* = 6.5 Hz and 3.4 Hz, 1H), 6.55 (br t, *J* = 5.8 Hz, 1H), 7.19 (d, *J* = 8.0 Hz, 2H), 7.21–7.30 (m, 5H), 7.63 (d, *J* = 8.2 Hz, 2H), 8.37 (br s, 1H); ^13^C-NMR (CD_3_OD) δ 21.5, 34.6, 41.5, 41.8, 43.0, 61.7, 62.1, 67.4, 128.6, 128.8, 129.8, 130.2, 131.6, 132.3, 135.9, 143.8, 169.7, 170.9, 172.3, 176.2; HRMS (TOF ES^+^) *m*/*z* calculated for C_24_H_26_N_3_O_5_S (M + H) 468.1593; found 468.1609. Recrystallization from ethanol gave a sample for x-ray analysis.

**2-((*S*)-2-((((9H-Fluoren-9-yl)methoxy)carbonyl)amino)propanamido)-4,4-dimethoxy-2-methylbutanoic acid on Merrifield resin (28b).** A 25-mL SPPS vessel was charged with 773 mg (363 μmol) of resin **13b**, and was swelled in NMP for 30 minutes. The vessel was drained, and the resin was treated with 5 mL of 20% piperidine in NMP for 5 min. The vessel was drained, the resin was treated with 10 mL of 20% piperidine, and the vessel was rocked on an orbital shaker for 40 min. Then, it was drained, and the resin was washed with 5 × 10 mL of NMP. The deprotected resin was then treated with 859 mg (1.11 mmol, 3.05 eq) of Fmoc-Ala anhydride in 4.5 mL of NMP. The vessel was rocked for 42 h, drained, and the resin was washed with 6 × 15 mL of NMP to give resin **28b**.

**4,4-Dimethoxy-2-methyl-2-((S)-2-(4-nitrobenzamido)propanamido)butanoic acid on Merrifield resin (29b).** To 363 μmol of resin **28b** in a 25-mL SPPS vessel, 5 mL of 20% piperidine in NMP was added. After five min, the vessel was drained, and the resin was treated with 15 mL of 20% piperidine. The vessel was rocked for 40 min, drained, and the resin was washed with 6 × 15 mL of NMP. To the deprotected resin, 3.80 mL (1.90 mmol, 5.2 eq) of a 0.50-M solution of DIEA in NMP was added, followed by 3.21 mL (1.60 mmol, 4.4 eq) of a 0.50-M solution of 4-nitrobenzoyl chloride in NMP. The vessel was rocked for 18 h, drained, and the resin was washed with 2 × 15 mL of NMP, 3 × 15 mL of 1:1 THF:MeOH, 3 × 15 mL of THF, and 5 × 15 mL of DCM to give resin **29b**.

**Fmoc-Ala Anhydride, (Fmoc-Ala)_2_O.** A variation of the method of Izdebski and Pawlak [48] is described here. A 50-mL, three-neck, round-bottomed flask under argon was charged with 1.25 g (4.00 mmol) of Fmoc-Ala-OH. The flask was fitted with a thermometer and two rubber septa, and 11 mL of DCM was added via syringe. The mixture was treated with 1 mL of anhydrous DMF, and then cooled to 3 °C. To the mixture, 1.69 mL (252 mg, 2.00 mmol) of 1.0 M diisopropylcarbodiimide (DIC) in DCM was added dropwise via syringe over a two-minute period. After stirring at 3 °C for 30 minutes, the mixture was allowed to warm to room temperature, and was stirred an additional 10 minutes. The contents were filtered to afford 469 mg of crude anhydride (70 mol%) containing 30 mol% diisopropylurea (DIU), ^1^H-NMR 1.14 (d, 12H, *J* = 6.5 Hz, DIU), 1.49 (d, 6H, *J* = 7.0 Hz), 3.83 (octet, 2H, *J* = 6.6 Hz, DIU), 3.99 (br s, 2H, DIU), 4.21 (t, 2H, *J* = 7.0 Hz), 4.38–4.49 (2m, 6H), 5.26 (m, 2H), 7.31 (t, 4H, *J* =7.2 Hz), 7.40 (t, 4H, *J* =7.5 Hz), 7.58 (br t, 4H, *J* =6.1 Hz), 7.76 (d, 4H, *J* =7.6 Hz). Further drying gave 288 mg. The mother liquor was concentrated to a small volume and afforded an additional 463 mg (81 mol% anhydride) after drying. The combined yield of **(Fmoc-Ala)_2_O** was 56%.

**Methyl((*3R,7aS*)-6-methyl-6-((*S*)-2-(4-nitrobenzamido)propanamido)-5-oxohexahydropyrrolo[2,1-b]thiazole-3-carbonyl)-L-alaninate (30b).***Hydrolysis of dimethyl acetal functional group*: Resin **29b** (363 μmol) was treated with 13 mL of TFA:DCM:water (4:4:1) for 35 min at room temperature. The SPPS vessel was drained, and the resin was washed with 6 × 15 mL of DCM to give the aldehyde resin. *Preparation of Cys-Ala-OMe·TFA*: Boc-Cys(Trt)-Ala-OMe (262 mg, 478 μmol, 1.3 eq) was treated with 5 mL of trifluoroacetic acid (TFA): triethylsilane (TES) (97.5:2.5) for two hours at room temperature. The volatiles were removed in vacuo, and the residue was treated with 5 mL of 1:1 diethyl ether:hexanes, and then decanted. This was repeated again with 5 mL of 1:1 diethyl ether: hexanes. The residue was treated with 5 mL of diethyl ether to induce solidification, and then the ether was evaporated to give the TFA salt of Cys-Ala-OMe. This material was dissolved in 3 mL of acetic acid, and was added to the pre-formed aldehyde resin described above followed by 294 mg (3.00 mmol, 8.3 eq) of potassium acetate. The vessel was rocked overnight at room temperature and then drained. The resin was then washed with 2 × 5 mL of acetic acid. Acetic acid (5 mL) was added, and the vessel was heated at 55–60 °C for 48 h. After cooling, the vessel was drained, and the resin was washed with 2 × 5 mL of acetic acid. The three filtrates were combined and evaporated to dryness to give 65.6 mg of crude **30b**. The crude material was chromatographed on 2.0 g of normal phase silica gel 60 slurried in DCM. Elution with DCM, 98/2 DCM/MeOH, and 95/5 DCM/MeOH afforded 43.4 mg, which was then separated into its two major diastereomers by reverse-phase chromatography on a 5-micron, 21.4 × 250 mm, C18 column using 50/50 1:1 MeOH/MeCN (5 mM NH_4_OAc)/water (5 mM NH_4_OAc) to give 12.5 mg (7% over 13 steps) of **α-30b**: ^1^H-NMR 1.45 (d, *J* = 6.8 Hz, 3H), 1.45 (d, *J* = 7.2 Hz, 3H), 1.51 (s, 3H), 2.28 (dd, *J* = 14.3 Hz and 4.3 Hz, 1H), 2.80 (dd, *J* = 14.3 and 7.8 Hz, 1H), 3.56–3.64 (m, 2H), 3.63 (s, 3H), 4.66 (quintet, *J* = 7.6 Hz, 1H), 4.79–4.82 (m, 2H), 5.26 (dd, *J* = 7.8 Hz and 4.3 Hz, 1H), 7.25 (br s, 1H), 7.57 (br d, *J* = 7.8 Hz, 1H), 7.67 (br d, *J* = 8.2 Hz, 1H), 8.01 (d, *J* = 8.8 Hz, 2H), 8.26 (d, *J* = 8.8 Hz, 2H); ^13^C-NMR δ 17.0, 18.4, 25.6, 36.7, 38.7, 47.9, 49.0, 52.4, 57.5, 60.8, 62.9, 123.7, 128.6, 139.4, 149.8, 166.2, 168.3, 172.2, 172.6, 173.5; HRMS (TOF ES^+^) *m*/*z* calculated for C_22_H_27_N_5_O_8_SNa (M + Na) 544.1478; found 544.1493 and 16.2 mg (9% over 13 steps) of **α-30b**; ^1^H-NMR δ 1.38 (d, *J* = 7.2 Hz, 3H), 1.52 (d, *J* = 6.9 Hz, 3H), 1.60 (s, 3H), 2.65 (dd, *J* = 12.9 Hz and 7.3 Hz, 1H), 2.82 (dd, *J* = 12.9 Hz and 6.6 Hz, 1H), 3.48 (dd, *J* = 11.7 Hz and 7.4 Hz, 1H), 3.67 (dd, *J* = 11.8 Hz and 5.8 Hz, 1H), 3.76 (s, 3H), 4.50 (quintet, *J* = 7.1 Hz, 1H), 4.83 (quintet, *J* = 7.0 Hz, 1H), 4.95 (t, *J* = 6.6 Hz, 1H), 5.15 (t, *J* = 7.0 Hz, 1H), 7.06 (br s, 1H), 7.35 (br d, *J* = 8.6 Hz, 1H), 7.37 (br d, *J* = 8.0 Hz, 1H), 7.97 (d, *J* = 8.6 Hz, 2H), 8.27 (d, *J* = 8.6 Hz, 2H); ^13^C-NMR δ 18.0, 19.1, 23.6, 35.4, 41.7, 48.4, 49.3, 52.6, 58.8, 61.9, 62.6, 123.8, 128.4, 139.1, 149.8, 165.0, 168.0, 171.7, 172.9, 174.4; HRMS (TOF ES^+^) *m*/*z* calculated for C_22_H_27_N_5_O_8_SNa (M + Na) 544.1478; found 544.1478.

**Boc-Cys(Trt)-Ala-OMe (31).** A solution of 1.28 g (2.76 mmol) of Boc-Cys(Trt)-OH and 303 μL (2.76 mmol) of N-methylmorpholine in 7 mL of anhydrous DMF was prepared in a 20-mL scintillation vial fitted with a rubber septum and under dry argon gas. This solution was transferred via syringe to a 50-mL, three-neck, round-bottomed flask also under argon. The solution was then cooled in an ice/acetone bath (−9 °C) and treated via syringe with 358 μL (2.76 mmol) of isobutyl chloroformate over a 30 s interval. After three minutes, a solution of 385 mg (2.76 mmol) alanine methyl ester hydrochloride and 303 μL (2.76 mmol) of *N*-methylmorpholine in 7 mL of anhydrous DMF cooled in ice/acetone was added via syringe. The mixture was stirred at −9 °C for one hour, and then at room temperature for one hour. The reaction mixture was transferred to a 250-mL beaker and was evaporated with a stream of nitrogen overnight. The residue was partitioned between 75 mL of ethyl acetate and 30 mL of pH 2 buffer. The layers were separated, and the organic phase was washed with pH 2 buffer, saturated sodium bicarbonate, twice with water (to pH 7), and then dried over sodium sulfate. Concentration gave 1.38 g (89%) of **31**. LC/MS analysis (4.6 × 75 mm, 3.5-micron, Agilent Zorbax SB-C18 column, 70–100% solvent B over 20 min at 0.5 mL/min (solvent B: 1/1 MeCN/MeOH w 5 mM NH_4_OAc―solvent A: 5 mM NH_4_OAc) showed that no epimerization had occurred (R_t_ = 17.0 min, M + 59 = 607, 100%). A portion (518 mg) was recrystallized from 7 mL of methanol to afford 346 mg of **31**. ^1^H-NMR δ 1.35 (d, 3H, *J* = 7.1 Hz), 1.42 (s, 9H), 2.53 (br dd, 1H, *J* = 13.0 Hz and 5.0 Hz), 2.74 (br m, 1H), 3.70 (s, 3H), 3.82 (br s, 1H), 4.49 (quintet, 1H, *J* = 7.2 Hz), 4.77 (br s, 1H), 6.52 (br d, 1H, *J* = 5.6 Hz), 7.22 (t, 3H, *J* = 7.2 Hz), 7.29 (t, 6H, *J* = 7.5 Hz), 7.42 (d, 6H, *J* = 7.3 Hz); ^13^C-NMR δ 18.4, 28.3, 33.7, 48.1, 52.4, 53.5, 67.2, 80.3, 126.9, 128.1, 129.6, 144.4, 155.3,170.0, 172.8; HRMS (ESI) *m*/*z* calculated for C_31_H_36_N_2_O_5_SNa (M + Na) 571.2237; found 571.2239.

**Cys-Ala-OMe, trifluoroacetic acid salt (32).** Boc-Cys(Trt)-Ala-OMe (411 mg, 750 μmol) was treated with 6 mL of trifluoroacetic acid/triethylsilane (97.5/2.5) solution. The mixture was stirred at room temperature for two hours, and was then concentrated to a residue that was treated with 8 mL of 1:1 hexane:diethyl ether, and then decanted from the insoluble oil. This decantation was performed two additional times using 4 mL of 1:1 hexane:diethyl ether. The oil was then triturated under diethyl ether to induce solidification. The mixture was then evaporated to give **32** as a white solid, which was immediately used in the cyclitive cleavage.

**Compounds 33a**–**c, 34a, and 35a**–**c** were prepared according to the methods outlined in Scheme 11 and Scheme 12:

**(3*R*)-Ethyl 6-(4-chlorobenzamido)-5-oxohexahydropyrrolo[2,1-*b*]thiazole-3-carboxylate (α-33a and β-33a):** A mixture of 242 μmol of **15** (R^1^ = H, R^2^ = 4-ClPh) in 10 mL of dichloromethane contained in a 25-mL glass reaction vessel for 15 minutes was drained, and the resin was then treated with 10 mL of 4:4:1 TFA:CH_2_Cl_2_:water. The vessel was rocked for 35 minutes, drained, and the resin was washed with 5 × 3 mL of dichloromethane, and then with 2 × 3 mL of acetic acid. The now-formed aldehyde resin **7** (R^1^ = H, R^2^ = 4-ClPh) was then converted to **33a** using Method C by treatment with 340 μmol (1.40 equiv.) of polyvinylpyridine, 193 μmol of cysteine ethyl ester hydrochloride, and 5 mL of acetic acid. The vessel was rocked overnight at room temperature. LC/MS analysis indicated product formation. The contents were then heated/rocked at 55 °C for 24 h. The vessel was drained, and the filtrate was evaporated to dryness affording 41.5 mg, which was chromatographed on 3.0 g of silica gel eluting with toluene and 80/20 toluene/ethyl acetate to afford 5.9 mg (8% over 11 steps) of **α**-**33a** as an oil; ^1^H-NMR δ 1.32 (t, *J* = 7.2 Hz, 3H), 2.49 (dt, *J* = 14.9 Hz and 7.4 Hz, 1H), 2.86 (ddd, *J* = 14.2 Hz, 9,3 Hz, and 1.2 Hz, 1H), 3.40 (dd, *J* = 11.5 Hz and 4.6 Hz, 1H), 3.54 (dd, *J* = 11.5 Hz and 8.6 Hz, 1H), 4.26 (q, *J* = 7.1 Hz, 2H), 4.77 (ddd, *J* = 9.2 Hz, 8.3 Hz, and 6.3 Hz, 1H), (dd, *J* = 8.7 Hz and 4.6 Hz, 1H), 5.24 (dd, *J* = 7.1 Hz and 1.3 Hz, 1H), 6.92 (br d, *J* = 6.2 Hz, 1H), 7.40 (d, *J* = 8.5 Hz, 2H), 7.74 (d, *J* = 8.6 Hz, 2H); ^13^C-NMR δ 14.1, 30.1, 37.2, 51.6, 58.6, 62.3, 64.4, 128.6, 128.9, 131.6, 138.3, 166.6, 169.7, 175.1; HRMS (TOF ES^+^) *m*/*z* calculated for C_16_H_18_ClN_2_O_4_S (M + H) 369.0670; found 369.0668. Compound **β**-**33a** was eluted afterwards with 80/20 toluene/ethyl acetate to afford 5.6 mg (8% over 11 steps) as an oil; ^1^H-NMR δ 1.31 (t, *J* = 7.2 Hz, 3H), 2.11 (ddd, *J* = 12.7 Hz, 10.6 Hz, and 7.6 Hz, 1H), 3.35 (ddd, *J* = 12.8 Hz, 8.1 Hz, and 6.2 Hz, 1H), 3.42 (d, *J* = 5.7 Hz, 2H), 4.25 (m, 2H), 5.00 (dd, *J* = 10.5 Hz, 8.2 Hz, and 5.7 Hz, 1H), 5.17 (t, *J* = 5.4 Hz, 1H), 5.21 (dd, *J* = 7.4 Hz and 6.4 Hz, 1H), 6.95 (br d, *J* = 5.5 Hz, 1H), 7.38 (d, *J* = 8.6 Hz, 2H), 7.72 (d, *J* = 8.6 Hz, 2H); ^13^C-NMR δ 14.1, 35.3, 38.6, 54.0, 57.8, 62.2, 62.3, 128.6, 128.9, 131.6, 138.3, 166.4, 168.9, 171.5; HRMS (TOF ES^+^) *m*/*z* calculated for C_16_H_18_ClN_2_O_4_S (M + H) 369.0670; found 369.0672.

**(3*R*)-Ethyl6-(4-chlorobenzamido)-6-methyl-5-oxohexahydropyrrolo[2,1-b]thiazole-3-carboxylate (β-33b and α-33b):** A mixture of 133.5 μmol of **15** (R^1^ = Me, R^2^ = 4-ClPh) and 2.4 mL of 4:4:1 TFA:CH_2_Cl_2_:water contained in a 5-mL glass reaction vessel was rotated for 35 minutes, drained, and the resin was washed with 6 × 1.5 mL of dichloromethane. The resin was then dried under a stream of nitrogen, and then under vacuum. The now-formed aldehyde resin **7** (R^1^ = Me, R^2^ = 4-ClPh) was then converted to **33b** using Method B by treatment with 270 μmol (2.0 equiv) of cysteine ethyl ester hydrochloride in 2 mL of NMP, followed by 927 μmol (6.9 equiv.) of potassium acetate in 0.6 mL of acetic acid. The vessel was rotated for 24 h at room temperature, drained, and the resin was washed with 2 × 3 mL each of NMP, 5% DIEA in NMP, 5% DIEA in dichloromethane, and 3 × 3 mL of dichloromethane. The resin was dried under a stream of argon, and then treated with 2 mL of chlorobenzene. The contents were heated at 60 °C for 67 h, cooled, the vessel was drained, and the resin was washed with 3 × 4 mL of dichloromethane. The combined filtrates were evaporated to afford 1.7 mg. The resin was then heated at 75 °C in 3 mL of chlorobenzene for 50 h. The vessel was drained, and the resin was washed with 3 × 4 mL of dichloromethane. The combined filtrates were evaporated to afford 4.1 mg. This process was repeated at 75 °C for 40 h to afford 2.2 mg. The combined materials (1.7 mg, 4.1 mg, and 2.2 mg) were chromatographed on a Dynamax Microsorb 5-micron C18 column (21.4 × 250 mm) using a step gradient beginning with 6/4 1:1 MeCN/MeOH with 5.0 mM ammonium acetate/water with 5.0 mM ammonium acetate to afford 2.5 mg (5% over 11 steps) of **β**-**33b** as an oil; ^1^H-NMR δ 1.31 (t, *J* = 7.2 Hz, 3H), 1.69 (s, 3H), 2.67 (dd, *J* = 13.1 Hz and 7.4 Hz, 1H), 3.03 (dd, *J* = 13.0 Hz and 6.5 Hz, 1H), 3.40 (dd, *J* = 11.1 Hz and 3.0 Hz, 1H), 3.50 (dd, *J* = 11.2 Hz and 7.3 Hz, 1H), 4.24 (m, 2H), 5.19 (dd, *J* = 7.2 Hz and 3.0 Hz, 1H), 5.21 (t, *J* = 7.0 Hz, 1H), 6.63 (br s, 1H), 7.40 (d, *J* = 8.6 Hz, 2H), 7.72 (d, *J* = 8.6 Hz, 2H); ^13^C-NMR δ 14.1, 23.1, 35.3, 43.7, 57.9, 61.6, 61.7, 62.1, 128.5, 128.8, 132.1, 138.2, 165.5, 169.0, 173.9; HRMS (TOF ES^+^) *m*/*z* calculated for C_17_H_19_ClN_2_O_4_SNa (M + Na) 405.0652; found 405.0642. The resin was subjected to methoxide cleavage conditions by treating with 1.75 mL of anhydrous THF followed by 700 μL (660 mg, 3.00 mmol, 22 equiv.) of 25% sodium methoxide in methanol for 3 h. The vessel was drained under positive argon pressure while the resin was washed with 4 mL of absolute methanol. The combined filtrates were added to a rapidly stirred, cold mixture of 20 mL of dichloromethane and 20 mL of 1 N HCl. The layers were separated, the aqueous phase was extracted once with 10 mL of dichloromethane, and the combined organics were dried (Na_2_SO_4_). Concentration gave 13.9 mg of a mixture consisting primarily of the carboxylic acid of **33b**, which was esterified with ethyl iodide (300 mg) and 1,8-diazabicyclo[5.4.0]undec-7-ene (DBU) (648 μmol) overnight at room temperature. The mixture was concentrated to a residue that was partitioned between ethyl acetate/5% citric acid/brine. The organic phase was washed with 5% citric acid and dried (MgSO_4_). Concentration gave a wet residue that was diluted with dichloromethane and then dried with sodium sulfate. Concentration gave 8.7 mg, which was chromatographed on a Dynamax Microsorb 5-micron C18 column (21.4 × 250 mm) using a step gradient beginning with 1/1 1:1 MeCN/MeOH with 5.0 mM ammonium acetate/water with 5.0 mM ammonium acetate to afford 2.1 mg (4% over 13 steps) of **α**-**33b** as an oil; ^1^H-NMR δ 1.33 and 1.34 (2t, *J* = 7.2 Hz and 7.2 Hz, 3H), 1.65 (s, 3H), 2.51 (d, *J* = 14.5 Hz, 1H), 3.10 (dd, *J* = 14.4 Hz and 7.8 Hz, 1H), 3.41 (dd, *J* = 11.4 Hz and 4.3 Hz, 1H), 3.48 (dd, *J* = 11.3 Hz and 8.4 Hz, 1H), 4.26 (m, 2H), 5.07 (dd, *J* = 8.2 Hz and 4.2 Hz, 1H), 5.34 (d, *J* = 8.7 Hz, 1H), 6.40 (br s, 1H), 7.40 (d, *J* = 8.6 Hz, 2H), 7.71 (d, *J* = 8.6 Hz, 2H); ^13^C-NMR δ 14.1, 25.8, 36.2, 36.5, 58.3, 59.6, 62.1, 63.3, 128.5, 128.9, 132.0, 138.2, 165.8, 169.4, 176.0; HRMS (TOF ES^+^) *m/z* calculated for C_17_H_19_ClN_2_O_4_SNa (M + Na) 405.0652; found 405.0667.

**(3*R*)-Ethyl6-benzyl-6-(4-chlorobenzamido)-5-oxohexahydropyrrolo[2,1-b]thiazole-3-carboxylate (β-33c and α-33c):** A mixture of 260 μmol of **15** (R^1^ = benzyl, R^2^ = 4-ClPh) and 2.4 mL of 4:4:1 TFA:CH_2_Cl_2_:water contained in a 5-mL glass reaction vessel was rotated for 35 minutes, drained, and the resin was washed with 6 × 1.5 mL of dichloromethane. The resin was then dried under a stream of nitrogen, and then under vacuum. The now-formed aldehyde resin **7** (R^1^ = benzyl, R^2^ = 4-ClPh) was then converted to **33c** using Method F by treatment with 1.56 mL (1.56 mmol, 6.00 equivalent) of 1.0 M potassium acetate in acetic acid followed by 1.04 mL (0.520 mmol, 2.00 equivalent) of cysteine ethyl ester hydrochloride in acetic acid. After rotation at room temperature for 18 h, the vessel was drained, and the resin was washed once with 3 mL of THF, and then with 4 × 2.5 mL of 5% DIEA in dichloromethane, and then with 4 × 2 mL of dichloromethane. The resin was dried *in vacuo*, and then treated with 3 mL of chlorobenzene followed by 0.270 mL (200 mg, 1.55 mmol, 6.0 equiv.) of DIEA. The mixture was heated at 55 °C for 24 h, the vessel was drained, and the resin washed with 2 × 3 mL of dichloromethane. Evaporation of the combined filtrates gave only 5.0 mg. The resin was then treated with 3 mL of acetic acid, and the vessel was heated at 75 °C for 40 h. After cooling, the vessel was drained, and the resin was washed with 2 × 2 mL of acetic acid. The combined filtrates were evaporated to give 46.6 mg, which was triturated under 500 μL of warm acetonitrile to afford 15.6 mg (13% over 11 steps) of **β**-**33c** as a white solid; ^1^H-NMR δ 1.31 (t, *J* = 7.2 Hz, 3H), 2.61 (dd, *J* = 13.4 Hz and 7.4 Hz, 1H), 3.23 (d, *J* = 13.3 Hz, 1H), 3.32 (dd, *J* = 13.4 Hz and 6.4 Hz, 1H), 3.37 (dd, *J* = 11.3 Hz and 3.6 Hz, 1H), 3.44 (dd, *J* = 11.3 Hz and 7.5 Hz, 1H), 3.45 (d, *J* = 13.4 Hz, 1H), 4.25 (m, 2H), 4.75 (t, *J* = 6.8 Hz, 1H), 5.14 (dd, *J* = 7.1 Hz and 3.6 Hz, 1H), 6.66 (br s, 1H), 7.21 (m, 2H), 7.28–7.31 (m, 3H), 7.39 (d, *J* = 8.6 Hz, 2H), 7.67 (d, *J* = 8.5 Hz, 2H); ^13^C-NMR δ 14.2, 35.3, 41.5, 42.9, 58.1, 62.06, 62.07, 65.5, 127.5, 128.4, 128.5, 128.9, 130.2, 132.2, 134.4, 138.2, 165.6, 168.8, 172.9; HRMS (TOF ES^+^) *m*/*z* calculated for C_23_H_24_ClN_2_O_4_S (M + H) 459.1140; found 459.1147. The filtrate was evaporated to a solid that was taken up in 650 μL of 1:1 MeCN/MeOH, decanted with syringe, and the filtrate was diluted with 350 μL of water. The mixture was filtered through a 0.45 micron filter, and was injected onto a Dynamax Microsorb 5-micron C_18_ column (21.4 × 250 mm) and chromatographed using a step gradient beginning with 65/35 1:1 MeCN/MeOH with 5.0 mM ammonium acetate/water with 5.0 mM ammonium acetate to afford 1.3 mg (1% over 11 steps) of **α**-**33c** as a film; ^1^H-NMR δ 1.31 (t, *J* = 7.1 Hz, 3H), 2.70 (dd, *J* = 14.6 Hz and 2.3 Hz, 1H), 2.93 (dd, *J* = 14.6 Hz and 7.7 Hz, 1H), 3.19 (d, *J* = 11.5 Hz, 1H), 3.21 (d, *J* = 11.2 Hz, 1H), 3.31 (d, *J* = 11.5 Hz, 1H), 3.33 (dd, *J* = 11.0 Hz and 3.6 Hz, 1H), 4.25 (m, 2H), 5.08 (dd, *J* = 8.1 Hz and 3.5 Hz, 1H), 5.39 (dd, *J* = 7.8 Hz and 2.3 Hz, 1H), 6.47 (br s, 1H), 7.27–7.34 (m, 5H), 7.39 (d, *J* = 8.5 Hz, 2H), 7.63 (d, *J*= 8.6 Hz, 2H); ^13^C-NMR δ 14.1, 33.6, 36.4, 42.8, 58.2, 62.1, 63.0, 63.2, 127.8, 128.4, 128.9, 129.0, 130.6, 132.0, 134.7, 138.2, 165.5, 169.3, 174.3; HRMS (TOF ES^+^) *m*/*z* calculated for C_23_H_24_ClN_2_O_4_S (M + H) 459.1140; found 459.1146.

**(2*S*)-Methyl2-((3*R*)-6-(4-fluorobenzamido)-5-oxohexahydropyrrolo[2,1-b]thiazole-3-carboxamido)-4-methylpentanoate (β-34a and α-34a):** Prepared using Method C and Scheme 12 *Preparation of Cys-Leu-OMe*. Fmoc-Leu-Wang resin (559 μmol) contained in an SPPS vessel was washed four times with NMP, and was then treated with 6 × 8 mL × 5 min 20% (*v*/*v*) piperidine in NMP. To the deprotected resin was then added a mixture of Boc-Cys(Trt)-OH (3.0 equivalent), DIEA (6.0 equivalent), *N*,*N*,*N′*,*N′*-tetramethyl-O-(1H-benzotriazol-1-yl)uronium hexafluorophosphate, and O-(benzotriazol-1-yl)-*N*,*N*,*N′*,*N′*-tetramethyluronium hexafluorophosphate (HBTU, 3 equivalent) in 6 mL of 85/15 DCM/DMF. The vessel was rocked for 20 h, drained, and the resin was washed three times each with DMF and 1:1 THF:MeOH, and then four times with methanol to give Boc-Cys(Trt)-Leu on Wang resin. The resin was then treated with 12 mL 30% (*v*/*v*) triethylamine in methanol, and the vessel was placed in an oven at 55–60 °C for 48 h. The contents were allowed to cool, the vessel was drained, and the resin was washed with 2 × 20 mL of methanol. The combined filtrates were then concentrated to give 287 mg of crude Boc-Cys(Trt)-OMe; ^1^H-NMR δ 0.89 (d, 3H, *J* = 6.2 Hz), 0.90 (d, 3H, *J* = 6.1 Hz), 1.42 (s, 9H), 1.51 (m, 1H), 1.57–1.63 (m, 2H), 2.50 (dd, 1H, *J* = 13.0 Hz and 5.0 Hz), 2.75 (br m, 1H), 3.68 (s, 3H), 3.82 (br m), 4.54 (br m), 4.74 (br s), 6.41 (br s), 7.22 (t, 3H, *J* = 7.2 Hz), 7.30 (t, 6H, *J* = 7.7 Hz), 7.42 (d, 6H, *J* = 7.7 Hz). Chromatography on 2.0 g of silica gel afforded 227.5 mg (57%) of purified Boc-Cys(Trt)-OMe. To 7 mL of trifluoroacetic acid (TFA):triethylsilane (TES) (97.5:2.5) under argon was added 175 mg (297 μmol) of purified Boc-Cys(Trt)-OMe. The mixture was stirred at 35 °C for one hour, and was then concentrated to a residue that was triturated twice with 15 mL of 1:1 hexanes:diethyl ether and decanted. The residue was then dried under vacuum to give a white powder that was dissolved in 2.7 mL of acetic acid and used immediately in the cyclitive cleavage, which is described as follows.

A 3.5-mL reaction vessel was charged with 106 μmol of resin **7** (R^1^ = H, R^2^ = 4-FPh), 19 mg (181 μmol, 1.7 equivalent) of polyvinylpyridine, 0.8 mL (85 μmol, 0.80 equivalent) of a 0.106 M solution of Cys-Leu-OMe in acetic acid, and 0.8 mL of acetic acid, and heated at 55 °C for 20 h. The vessel was drained, the resin was washed with 2 × 2 mL of acetic acid, and the combined filtrates were evaporated to give 23 mg. Further exposure (72 h) to 55 °C gave an additional 4.1 mg. Both quantities were combined and chromatographed on 1.0 g of silica gel. The higher R_f_ material, **α**-**34a**, was eluted with 1/1 hexanes/ethyl acetate to afford 5.7 mg, while the lower R_f_ material, **β**-**34a**, was eluted with 1/2 hexanes/ethyl acetate to afford 6.4 mg. Each of these materials was separately further purified by reverse-phase HPLC on a Dynamax Microsorb 5-micron C18 column (21.4 × 250 mm) using step gradients of 1:1 MeCN/MeOH with 5.0 mM ammonium acetate/water with 5.0 mM ammonium acetate to afford 4.6 mg (12% over seven steps) of **α**-**34a** as a film; ^1^H-NMR δ 0.92 and 0.93 (2d, *J* = 6.3 Hz and 6.3 Hz, 6H), 1.54–1.64 (m, 2H), 1.65–1.69 (m, 1H), 2.14 (ddd, *J* = 12.8 Hz, 10.8 Hz, and 7.6 Hz, 1H), 3.35 (ddd, 12.7 Hz, 8.4 Hz, and 6.3 Hz, 1H), 3.36 (dd, *J* = 12.0 Hz and 6.3 Hz, 1H), 3.75 (s, 3H), 3.79 (dd, *J* = 12.0 Hz and 6.4 Hz, 1H), 4.55–4.60 (m, 1H), 4.88 (t, *J* = 6.8 Hz, 1H), 5.09 (dd, *J* = 8.4 Hz and 6.1 Hz, 1H), 5.11 (dd, *J* = 7.3 Hz and 6.5 Hz, 1H), 6.84 (br d, *J* = 5.9 Hz, 1H), 6.95 (br d, *J* = 7.9 Hz, 1H), 7.12 (t, *J* = 8.6 Hz, 2H), 7.82 (dd, *J* = 8.7 Hz and 5.2 Hz, 2H); ^13^C-NMR δ 22.0, 22.7, 25.0, 34.8, 37.7, 41.3, 51.3, 52.5, 54.1, 59.3, 63.1, 115.7 (d, ^2^*J_CF_* = 22.0 Hz), 129.3 (d, ^4^*J_CF_* = 3.1 Hz), 129.5 (d, ^3^*J_CF_* = 9.1 Hz), 165.0 (d, ^1^*J_CF_* = 253 Hz), 166.4, 167.6, 172.8, 173.1; HRMS (TOF ES^+^) *m*/*z* calculated for C_21_H_27_FN_3_O_5_S (M + H) 452.1650; found 452.1654. **β**-**34a**: 4.7 mg (12% over 7 steps); ^1^H-NMR δ 0.97 and 0.98 (2d, *J* = 6.7 Hz and 6.6 Hz, 6H), 1.72–1.79 (m, 3H), 2.56 (ddd, *J* = 14.3 Hz, 7.5 Hz, and 4.7 Hz, 1H), 2.65 (ddd, *J* = 14.5 Hz, 10.5 Hz, and 4.2 Hz, 1H), 3.60 (dd, *J* = 11.6 Hz and 8.6 Hz, 1H), 3.69 (dd, *J* = 11.7 Hz and 4.9 Hz, 1H), 3.76 (s, 3H), 4.36 (ddd, *J* = 11.1 Hz, 6.7 Hz, and 4.9 Hz, 1H), 4.53–4.57 (m, 1H), 4.93 (dd, *J* = 8.5 Hz and 4.8 Hz, 1H), 5.31 (dd, *J* = 7.6 Hz and 4.2 Hz, 1H), 6.98 (t, *J* = 8.6 Hz, 2H), 7.47 (br d, *J* = 7.6 Hz, 1H), 7.63 (dd, *J* = 8.7 Hz and 5.2 Hz, 2H), 8.16 (br d, *J* = 6.7 Hz, 1H); ^13^C-NMR δ 21.8, 22.8, 25.0, 32.1, 36.5, 40.6, 51.4, 52.3, 54.8, 57.8, 64.7, 115.5 (d, ^2^*J_CF_ =* 22.0 Hz), 128.3 (d, ^4^*J_CF_* = 3.0 Hz), 129.5 (d, ^3^*J_CF_* = 9.2 Hz), 165.0 (d, ^1^*J_CF_* = 253 Hz), 166.4, 168.8, 172.6, 172.9; HRMS (TOF ES^+^) *m*/*z* calculated for C_21_H_27_FN_3_O_5_S (M + H) 452.1650; found 452.1656.

**(2*S*)-Methyl2-((3*R*)-6-(4-chlorobenzamido)-5-oxohexahydropyrrolo[2,1-b]thiazole-3-carboxamido)propanoate (α-35a and β-35a):** Separated by reverse-phase HPLC on a Dynamax Microsorb 5-micron C18 column (21.4 × 250 mm) using a step gradient beginning with 60/40 of 1:1 MeCN/MeOH with 5.0 mM of ammonium acetate/water with 5.0 mM ammonium acetate to afford 6.3 mg (6% over 11 steps) of **α**-**35a**; ^1^H-NMR δ 1.53 (d, *J* = 7.3 Hz, 3H), 2.56 (ddd, *J =* 14.3 Hz, 7.6 Hz, and 4.9 Hz, 1H), 2.65 (ddd, *J =* 14.5 Hz, 10.5 Hz, and 4.1 Hz, 1H), 3.60 (dd, *J* = 11.6 Hz and 8.6 Hz, 1H), 3.69 (dd, *J* = 11.6 Hz and 4.7 Hz, 1H), 3.80 (s, 3H), 4.37 (ddd, *J =* 10.8 Hz, 6.7 Hz, and 4.9 Hz, 1H), 4.56 (quintet, *J* = 7.2 Hz, 1H), 4.95 (dd, *J* = 8.7 Hz and 4.8 Hz, 1H), 5.32 (dd, *J* = 7.6 Hz and 4.1 Hz, 1H), 7.26 (d, *J* = 8.6 Hz, 2H), 7.50 (d, *J* = 8.6 Hz, 2H), 7.58 (br d, *J* = 7.1 Hz, 1H), 8.42 (br d, *J* = 6.8 Hz, 1H); ^13^C-NMR δ 17.5, 31.8, 36.4, 48.6, 52.5, 54.7, 57.8, 64.6, 128.5, 128.7, 130.3, 138.2, 166.5, 168.5, 172.8, 172.9; HRMS (TOF ES^+^) *m*/*z* calculated for C_18_H_20_ClN_3_O_5_SNa (M + Na) 448.0710; found 448.0725 and 4.6 mg (4% over 11 steps) of **β**-**35a**; ^1^H-NMR 1.42 (d, *J* = 7.2 Hz, 3H), 2.14 (ddd, *J =* 12.9 Hz, 10.7 Hz, and 7.5 Hz, 1H), 3.35 (dd, *J* = 8.3 Hz and 6.3 Hz, 1H), 3.38 (dd, *J* = 12.0 Hz and 7.2 Hz, 1H), 3.77 (s, 3H), 3.78 (dd, *J* = 12.1 Hz and 6.4 Hz, 1H), 4.54 (quintet, *J* = 7.1 Hz, 1H), 4.86 (t, *J* = 6.8 Hz, 1H), 5.08 (ddd, *J =* 10.6 Hz, 8.5 Hz and 5.9 Hz, 1H), 5.13 (t, *J* = 6.8 Hz, 1H), 6.74 (br d, *J* = 5.6 Hz, 1H), 7.10 (br d, *J* = 6.9 Hz, 1H), 7.42 (d, *J* = 8.5 Hz, 2H), 7.74 (d, *J* = 8.5 Hz, 2H); ^13^C-NMR δ 18.2, 34.9, 37.6, 48.6, 52.7, 54.2, 59.3, 63.1, 128.6, 129.0, 131.5, 138.4, 166.4, 167.5, 172.8, 173.0; HRMS (TOF ES^+^) *m*/*z* calculated for C_18_H_20_ClN_3_O_5_SNa (M + Na) 448.0710; found 448.0710.

**(2*S*)-Methyl2-((3*R*)-6-(4-chlorobenzamido)-6-methyl-5-oxohexahydro-pyrrolo[2,1-b]thiazole-3carboxamido)propanoate (β-35b and α-35b):** Separated on 2.0 g of silica gel 60 using hexanes/ethyl acetate eluents (40/60 and 1/2) to afford 10.3 mg (9% over 11 steps) of **β-35b**; ^1^H-NMR δ 1.41 (d, *J* = 7.1 Hz, 3H), 1.69 (s, 3H), 2.73 (dd, *J* = 13.1 Hz and 7.3 Hz, 1H), 2.99 (dd, *J* = 13.1 Hz and 6.6 Hz, 1H), 3.47 (dd, *J* = 12.9 Hz and 7.3 Hz, 1H), 3.73 (dd, *J* = 11.9 Hz and 6.0 Hz, 1H), 3.76 (s, 3H), 4.51 (quintet, *J* = 7.1 Hz, 1H), 4.89 (dd, *J* = 6.9 Hz and 6.3 Hz, 1H), 5.13 (t, *J* = 6.9 Hz, 1H), 6.71 (br s, 1H), 7.22 (br d, *J* = 6.8 Hz, 1H), 7.40 (d, *J* = 8.6 Hz, 2H), 7.73 (d, *J* = 8.6 Hz, 2H); ^13^C-NMR δ 19.2, 23.5, 35.0, 42.2, 48.5, 52.6, 59.2, 62.0, 62.7, 128.5, 128.9, 131.9, 138.3, 165.5, 167.8, 172.9, 175.4; HRMS (TOF ES^+^) *m*/*z* calculated for C_19_H_23_ClN_3_O_5_S (M + H) 440.1041; found 440.1039 and 10.1 mg (9% over 11 steps) of **α-35b**; ^1^H-NMR δ 1.51 (d, *J* = 7.3 Hz, 3H), 1.64 (s, 3H), 2.34 (dd, *J* = 14.3 Hz and 3.8 Hz, 1H), 2.88 (dd, *J* = 14.3 and 7.9 Hz, 1H), 3.55 (dd, *J* = 11.6 Hz and 8.6 Hz, 1H), 3.68 (dd, *J* = 11.7 Hz and 4.8 Hz, 1H), 3.69 (s, 3H), 4.54 (quintet, *J* = 7.2 Hz, 1H), 4.93 (dd, *J* = 8.6 Hz and 4.8 Hz, 1H), 5.26 (dd, *J* = 7.9 Hz and 3.8 Hz, 1H), 6.59 (br s, 1H), 7.40 (d, *J* = 8.6 Hz, 2H), 7.62 (br d, *J* = 7.1 Hz, 1H), 7.67 (d, *J* = 8.6 Hz, 2H); ^13^C-NMR δ 17.6, 26.2, 36.0, 38.7, 48.5, 52.4, 57.8, 60.5, 62.5, 128.5, 129.0, 131.4, 138.5, 166.4, 168.7, 172.5, 173.1; HRMS (TOF ES^+^) *m*/*z* calculated for C_19_H_23_ClN_3_O_5_S (M + H) 440.1041; found 440.1044.

**(2*S*)-Methyl2-((3*R*)-6-benzyl-6-(4-chlorobenzamido)-5-oxohexahydro-pyrrolo[2,1-b]thiazole-3carboxamido)propanoate (β-35c and α-35c):** Separated on two 2.0 g of silica gel 60 using hexanes/ethyl acetate eluents (70/30, 60/40, 55/45, 1/1) to afford 10.3 mg (8% over 11 steps) of **β-35c**; ^1^H-NMR δ 1.43 (d, *J* = 7.2 Hz, 3H), 2.69 (dd, *J* = 13.7 Hz and 7.1 Hz, 1H), 3.27 (dd, *J* = 13.7 Hz and 6.7 Hz, 1H), 3.30 (d, *J* = 12.9, 1H), 3.34 (dd, *J* = 8.6 Hz and 7.5 Hz, 1H), 3.35 (d, *J* = 13.1 Hz, 1H), 3.64 (dd, *J* = 11.8 Hz and 5.8 Hz, 1H), 3.80 (s, 3H), 4.25 (t, *J* = 6.8 Hz, 1H), 4.59 (quintet, *J* = 7.3 Hz, 1H), 4.85 (t, *J* = 6.4 Hz, 1H), 6.73 (br s, 1H), 6.94 (br d, *J* = 7.4 Hz, 1H), 7.23–7.25 (m, 2H), 7.29–7.31 (m, 3H), 7.41 (d, *J* = 8.5 Hz, 2H), 7.71 (d, *J* = 8.5 Hz, 2H); ^13^C-NMR δ 18.2, 34.6, 41.5, 42.2, 48.3, 52.6, 60.1, 62.7, 66.0, 127.9, 128.5, 128.7, 128.9, 130.1, 132.0, 134.3, 138.3, 165.5, 167.3, 172.7, 174.9; HRMS (TOF ES^+^) *m*/*z* calculated for C_25_H_26_ClN_3_O_5_SNa (M + Na) 538.1179; found 538.11 and 5.1 mg (3% over 11 steps) of **α-35c** (90% by-NMR); ^1^H-NMR δ 1.50 (d, *J* = 7.2 Hz, 3H), 2.62 (dd, *J* = 14.3 and 7.6 Hz, 1H), 2.68 (dd, *J* = 14.3 and 4.5 Hz, 1H), 3.19 (d, *J* = 13.6 Hz, 1H), 3.27 (d, *J* = 13.5 Hz, 1H), 3.35 (dd, *J* = 11.3 Hz and 8.4 Hz, 1H), 3.63 (dd, *J* = 11.4 Hz and 4.3 Hz, 1H), 3.71 (s, 3H), 4.53 (quintet, *J* = 7.2 Hz, 1H), 4.94 (dd, *J* = 8.4 Hz and 4.3 Hz, 1H), 5.25 (dd, *J* = 7.6 Hz and 4.5 Hz, 1H), 6.56 (br s, 1H), 7.31–7.33 (m, 2H), 7.37–7.43 (m, 5H), 7.56 (d, *J* = 8.6 Hz, 2H), 7.68 (br d, *J* = 7.2 Hz, 1H); ^13^C-NMR δ 17.5, 35.8, 37.0, 43.5, 48.6, 52.3, 57.9, 62.5, 63.9, 128.2, 128.3, 129.0, 129.3, 130.2, 131.6, 134.0, 138.6, 165.9, 168.5, 171.8, 172.5; HRMS (TOF ES^+^) *m*/*z* calculated for C_25_H_27_ClN_3_O_5_S (M + H) 516.1354; found 516.1355.

**General procedure for the preparation of 36a**–**c and 30b**,**c (Method D cleavage).** According to Scheme 10, 250 μmol of resin **13a**–**c** contained in a SPPS vessel was treated with 20% piperidine in NMP at room temperature for 40 minutes with gentle agitation. The vessel was drained, and the resin was washed with 6 × 4 mL of NMP. To the deprotected resin, Fmoc-Ala, HOBt, and DIC (5 eq each) were added in NMP for R^1^ = H or (Fmoc-Ala)_2_O (3 eq) in NMP for R^1^ = Me and Bn. The vessel was agitated for 18–42 h, drained, and the resin was washed with 6 × 6 mL of NMP to give resin **22a**–**c**. Resin **22a**–**c** was treated with 20% piperidine in NMP at room temperature for 40 minutes with gentle agitation. The vessel was drained, and the resin was washed with 6 × 4 mL of NMP. To the deprotected resin 4-chlorobenzoyl chloride (4.4 eq) in NMP was added, followed by DIEA (5.2 eq) in NMP. The vessel was rocked for 18 h to 24 h, drained, and the resin was washed with 3 × 6 mL of NMP, 3 × 6 mL of 1:1 THF:MeOH, 3 × 6 mL of THF, and 5 × 6 mL of DCM. The resin was treated with 5 mL of 4:4:1 TFA:CH_2_Cl_2_:water for 35 min at room temperature. The mixture was filtered, and the resin was washed with 5 × 2 mL of dichloromethane, and then dried under a stream of nitrogen. To the resulting resin **38a**–**c**, 1.0–1.3 equivalents of a 0.25-M solution of **32** in acetic acid was added, followed by 6.2–8.6 equivalents of a 1.6-M solution of potassium acetate in acetic acid. The vessel was rotated at room temperature overnight (16–22 h) and was then drained, and the resin was washed with acetic acid. The combined filtrates were evaporated to a residue that was taken up in dichloromethane and washed two times with saturated sodium bicarbonate and dried (MgSO_4_). Concentration gave a crude sample that was analyzed by LC/MS and quantitative NMR. The resin was then treated with 3 mL of acetic acid, and was heated at 55 °C for 24 h. The vessel was drained, the resin was washed with acetic acid, and the combined filtrates were evaporated to a residue. A further exposure of the resin/acetic acid to 55 °C for 24 h resulted in a minor amount of cyclitive cleavage product. The residues were combined, and the diastereomers were separated by normal phase or reverse-phase chromatography.

(**2S)-Methyl2-((3R)-6-((S)-2-(4-chlorobenzamido)propanamido)-5-oxohexahydropyrrolo[2,1-b]thiazole-3-carboxamido)propanoate (β-36a and α-36a):** Separated by chromatography on 2.0 g of silica gel 60 using CH_2_Cl_2_ and CH_2_Cl_2_/MeOH (98/2, 97/3, and 95/5) to afford 4.1 mg (4% over 13 steps) of **β-36a**; ^1^H-NMR δ 1.37 (d, *J* = 7.2 Hz, 3H), 1.49 (d, *J* = 7.0 Hz, 3H), 2.07 (ddd, *J* = 12.8 Hz, 10.8 Hz, and 7.4 Hz, 1H), 3.14 (ddd, *J* = 12.9 Hz, 8.5 Hz, and 6.3 Hz, 1H), 3.33 (dd, *J* = 11.8 Hz and 7.2 Hz, 1H), 3.71 (dd, *J* = 11.8 Hz and 5.8 Hz, 1H), 3.74 (s, 3H), 4.51 (quintet, *J* = 7.2 Hz, 1H), 4.78 (quintet, *J* = 7.2 Hz, 1H), 4.84–4.89 (m, 2H), 5.06 (t, *J* = 6.8 Hz, 1H), 6.95 (br d, *J* = 7.3 Hz, 1H), 7.14 (br d, *J* = 6.3 Hz, 1H), 7.20 (br d, *J* = 7.1 Hz, 1H), 7.41 (d, *J* = 8.5 Hz, 2H), 7.75 (d, *J* = 8.5 Hz, 1H); ^13^C-NMR δ 18.0, 18.3, 34.8, 37.0, 48.5, 49.0, 52.6, 53.7, 59.1, 62.7, 128.6, 128.9, 132.0, 138.3, 166.4, 167.7, 172.5, 172.6, 172.9; HRMS (TOF ES^+^) *m*/*z* calculated for C_21_H_26_ClN_4_O_6_S (M + H) 497.1256; found 497.1260 and 2.7 mg (3% over 13 steps) of **α-36a**; ^1^H-NMR δ 1.44 (d, *J* = 7.1 Hz, 3H), 1.46 (d, *J* = 7.3 Hz, 3H), 2.48 (ddd, *J* = 14.3 Hz, 7.3 Hz, and 4.8 Hz, 1H), 2.56 (ddd, *J* = 14.4 Hz, 10.2 Hz, and 4.3 Hz, 1H), 3.59 (d, *J* = 7.1 Hz, 2H), 3.67 (s, 3H), 4.20 (ddd, *J* = 11.7 Hz, 7.1 Hz, 4.8 Hz, 1H), 4.58 (quintet, *J* = 7.4 Hz, 1H), 4.72 (quintet, *J* = 7.2 Hz, 1H), 4.76 (t, *J* = 7.1 Hz, 1H), 5.29 (dd, *J* = 7.3 Hz and 4.3 Hz, 1H), 7.27 (br d, *J* = 7.7 Hz, 1H), 7.41 (d, *J* = 8.5 Hz, 2H), 7.56 (br d, *J* = 7.6 Hz, 1H), 7.75 (br d, *J* = 7.0 Hz, 1H), 7.79 (d, *J* = 8.6 Hz, 2H); ^13^C-NMR δ 17.4, 17.8, 31.7, 36.9, 48.2, 49.4, 52.4, 54.3, 58.0, 64.9, 128.78, 128.85, 131.8, 138.3, 166.7, 168.5, 171.9, 173.3, 173.6; HRMS (TOF ES^+^) *m*/*z* calculated for C_21_H_26_ClN_4_O_6_S (M + H) 497.1256; found 497.1253.

**(2*S*)-Methyl2-((3*R*)-6-((*S*)-2-(4-chlorobenzamido)propanamido)-6-methyl-5-oxohexahydropyrrolo-[2,1-b]thiazole-3-carboxamido)propanoate (α-36b and β-36b):** Separated by reverse-phase HPLC on a Dynamax Microsorb 5-micron C18 column (21.4 × 250 mm) using a step gradient beginning with 60/40 of 1:1 MeCN/MeOH with 5.0 mM ammonium acetate/water with 5.0 mM ammonium acetate to afford 4.2 mg (4% over 13 steps) of **α-36b**; ^1^H-NMR δ 1.38 (d, *J* = 7.1 Hz, 3H), 1.49 (d, *J* = 7.0 Hz, 3H), 1.59 (s, 3H), 2.63 (dd, *J* = 13.0 Hz and 7.4 Hz, 1H), 2.79 (dd, *J* = 13.0 Hz and 6.6 Hz, 1H), 3.47 (dd, *J* = 11.9 Hz and 7.5 Hz, 1H), 3.69 (dd, *J* = 11.9 Hz and 5.9 Hz, 1H), 3.75 (s, 3H), 4.50 (quintet, *J* = 7.1 Hz, 1H), 4.78 (quintet, *J* = 7.0 Hz, 1H), 4.94 (dd, *J* = 7.0 and 6.2 Hz, 1H), 5.11 (t, *J* = 7.0 Hz, 1H), 6.97 (br d, *J* = 7.2 Hz, 1H), 7.02 (br s, 1H), 7.35 (br d, *J* = 6.9 Hz, 1H), 7.41 (d, *J* = 8.5 Hz, 2H), 7.74 (d, *J* = 8.5 Hz, 2H); ^13^C-NMR δ 18.0, 18.9, 23.6, 35.2, 41.7, 48.4, 49.1, 52.6, 58.8, 61.7, 62.5, 128.5, 128.9, 131.9, 138.2, 166.0, 168.1, 171.7, 172.9, 174.5; HRMS (TOF ES^+^) *m*/*z* calculated for C_22_H_28_ClN_4_O_6_S (M + H) 511.1413; found 511.1416 and 4.1 mg (4% over 13 steps) of **β-36b**; ^1^H-NMR δ 1.42 (d, *J* = 6.9 Hz, 3H), 1.45 (d, *J* = 7.2 Hz, 3H), 1.50 (s, 3H), 2.26 (dd, *J* = 14.2 Hz and 4.3 Hz, 1H), 2.78 (dd, *J* = 14.3 Hz and 7.8 Hz, 1H), 3.55–3.62 (m, 2H), 3.64 (s, 3H), 4.63 (quintet, *J* = 7.5 Hz, 1H), 4.77 (quintet, *J* = 7.2 Hz, 1H), 4.81 (dd, *J* = 8.1 Hz and 5.8 Hz, 1H), ), 5.26 (dd, *J* = 7.8 Hz and 4.4 Hz, 1H), 7.21 (br s, 1H), 7.26 (br d, *J* = 6.8 Hz, 1H), 7.38 (d, *J* = 8.4 Hz, 2H), 7.66 (br d, *J* = 7.9 Hz, 1H), 7.77 (d, *J* = 8.5 Hz, 2H); ^13^C-NMR δ 16.9, 18.2, 25.6, 36.6, 38.8, 47.9, 48.8, 52.3, 57.5, 60.7, 62.9, 128.7, 128.8, 132.1, 138.2, 167.1, 168.4, 172.2, 172.8, 173.3; HRMS (TOF ES^+^) *m*/*z* calculated for C_22_H_28_ClN_4_O_6_S (M + H) 511.1413; found 511.1411.

**(2*S*)-Methyl2-((3*R*)-6-benzyl-6-((*S*)-2-(4-chlorobenzamido)propanamido)-5-oxohexahydropyrrolo-[2,1-b]thiazole-3-carboxamido)propanoate (α-36c and β-36c):** Compound **α-36c** was separated from **β-36c** by chromatography on 1.5 g of silica gel. It was further purified by reverse-phase HPLC on a Dynamax Microsorb 5-micron C18 column (21.4 × 250 mm) using a step gradient beginning with 70/30 of 1:1 MeCN/MeOH with 5.0 mM of ammonium acetate/water with 5.0 mM of ammonium acetate to afford 2.1 mg (1% over 13 steps) of **α-36c**; ^1^H-NMR δ 1.37 (d, *J* = 6.9 Hz, 3H), 1.44 (d, *J* = 7.2 Hz, 3H), 2.54 (d, *J* = 6.2 Hz, 2H), 3.02 (d, *J* = 13.6 Hz, 1H), 3.10 (d, *J* = 13.6 Hz, 1H), 3.42 (dd, *J* = 11.6 Hz and 8.6 Hz, 1H), 3.50 (dd, *J* = 11.6 Hz and 5.2 Hz, 1H), 3.67 (s, 3H), 4.63 (quintet, *J* = 7.6 Hz, 1H), 4.72 (quintet, *J* = 7.4 Hz, 1H), 4.78 (dd, *J* = 8.5 Hz and 5.2 Hz, 1H), 5.23 (t, *J* = 6.2 Hz, 1H), 7.11 (br d, *J* = 8.4 Hz, 1H), 7.12 (br s, 1H), 7.24–7.31 (m, 5H), 7.42 (d, *J* = 8.5 Hz, 2H), 7.63 (br d, *J* = 8.0 Hz, 1H), 7.79 (d, *J* = 8.5 Hz, 2H); ^13^C-NMR δ 16.5, 18.2, 36.0, 36.4, 42.9, 47.9, 48.8, 52.3, 57.5, 63.1, 64.6, 127.8, 128.7, 128.86, 128.93, 130.3, 132.0, 133.9, 138.2, 167.1, 168.2, 171.2, 172.6, 173.3; HRMS (TOF ES^+^) *m*/*z* calculated for C_28_H_31_ClN_4_O_6_SNa (M + Na) 609.1551; found 609.1565. Compound **β-36c** was further purified by reverse-phase HPLC on a Dynamax Microsorb 5-micron C18 column (21.4 × 250 mm) using a step gradient beginning with 70/30 of 1:1 MeCN/MeOH with 5.0 mM of ammonium acetate/water with 5.0 mM of ammonium acetate to afford 3.2 mg (2% over 13 steps) of **β-36c**; ^1^HNMR δ 1.39 (d, *J* = 7.3 Hz, 3H), 1.50 (d, *J* = 6.9 Hz, 3H), 2.57 (dd, *J* = 13.5 Hz and 7.0 Hz, 1H), 3.02 (dd, *J* = 13.5 Hz and 6.8 Hz, 1H), 3.09 (d, *J* = 13.0 Hz, 1H), 3.24 (d, *J* = 13.0 Hz, 1H), 3.32 (dd, *J* = 11.6 Hz and 6.9 Hz, 1H), 3.61 (dd, *J* = 11.7 Hz and 5.5 Hz, 1H), 3.79 (s, 3H), 4.09 (t, *J* = 6.9 Hz, 1H), 4.57 (quintet, *J* = 7.3 Hz, 1H), 4.69 (quintet, *J* = 7.1 Hz, 1H), 4.85 (t, *J* = 6.2 Hz, 1H), 6.70 (br d, *J* = 7.2 Hz, 1H), 6.84 (br s, 1H), 6.88 (br d, *J* = 7.6 Hz, 1H), 7.21–7.22 (m, 2H), 7.27–7.28 (m, 3H), 7.42 (d, *J* = 8.5 Hz, 2H), 7.73 (d, *J* = 8.5 Hz, 2H); ^13^C-NMR δ 18.2, 18.3, 34.4, 40.6, 42.4, 48.2, 49.2, 52.6, 60.1, 62.1, 65.7, 128.0, 128.5, 128.7, 128.9, 130.1, 131.9, 133.9, 138.3, 166.1, 167.4, 171.5, 172.7, 174.3; HRMS (TOF ES^+^) *m*/*z* calculated for C_28_H_31_ClN_4_O_6_SNa (M + Na) 609.1551; found 609.1572.

**(2*S*)-Methyl2-((3*R*)-6-benzyl-6-((*S*)-2-(4-nitrobenzamido)propanamido)-5-oxohexahydropyrrolo[2,1-b]thiazole-3-carboxamido)propanoate (β-30c and α-30c):** Partial separation was achieved by chromatography on 1.0 g of silica gel using CH_2_Cl_2_ and CH_2_Cl_2_/EtOAc mobile phases. Compound **β-30c** was then purified by reverse-phase HPLC on a Dynamax Microsorb 5-micron, C18 column (21.4 × 250 mm) using 65/35 of 1:1 MeCN/MeOH with 5.0 mM of ammonium acetate/water with 5.0 mM of ammonium acetate to afford 9.1 mg (4% over 13 steps) of **β-30c**; ^1^H-NMR δ 1.40 (d, *J* = 7.2 Hz, 3H), 1.54 (d, *J* = 7.0 Hz, 3H), 2.59 (dd, *J* = 13.4 Hz and 7.0 Hz, 1H), 3.06 (dd, *J* = 13.5 Hz and 6.8 Hz, 1H), 3.11 (d, *J* = 13.1 Hz, 1H), 3.25 (d, *J* = 13.1 Hz, 1H), 3.32 (dd, *J* = 11.7 Hz and 7.0 Hz, 1H), 3.60 (dd, *J* = 11.7 Hz and 5.6 Hz, 1H), 3.79 (s, 3H), 4.14 (t, *J* = 6.9 Hz, 1H), 4.57 (quintet, *J* = 7.3 Hz, 1H), 4.75 (quintet, *J* = 7.0 Hz, 1H), 4.85 (t, *J* = 6.3 Hz, 1H), 6.84 (br s, 1H), 6.92 (br d, *J* = 7.6 Hz, 1H), 7.07 (br d, *J* = 7.1 Hz, 1H), 7.21–7.23 (m, 2H), 7.26–7.29 (m, 3H), 7.97 (d, *J* = 8.6 Hz, 2H), 8.29 (d, *J* = 8.7 Hz, 2H); ^13^C-NMR δ 18.2, 18.7, 34.6, 40.7, 42.4, 48.3, 49.4, 52.6, 60.0, 62.2, 65.8, 123.8, 128.0, 128.4, 128.8, 130.1, 133.8, 139.1, 149.8, 165.0, 167.4, 171.5, 172.7, 174.1; HRMS (TOF ES^+^) *m*/*z* calculated for C_28_H_32_N_5_O_8_SNa (M + H) 598.1966; found 598.1957. Compound **α-30c** was purified by reverse-phase HPLC on a Dynamax Microsorb 5-micron, C18 column (21.4 × 250 mm) using 65/35 of 1:1 MeCN/MeOH with 5.0 mM of ammonium acetate/water with 5.0 mM ammonium acetate to afford 4.7 mg (2% over 13 steps) of **α-30c**; ^1^H-NMR δ 1.39 (d, *J* = 6.9 Hz, 3H), 1.45 (d, *J* = 7.2 Hz, 3H), 2.56 (d, *J* = 6.2 Hz, 2H), 3.03 (d, *J* = 13.7 Hz, 1H), 3.12 (d, *J* = 13.7 Hz, 1H), 3.44 (dd, *J* = 11.6 Hz and 8.4 Hz, 1H), 3.48 (dd, *J* = 11.7 Hz and 5.4 Hz, 1H), 3.65 (s, 3H), 4.67 (quintet, *J* = 7.2 Hz, 1H), 4.74–4.77 (m, 2H), 5.25 (t, *J* = 6.1 Hz, 1H), 7.09 (br s, 1H), 7.25–7.26 (m, 2H), 7.29–7.34 (m, 3H), 7.43 (br d, *J* = 7.9 Hz, 1H), 7.62 (br d, *J* = 8.2 Hz, 1H), 8.04 (d, *J* = 8.7 Hz, 2H), 8.29 (d, *J* = 8.8 Hz, 2H); ^13^C-NMR δ 16.3, 18.4, 35.7, 36.6, 42.9, 47.8, 49.0, 52.4, 57.4, 63.2, 64.8, 123.7, 127.9, 128.6, 129.0, 130.3, 133.9, 139.4, 149.8, 166.3, 168.1, 171.1, 172.5, 173.6; HRMS (TOF ES^+^) *m*/*z* calculated for C_28_H_32_N_5_O_8_SNa (M + H) 598.1966; found 598.1960.

**(2*S*)-Methyl2-((3*R*)-6-((*S*)-2-((((9H-fluoren-9-yl)methoxy)carbonyl)amino)-propanamido)-6-methyl-5-oxohexahydropyrrolo[2,1-b]thiazole-3-carboxamido)-propanoate (α-37b and β-37b):** To 252 μmol of resin **13b** (R^1^ = Me) swelled with NMP contained in a 3.5-mL reaction vessel, 2 mL of 20% piperidine in NMP was added. The vessel was drained, charged with 3 mL of the 20% piperidine solution, and rotated at room temperature for 45 minutes. The vessel was drained, and the resin was washed with 7 × 3 mL of NMP to remove all of the piperidine. The resin was then treated with a solution of 1.25 mmol (5 equiv.) each of HBTU and Fmoc-Ala-OH and 2.50 mmol (10 equiv.) of DIEA in 3.75 mL of NMP. The vessels were rotated at room temperature for six days, drained, and the resins were washed with 3 × 3 mL each of NMP, 1;1 THF:MeOH, THF, and 4 × 3 mL of dichloromethane to give acetal resin **22b** (R^1^ = Me), which was hydrolyzed using 3 mL of 4:4:1 TFA:CH_2_Cl_2_/water over 35 minutes at room temperature. The vessel was drained, and the resulting aldehyde resin was washed six times with dichloromethane. To this resin, a solution of 32 in 1 mL of acetic acid that was generated from 268 μmol (1.07 equiv.) of 31 was then added, followed by a solution of 2.0 mmol (8.0 equiv.) of potassium acetate in acetic acid. After exposure at room temperature for 18 h, the vessel was drained into a tared collection vial. The resin was washed with 2 × 2 mL of acetic acid, and the filtrates were combined. The resin was treated with 3 mL of acetic acid, and was placed in an oven heated at 45–52 °C for 66 h. The vessels were drained, and the resin was washed with 2 × 2 mL of acetic acid. This filtrate was evaporated to give 25.3 mg of **α-37b** and **β-37b**. The diastereomers were separated by reverse-phase HPLC on a Dynamax Microsorb 5-micron, C18 column (21.4 × 250 mm) using a step gradient beginning with 75/25 of 1:1 MeCN/MeOH with 5.0 mM of ammonium acetate/water with 5.0 mM of ammonium acetate to afford 3.7 mg (3% over 11 steps) of **α-37b**; ^1^H-NMR δ 1.35 (d, *J* = 6.6 Hz, 3H), 1.45 (d, *J* = 7.2 Hz, 3H), 1.49 (s, 3H), 2.24 (br dd, *J* = 14.0 Hz and 3.2 Hz, 1H), 2.75 (br dd, *J* = 13.1 Hz and 7.4 Hz, 1H), 3.55 (dd, *J* = 11.7 Hz and 8.7 Hz, 1H), 3.62 (dd, *J* = 11.7 Hz and 5.0 Hz, 1H), 3.69 (s, 3H), 4.21 (t, *J* = 7.0 Hz, 1H), 4.26 (br m, 1H), 4.39–4.47 (br m, 2H), 4.56 (quintet, *J* = 7.3 Hz, 1H), 4.86 (dd, *J* = 8.6 Hz and 5.1 Hz, 1H), 5.21 (br m, 1H), 5.58 (br d, *J* = 6.0 Hz, 1H), 6.75 (br s, 1H), 7.30 (q, *J* = 7.6 Hz, 2H), 7.40 (t, *J* = 7.4 Hz, 2H), 7.58 (d, *J* = 7.5 Hz, 2H), 7.59 (br d, *J* = 6.0 Hz, 1H), 7.76 (d, *J* = 7.6 Hz, 2H); ^13^C-NMR δ 17.8, 25.7, 36.3, 38.9, 47.1, 48.3, 50.4, 52.4, 57.7, 60.3, 62.6, 67.2, 120.0, 124.96, 125.04, 127.06, 127.12, 127.8, 141.3, 143.6, 143.7, 156.5, 168.5, 172.5, 172.6, 172.8; HRMS (TOF ES^+^) *m*/*z* calculated for C_30_H_35_N_4_O_7_S (M + H) 595.2221; found 595.2219 and 5.5 mg (4% over 11 steps) of **β-37b**: ^1^H-NMR δ 1.39 (d, *J* = 7.2 Hz, 6H), 1.56 (s, 3H), 2.62 (br dd, *J* = 12.6 Hz and 7.3 Hz, 1H), 2.78 (br dd, *J* = 12.8 Hz and 6.5 Hz, 1H), 3.42 (dd, *J* = 11.7 Hz and 7.3 Hz, 1H), 3.71 (dd, *J* = 11.8 Hz and 6.1 Hz, 1H), 3.75 (s, 3H), 4.22 (quintet, *J* = 7.1 Hz, 1H), 4.25 (br m, 1H), 4.40 (br d, *J* = 6.8 Hz, 2H), 4.51 (quintet, *J* = 7.1 Hz, 1H), 4.84 (t, *J* = 6.6 Hz, 1H), 5.06 (t, *J* = 7.1 Hz, 1H), 5.33 (br d, *J* = 6.5 Hz, 1H), 6.61 (br s, 1H), 7.19 (br d, *J* = 6.2 Hz, 1H), 7.32 (t, *J* = 7.6 Hz, 2H), 7.41 (t, *J* = 7.0 Hz, 2H), 7.58 (d, *J* = 7.4 Hz, 2H), 7.76 (d, *J* = 7.6 Hz, 2H); ^13^C-NMR δ 18.1, 23.5, 34.9, 41.6, 47.1, 48.4, 50.3, 52.6, 59.1, 61.5, 62.4, 67.2, 120.0, 125.03, 125.06, 127.1, 127.8, 141.3, 143.7, 156.0, 167.8, 171.6, 172.9, 174.9; HRMS (TOF ES^+^) *m*/*z* calculated for C_30_H_34_N_4_O_7_SNa (M + Na) 617.2046; found 617.2043.

## 4. Summary and Conclusions

### 4.1. Synthesis and Use of Key Intermediate ***13***

The synthesis of Fmoc acetal resin **13** from the advanced intermediate amino resin **9** is described (Scheme 3). Since ozonolysis of the allyl group precedes introduction of the R^2^CO group, the strategy enables the incorporation of ozone-labile moieties such as furan and trimethoxyphenyl at R^2^ for a variety of peptidomimetic and biomimetic scaffolds **1**–**5**. Resin **13** also features two orthogonally-related Fmoc and acetal protecting groups, which can be selectively removed under basic and acidic conditions, respectively, thereby providing an Fmoc-based, SPPS approach to unnatural peptides and peptidomimetics **1**–**5**. The versatility of Fmoc acetal resin **13** is illustrated with the syntheses of homoserine lactones **19a**–**c** (scaffold **1**) and the bicyclic thiazolidine **21c** (scaffold **3**), which are examples that contain ozone-labile substructures. Homoserine lactone **24c** and many of the bicyclic thiazolidine compounds listed in Table 2 offer examples of scaffolds **1** and **3** containing amino acid residues. Most of the bicyclic thiazolidines **3** were synthesized in 10–13 steps from commercially available Boc-protected glycine, alanine, and phenylalanine on Merrifield resin. However, access to the multiple scaffolds from the key orthogonally-protected intermediate **13** was often accomplished in four or fewer steps. Several examples (**36a**–**c**, **30b**–**c**, and **37b**) represent the successful application of peptide fragment condensation in which Cys-Ala-OMe is condensed with the Fmoc-Ala extended analog of **13a**–**c**.

### 4.2. NMR Characterization: Stereochemistry and Possible Secondary Structure

The cyclitive cleavage process in acetic acid at elevated temperatures using cysteine-based nucleophiles to generate scaffold **3** compounds proceeds to afford primarily two diastereomers in ratios from 1:1 to 4:1. These stereoisomers were separated by normal-phase or reverse-phase chromatography, and their relative configurations were determined by one and two-dimensional nOe studies. The difference in chemical shifts (∆δ) between diastereotopic protons at C-6 (the methylene group of the lactam ring) is seen to be diagnostic. For the R^1^ = Bn and H series, ∆δ is significantly larger for the β isomer (major diastereoisomer), whereas for the R^1^ = Me series, it is the α isomer that displays the larger values. Thus, knowledge of the C-6 proton ∆δ values of the two major diastereomers in the three series (R^1^ = H, Me, Bn) affords a predictive value in the assignment of the stereochemistry of future compounds. Nuclear Overhauser enhancement studies also reveal a small enhancement of signals from remote protons located on opposite ends of the four-residue peptidomimetic sequence of **α**-**30b**, and may suggest that the molecule adopts a β-turn secondary structure in chloroform.

## Figures and Tables

**Table 1:** Crystal data and structure refinement for **β**-**26c** (Reciprocal Net report 08134).

Empirical formula	C_25_H_28_N_3_O_5.50_S	
Formula weight	490.56	
Crystal color, shape, size	colorless block, 0.25 × 0.22 × 0.17 mm^3^	
Temperature	150(2) K	
Wavelength	0.71073 Å	
Crystal system, space group	Monoclinic, P2(1)	
Unit cell dimensions	a = 9.222 Å	α = 90°
	b = 27.417 Å	β = 91.73°
	c = 9.552 Å	γ = 90°
Volume	2414.0 Å^3^	
Z	4	
Density (calculated)	1.350 Mg/m^3^	
Absorption coefficient	0.178 mm^−1^	
F(000)	1036	
**Data collection**		
Diffractometer	APEX II Kappa Duo, Bruker	
Theta range for data collection	1.49 to 28.39°	
Index ranges	−12 ≤ h ≤ 11, −36 ≤ k ≤ 27, −12 ≤ l ≤ 12	
Reflections collected	22656	
Independent reflections	10036 [R(int) = 0.0428]	
Observed Reflections	8608	
Completeness to theta = 28.39°	99.5%	
**Solution and Refinement**		
Absorption correction	Semi-empirical from equivalents	
Max. and min. transmission	0.9704 and 0.9568	
Solution	Direct methods	
Refinement method	Full-matrix least-squares on F^2^	
Weighting scheme	w = [σ^2^F_o_^2^ + AP^2^ + BP]^−1^, with P = (F_o_^2^ + 2 F_c_^2^)/3, A =, B =	
Data/restraints/parameters	10036/259/638	
Goodness of fit on F^2^	1.044	
Final R indices [I > 2sigma(I)]	R1 = 0.0404, wR2 = 0.0889	
R indices (all data)	R1 = 0.0522, wR2 = 0.0946	
Absolute structure parameter	0.04 (5)	
Largest diff. peak and hole	0.319 and −0.320 e.Å^−3^	

Goodness-of-fit = [Σ[w(F_o_^2^ − F_c_^2^)^2^]/N_observns_ − N_params_)]^1/2^, all data. R1 = Σ(|F_o_| − |F_c_|)/Σ |F_o_|. wR2 = [Σ[w(F_o_^2^ − F_c_^2^)^2^]/Σ [w(F_o_^2^)^2^]]^1/2^.

**Table 2:**
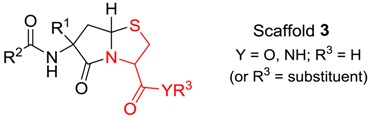
Bicyclic Lactams **3**.

Entry	Cmpd	R^1^	R^2^	YR^3^	Cleavage Method	dr (β/α) ^a^	%Overall Yield (Steps)	Δδ (ppm)	R^1^
**1**	**β-27c**	benzyl	4-MePh	OEt	A	80:20	29 (10)	0.69	β
**2**	**α-27c**	benzyl	4-MePh	OEt	A			0.24	α
**3**	**α-33a**	H	4-ClPh	OEt	C	49:51	8 (11)	0.37	α
**4**	**β-33a**	H	4-ClPh	OEt	C		8 (11)	1.24	β
**5**	**β-33b**	Me	4-ClPh	OEt	B	87:13	5 (11)	0.36	β
**6**	**α-33b**	Me	4-ClPh	OEt	E		4 (11)	0.59	α
**7**	**β-33c**	benzyl	4-ClPh	OEt	F	87:13	13 (11)	0.71	β
**8**	**α-33c**	benzyl	4-ClPh	OEt	F		1 (11)	0.23	α
**9**	**β-26c**	benzyl	4-MePh	NHCH_2_CO_2_H	A	70:30	7 (10)	0.26 ^b^	β
**10**	**β-34a**	H	4-FPh	NH-Leu-OMe	C	41:59	17 (5) ^c^	1.21	β
**11**	**α-34a**	H	4-FPh	NH-Leu-OMe	C		15 (5)	0.09	α
**12**	**α-35a**	H	4-ClPh	NH-Ala-OMe	D	55:45	6 (11)	0.09	α
**13**	**β-35a**	H	4-ClPh	NH-Ala-OMe	D		4 (11)	1.21	β
**14**	**β-35b**	Me	4-ClPh	NH-Ala-OMe	D	52:48	9 (11)	0.26	β
**15**	**α-35b**	Me	4-ClPh	NH-Ala-OMe	D		9 (11)	0.54	α
**16**	**β-35c**	benzyl	4-ClPh	NH-Ala-OMe	D	66:34	8 (11)	0.58	β
**17**	**α-35c**	benzyl	4-ClPh	NH-Ala-OMe	D		3 (11)	0.06	α
**18**	**β-36a**	H	4-ClPh-Ala	NH-Ala-OMe	D	60:40	4 (13) ^d^	1.07	β
**19**	**α-36a**	H	4-ClPh-Ala	NH-Ala-OMe	D		3 (13)	0.08	α
**20**	**β-36b**	Me	4-ClPh-Ala	NH-Ala-OMe	D	59:41	4 (13)	0.16	β
**21**	**α-36b**	Me	4-ClPh-Ala	NH-Ala-OMe	D		4 (13)	0.52	α
**22**	**α-36c**	benzyl	4-ClPh-Ala	NH-Ala-OMe	D	58:42	2 (13)	0.00	α
**23**	**β-36c**	benzyl	4-ClPh-Ala	NH-Ala-OMe	D		1 (13)	0.45	β
**24**	**α-30b**	Me	4-NO_2_Ph-Ala	NH-Ala-OMe	D	58:42	9 (13)	0.52	α
**25**	**β-30b**	Me	4-NO_2_Ph-Ala	NH-Ala-OMe	D		7 (13)	0.17	β
**26**	**β-30c**	benzyl	4-NO_2_Ph-Ala	NH-Ala-OMe	D	66:34	4 (13)	0.47	β
**27**	**α-30c**	benzyl	4-NO_2_Ph-Ala	NH-Ala-OMe	D		2 (13)	0.00	α
**28**	**α-37b**	Me	Fmoc-Ala	NH-Ala-OMe	D	62:38	3 (11)	0.51	α
**29**	**β-37b**	Me	Fmoc-Ala	NH-Ala-OMe	D		4 (11)	0.16	β

^a^ Material released from resin was collected after 24 h at rt, then after successive 24 h/55 °C periods. The diastereomeric ratio (dr) cited is that from the most productive, the first 24 h/55 °C period; β is defined as R^1^ “up” (wedge bond), ^b^ in methanol-d_4_, ^c^ number of steps from Boc-Gly(allyl)-Merrifield, ^d^ an additional 3% was obtained as a mixture of the two diastereomers. Method A: Acetal resin **25c**, 2.0–2.5 equiv. Cys(OEt)·HCl or Cys-Gly, 5–7 equiv.KOAc, acetic acid, 90 °C/24 h. Method B: Aldehyde resin **7** (R^1^ = Me, R^2^ = 4-ClC_6_H_5_), 2.0 equiv. Cys(OEt)·HCl, 6 equiv KOAc, AcOH, rt/24h, filter then replace with PhCl/6 equiv DIEA/55–80 °C/88 h. Method C: Aldehyde resin **7** (R^1^ = H, R^2^ = 4-ClC_6_H_5_ or 4-FC_6_H_5_), 0.8–0.9 equiv. Cys(OEt)·HCl or Cys-AA-OMe·HCl or TFA, 1.4–1.7 equiv poly-4-vinylpyridine (PVP), AcOH/rt-55 °C/66 h. Method D: Aldehyde resin **7**, 0.9–1.3 equiv. Cys(OEt)·HCl or Cys-AA-OMe·TFA, 6 equiv KOAc, AcOH, rt then 55 °C. Method E: Conditions of Method B followed by methoxide cleavage (22 equiv sodium methoxide in methanol/THF/3 h) to give the acid which was then esterified (DBU, EtI/THF). Method F: Aldehyde resin **7** (R^1^ = Me, R^2^ = 4-ClC_6_H_5_), 2.0 equiv. Cys(OEt)·HCl, 6 equiv KOAc, AcOH, rt/24 h, filter then replace with PhCl/6 equiv DIEA/55 °C/24 h, filter then replace with AcOH/75–90 °C/66 h.

**Table 3:**
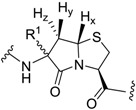
Chemical shifts of H_x_, H_y_, and H_z_ and the chemical shift differences between H_y_ and H_z_ in diastereomeric pairs.

	R^1^	δ (H_x_)	δ (H_y_)	δ (H_z_)	Δδ_yz_	Isomer
α-33a β-33a	H	5.24 5.21	2.49 3.35	2.86 2.11	0.37 1.24	α β
α-34a β-34a	H	5.31 5.11	2.56 3.35	2.65 2.14	0.09 1.21	α β
α-35a β-35a	H	5.32 5.13	2.56 3.35	2.65 2.14	0.09 1.21	α β
α36a β-36a	H	5.29 5.06	2.48 3.14	2.56 2.07	0.08 1.07	α β
α-33b β-33b	Me	5.34 5.21	3.10 3.03	2.51 2.67	0.59 0.36	α β
α-35b β-35b	Me	5.26 5.13	2.88 2.99	2.34 2.73	0.54 0.26	α β
α-36b β-36b	Me	5.26 5.11	2.79 2.79	2.26 2.63	0.52 0.16	α β
α-30b β-30b	Me	5.26 5.15	2.80 2.82	2.28 2.65	0.52 0.17	α β
α-37b β-37b	Me	5.21 5.06	2.75 2.78	2.24 2.62	0.51 0.16	α β
α-33c β-33c	Bn	5.39 4.75	2.93 3.32	2.71 2.61	0.22 0.71	α β
α-35c β-35c	Bn	5.25 4.25	2.62 3.27	2.68 2.69	0.06 0.58	α β
α-36c β-36c	Bn	5.23 4.09	2.54 3.02	2.54 2.58	0.00 0.44	α β
α-30c β-30c	Bn	5.25 4.14	2.56 3.06	2.56 2.59	0.00 0.47	α β

## Supplementary Materials

The following are available online at http://www.mdpi.com/1420-3049/23/7/1762/s1, **Figure S1.** Proton NMR Spectrum of **19a** in CDCl_3_, **Figure S2.** Carbon-13 NMR Spectrum of **19a** in CDCl_3_, **Figure S3.** Proton NMR Spectrum of **19b** in CDCl_3_, **Figure S4.** Carbon-13 NMR Spectrum of **19b** in CDCl_3_, **Figure S5.** Proton NMR Spectrum of **19c** in CDCl_3_, **Figure S6.** Carbon-13 NMR Spectrum of **19c** in CDCl_3_, **Figure S7.** Proton NMR Spectrum of **α**-**21c** and **β**-**21c** in CDCl_3_, **Figure S8.** Carbon-13 NMR Spectrum of **α**-**21c** and **β**-**21c** in CDCl_3_, **Figure S9.** Proton NMR Spectrum of **24c** (earlier R_t_) in CD_3_OD, **Figure S10.** Carbon-13 NMR Spectrum of **24c** (earlier R_t_) in CD_3_OD, **Figure S11.** Proton NMR Spectrum of **24c** (later R_t_) in CDCl_3_, **Figure S12.** Carbon-13 NMR Spectrum of **24c** (later R_t_) in CDCl_3_, **Figure S13.** Proton NMR Spectrum of **β**-**26c** in CD_3_OD, **Figure S14.** Carbon-13 NMR Spectrum of **β**-**26c** in CD_3_OD, **Figure S15.** Proton NMR Spectrum of **31** in CDCl_3_, **Figure S16.** Carbon-13 NMR Spectrum of **31** in CDCl_3_, **Figure S17.** Proton NMR Spectrum of **α**-**33a** in CDCl_3_, **Figure S18.** Carbon-13 NMR Spectrum of **α**-**33a** in CDCl_3_, **Figure S19.** Proton NMR Spectrum of **β**-**33a** in CDCl_3_, **Figure S20.** Carbon-13 NMR Spectrum of **β**-**33a** in CDCl_3_, **Figure S21.** Proton NMR Spectrum of **β**-**33b** in CDCl_3_, **Figure S22.** Carbon-13 NMR Spectrum of **β**-**33b** in CDCl_3_, **Figure S23.** Proton NMR Spectrum of **α**-**33b** in CDCl_3_, **Figure S24.** Carbon-13 NMR Spectrum of **α**-**33b** in CDCl_3_, **Figure S25.** Proton NMR Spectrum of **β**-**33c** in CDCl_3_, **Figure S26.** Carbon-13 NMR Spectrum of **β**-**33c** in CDCl_3_, **Figure S27.** Proton NMR Spectrum of **α**-**33c** in CDCl_3_, **Figure S28.** Carbon-13 NMR Spectrum of **α**-**33c** in CDCl_3_, **Figure S29.** Proton NMR Spectrum of **β**-**34a** in CDCl_3_, **Figure S30.** Carbon-13 NMR Spectrum of **β**-**34a** in CDCl_3_, **Figure S31.** Proton NMR Spectrum of **α**-**34a** in CDCl_3_, **Figure S32**. Carbon-13 NMR Spectrum of **α**-**34a** in CDCl_3_, **Figure S33.** Proton NMR Spectrum of **α**-**35a** in CDCl_3_, **Figure S34.** Carbon-13 NMR Spectrum of **α**-**35a** in CDCl_3_, **Figure S35.** Proton NMR Spectrum of **β**-**35a** in CDCl_3_, **Figure S36.** Carbon-13 NMR Spectrum of **β**-**35a** in CDCl_3_, **Figure S37.** Proton NMR Spectrum of **β**-**35b** in CDCl_3_, **Figure S38.** Carbon-13 NMR Spectrum of **β**-**35b** in CDCl_3_, **Figure S39.** Proton NMR Spectrum of **α**-**35b** in CDCl3, **Figure S40.** Carbon-13 NMR Spectrum of **α**-**35b** in CDCl_3_, **Figure S41.** Proton NMR Spectrum of **β**-**35c** in CDCl_3_, **Figure S42.** Carbon-13 NMR Spectrum of **β**-**35c** in CDCl_3_, **Figure S43.** Proton NMR Spectrum of **α**-**35c** in CDCl_3_, **Figure S44.** Carbon-13 NMR Spectrum of **α**-**35c** in CDCl_3_, **Figure S45.** Proton NMR Spectrum of **β**-**36a** in CDCl_3_, **Figure S46.** Carbon-13 Spectrum of **β**-**36a** in CDCl_3_, **Figure S47.** Proton NMR Spectrum of **α**-**36a** in CDCl_3_, **Figure S48.** Carbon-13 NMR Spectrum of **α**-**36a** in CDCl_3_, **Figure S49.** Proton NMR Spectrum of **β**-**36b** in CDCl_3_, **Figure S50.** Carbon-13 NMR Spectrum of **β**-**36b** in CDCl_3_, **Figure S51.** Proton NMR Spectrum of **α**-**36b** in CDCl_3_, **Figure S52.** Carbon-13 NMR Spectrum of **α**-**36b** in CDCl_3_, **Figure S53.** Proton NMR Spectrum of **α**-**36c** in CDCl_3_, **Figure S54.** Carbon-13 NMR Spectrum of **α**-**36c** in CDCl_3_, **Figure S55.** Proton NMR Spectrum of **β**-**36c** in CDCl_3_, **Figure S56.** Carbon-13 NMR Spectrum of **β**-**36c** in CDCl_3_, **Figure S57.** Proton NMR Spectrum of **α**-**30b** in CDCl_3_, **Figure S58.** Carbon-13 NMR Spectrum of **α**-**30b** in CDCl_3_, **Figure S59.** Proton NMR Spectrum of **β**-**30b** in CDCl_3_, **Figure S60.** Carbon-13 NMR Spectrum of **β**-**30b** in CDCl_3_, **Figure S61.** Proton NMR Spectrum of **β**-**30c** in CDCl_3_, **Figure S62.** Carbon-13 NMR Spectrum of **β**-**30c** in CDCl_3_, **Figure S63.** Proton NMR Spectrum of **α**-**30c** in CDCl_3_, **Figure S64.** Carbon-13 NMR Spectrum of **α**-**30c** in CDCl_3_, **Figure S65.** Proton NMR Spectrum of **α**-**37b** in CDCl_3_, **Figure S66.** Carbon-13 NMR Spectrum of **α**-**37b** in CDCl_3_, **Figure S67.** Proton NMR Spectrum of **β**-**37b** in CDCl_3_, **Figure S68.** Carbon-13 NMR Spectrum of **β**-**37b** in CDCl_3_. **Figure S69.** Proton NMR Spectrum of (Fmoc-Ala)_2_O in CDCl_3_ with Diisopropylurea (DIU), **Figure S70**. Proton NMR Spectrum of Boc-Cys(Trt)-Leu-OMe in CDCl_3_, **Figure S71.** Carbon-13 NMR Spectrum of Boc-Cys(Trt)-Leu-OMe in CDCl_3_.
