# Supplementary material for: Preparation and Use of a General Solid-Phase Intermediate to Biomimetic Scaffolds and Peptide Condensations
Source: Molecules. 2018 Jul 18;23(7):1762. doi: 10.3390/molecules23071762 (PMC6100553; doi:10.3390/molecules23071762)
Supplement: Supplementary file 1 [file molecules-23-01762-s001.pdf]

Supplementary Materials

Preparation and Use of a General Solid-Phase Intermediate to Biomimetic Scaffolds and Peptide Condensations

J. Geno Samaritoni [1,*], Jacek G. Martynow [2], Martin J. O'Donnell [1] and William L. Scott [1]

[1] Indiana University Purdue University Indianapolis, Department of Chemistry and Chemical Biology, 402 N. Blackford St., Indianapolis 46202, Indiana; modonnel@iupui.edu (M.J.O.); wscott@iupui.edu (W.L.S.)
[2] Melinta Therapeutics, Inc., 300 George Street, S 301, New Haven, CT 06511; jmartynow@melinta.com
* Correspondence: jsamarit@iupui.edu; Tel.: +1-317-274-6872

Received: 23 June 2018; Accepted: 14 July 2018; Published: date

Table of Contents

Figure S1. Proton NMR Spectrum of **19a** in $CDCl_3$ ... 5

Figure S2. Carbon-13 NMR Spectrum of **19a** in $CDCl_3$ ... 6

Figure S3. Proton NMR Spectrum of **19b** in $CDCl_3$ ... 7

Figure S4. Carbon-13 NMR Spectrum of **19b** in $CDCl_3$ ... 8

Figure S5. Proton NMR Spectrum of **19c** in $CDCl_3$ ... 9

Figure S6. Carbon-13 NMR Spectrum of **19c** in $CDCl_3$ ... 10

Figure S7. Proton NMR Spectrum of **α-21c** and **β-21c** in $CDCl_3$ ... 11

Figure S8. Carbon-13 NMR Spectrum of **α-21c** and **β-21c** in $CDCl_3$, ... 12

Figure S9. Proton NMR Spectrum of **24c** (earlier R_t) in CD_3OD ... 13

Figure S10. Carbon-13 NMR Spectrum of **24c** (earlier R_t) in CD_3OD ... 14

Figure S11. Proton NMR Spectrum of **24c** (later R_t) in $CDCl_3$ ... 15

Figure S12. Carbon-13 NMR Spectrum of **24c** (later R_t) in $CDCl_3$ ... 16

Figure S13. Proton NMR Spectrum of **β-26c** in CD_3OD ... 17

Figure S14. Carbon-13 NMR Spectrum of **β-26c** in CD_3OD ... 18

Figure S15. Proton NMR Spectrum of **31** in $CDCl_3$ ... 19

Figure S16. Carbon-13 NMR Spectrum of **31** in $CDCl_3$ 20
Figure S17. Proton NMR Spectrum of α**-33a** in $CDCl_3$ 21
Figure S18. Carbon-13 NMR Spectrum of α**-33a** in $CDCl_3$ 22
Figure S19. Proton NMR Spectrum of β**-33a** in $CDCl_3$........ 23
Figure S20. Carbon-13 NMR Spectrum of β**-33a** in $CDCl_3$........ 24
Figure S21. Proton NMR Spectrum of β**-33b** in $CDCl_3$ 25
Figure S22. Carbon-13 NMR Spectrum of β**-33b** in $CDCl_3$........ 26
Figure S23. Proton NMR Spectrum of α**-33b** in $CDCl_3$ 27
Figure S24. Carbon-13 NMR Spectrum of α**-33b** in $CDCl_3$ 28
Figure S25. Proton NMR Spectrum of β**-33c** in $CDCl_3$........ 29
Figure S26. Carbon-13 NMR Spectrum of β**-33c** in $CDCl_3$ 30
Figure S27. Proton NMR Spectrum of α**-33c** in $CDCl_3$........ 31
Figure S28. Carbon-13 NMR Spectrum of α**-33c** in $CDCl_3$........ 32
Figure S29. Proton NMR Spectrum of β**-34a** in $CDCl_3$........ 33
Figure S30. Carbon-13 NMR Spectrum of β**-34a** in $CDCl_3$........ 34
Figure S31. Proton NMR Spectrum of α**-34a** in $CDCl_3$ 35
Figure S32. Carbon-13 NMR Spectrum of α**-34a** in $CDCl_{3,}$........ 36
Figure S33. Proton NMR Spectrum of α**-35a** in $CDCl_3$ 37
Figure S34. Carbon-13 NMR Spectrum of α**-35a** in $CDCl_3$ 38
Figure S35. Proton NMR Spectrum of β**-35a** in $CDCl_3$........ 39
Figure S36. Carbon-13 NMR Spectrum of β**-35a** in $CDCl_3$........ 40
Figure S37. Proton NMR Spectrum of β**-35b** in $CDCl_3$ 41
Figure S38. Carbon-13 NMR Spectrum of β**-35b** in $CDCl_3$........ 42
Figure S39. Proton NMR Spectrum of α**-35b** in $CDCl_3$ 43
Figure S40. Carbon-13 NMR Spectrum of α**-35b** in $CDCl_3$ 44
Figure S41. Proton NMR Spectrum of β**-35c** in CDCl3 45
Figure S42. Carbon-13 NMR Spectrum of β**-35c** in $CDCl_3$ 46
Figure S43. Proton NMR Spectrum of α**-35c** in CDCl3 47
Figure S44. Carbon-13 NMR Spectrum of α**-35c** in $CDCl_3$........ 48
Figure S45. Proton NMR Spectrum of β**-36a** in $CDCl_3$........ 49
Figure S46. Carbon-13 NMR Spectrum of β**-36a** in $CDCl_3$........ 50
Figure S47. Proton NMR Spectrum of α**-36a** in $CDCl_3$ 51

Figure S48. Carbon-13 NMR Spectrum of **α-36a** in $CDCl_3$ 52
Figure S49. Proton NMR Spectrum of **β-36b** in $CDCl_3$ 53
Figure S50. Carbon-13 NMR Spectrum of **β-36b** in $CDCl_3$ 54
Figure S51. Proton NMR Spectrum of **α-36b** in $CDCl_3$ 55
Figure S52. Carbon-13NMR Spectrum of **α-36b** in $CDCl_3$ 56
Figure S53. Proton NMR Spectrum of **α-36c** in $CDCl_3$ 57
Figure S54. Carbon-13 NMR Spectrum of **α-36c** in $CDCl_3$ 58
Figure S55. Proton NMR Spectrum of **β-36c** in $CDCl_3$ 59
Figure S56. Carbon-13 NMR Spectrum of **β-36c** in $CDCl_3$ 60
Figure S57. Proton NMR Spectrum of **α-30b** in $CDCl_3$ 61
Figure S58. Carbon-13 NMR Spectrum of **α-30b** in $CDCl_3$ 62
Figure S59. Proton NMR Spectrum of **β-30b** in $CDCl_3$ 63
Figure S60. Carbon-13 NMR Spectrum of **β-30b** in $CDCl_3$ 64
Figure S61. Proton NMR Spectrum of **β-30c** in $CDCl_3$ 65
Figure S62. Carbon-13 NMR Spectrum of **β-30c** in $CDCl_3$ 66
Figure S63. Proton NMR Spectrum of **α-30c** in $CDCl_3$ 67
Figure S64. Carbon-13 NMR Spectrum of **α-30c** in $CDCl_3$ 68
Figure S65. Proton NMR Spectrum of **α-37b** in $CDCl_3$ 69
Figure S66. Carbon-13 NMR Spectrum of **α-37b** in $CDCl_3$ 70
Figure S67. Proton NMR Spectrum of **β-37b** in $CDCl_3$ 71
Figure S68. Carbon-13 NMR Spectrum of **β-37b** in $CDCl_3$ 72
Figure S69. Proton NMR Spectrum of (Fmoc-Ala)$_2$O in $CDCl_3$ with Diisopropylurea (DIU) 73
Figure S70. Proton NMR Spectrum of Boc-Cys(Trt)-Leu-OMe in $CDCl_3$ 74
Figure S71. Carbon-13 NMR Spectrum of Boc-Cys(Trt)-Leu-OMe in $CDCl_3$ 75

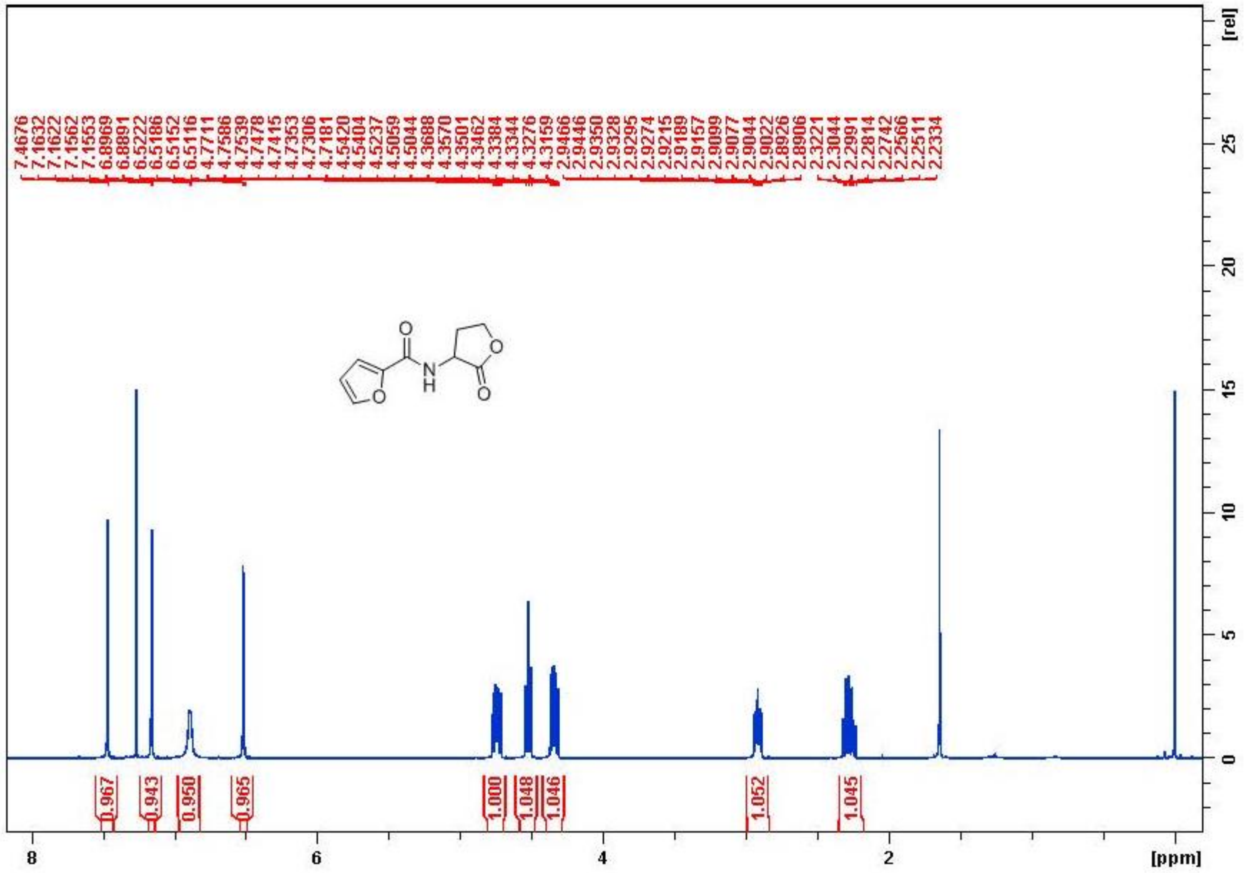

Figure S1. Proton NMR Spectrum of **19a** in $CDCl_3$

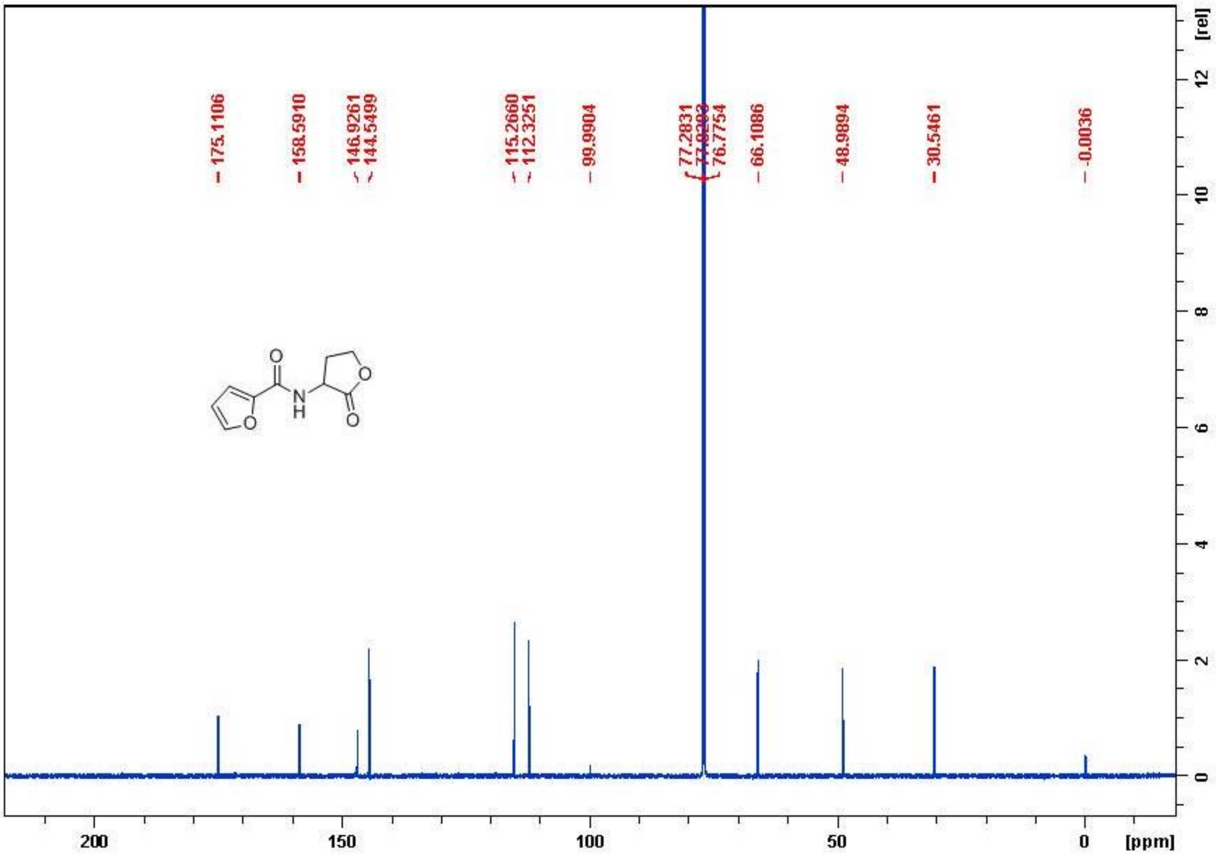

Figure S2. Carbon-13 NMR Spectrum of **19a** in $CDCl_3$

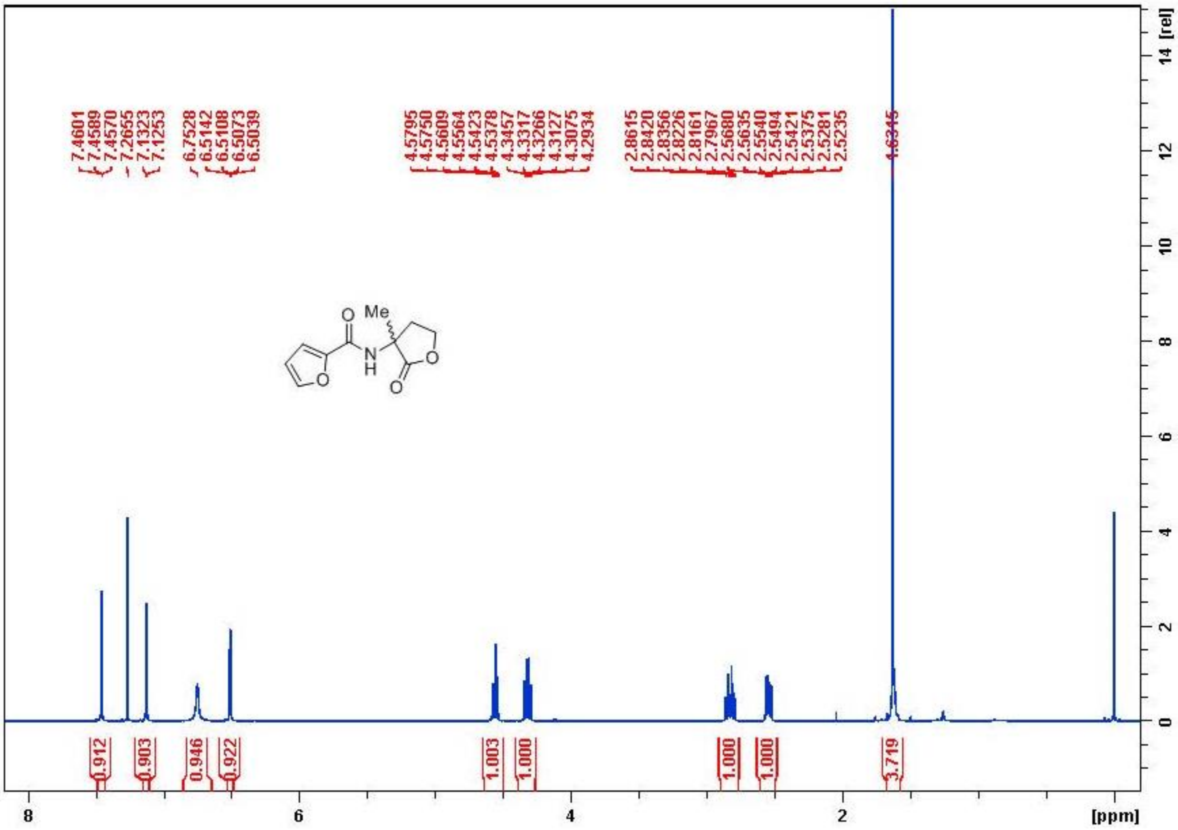

Figure S3. Proton NMR Spectrum of **19b** in $CDCl_3$

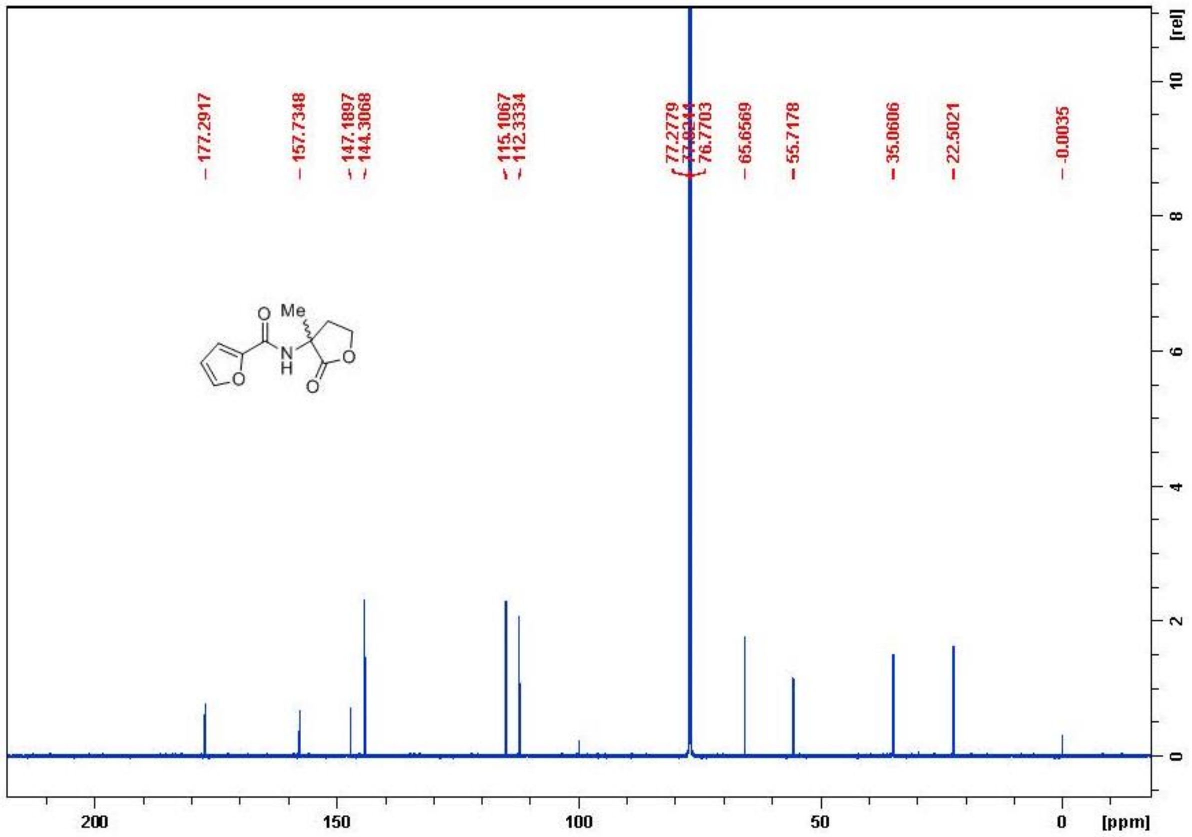

Figure S4. Carbon-13 NMR Spectrum of **19b** in $CDCl_3$

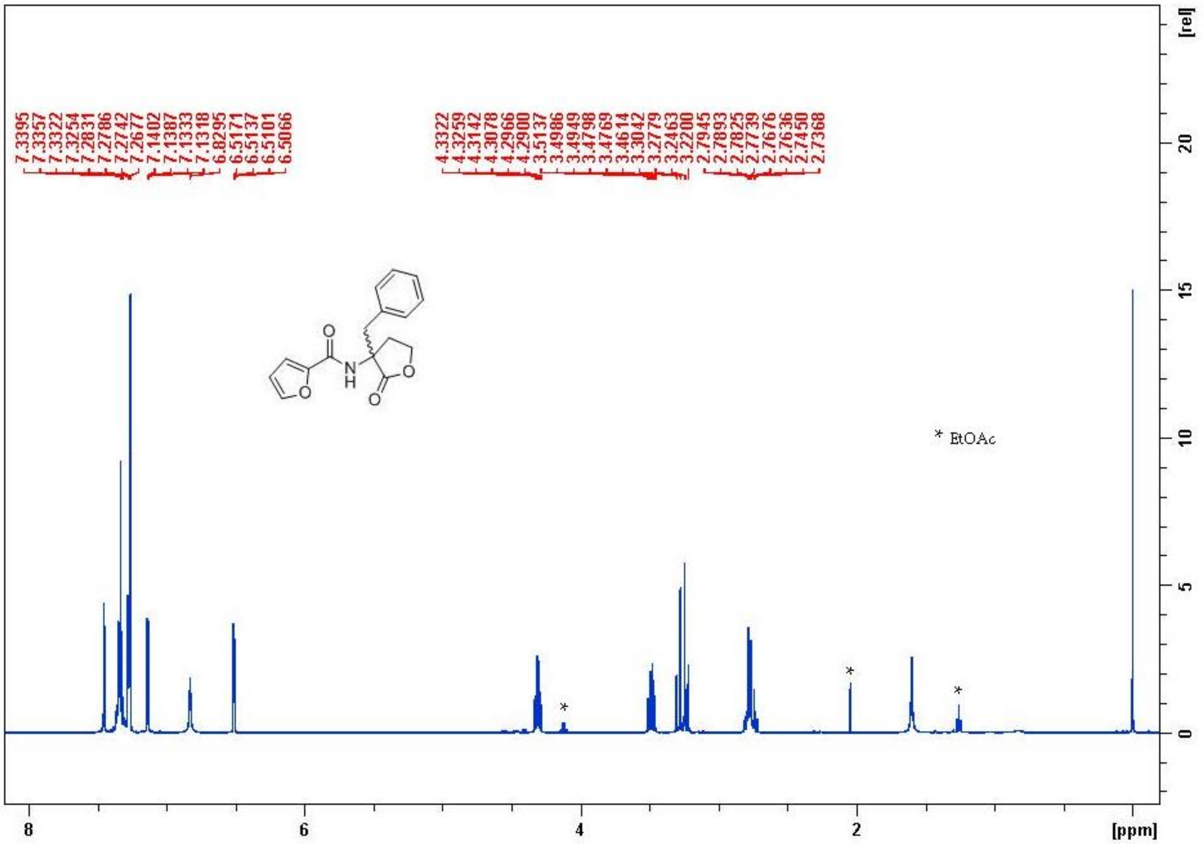

Figure S5. Proton NMR Spectrum of **19c** in $CDCl_3$

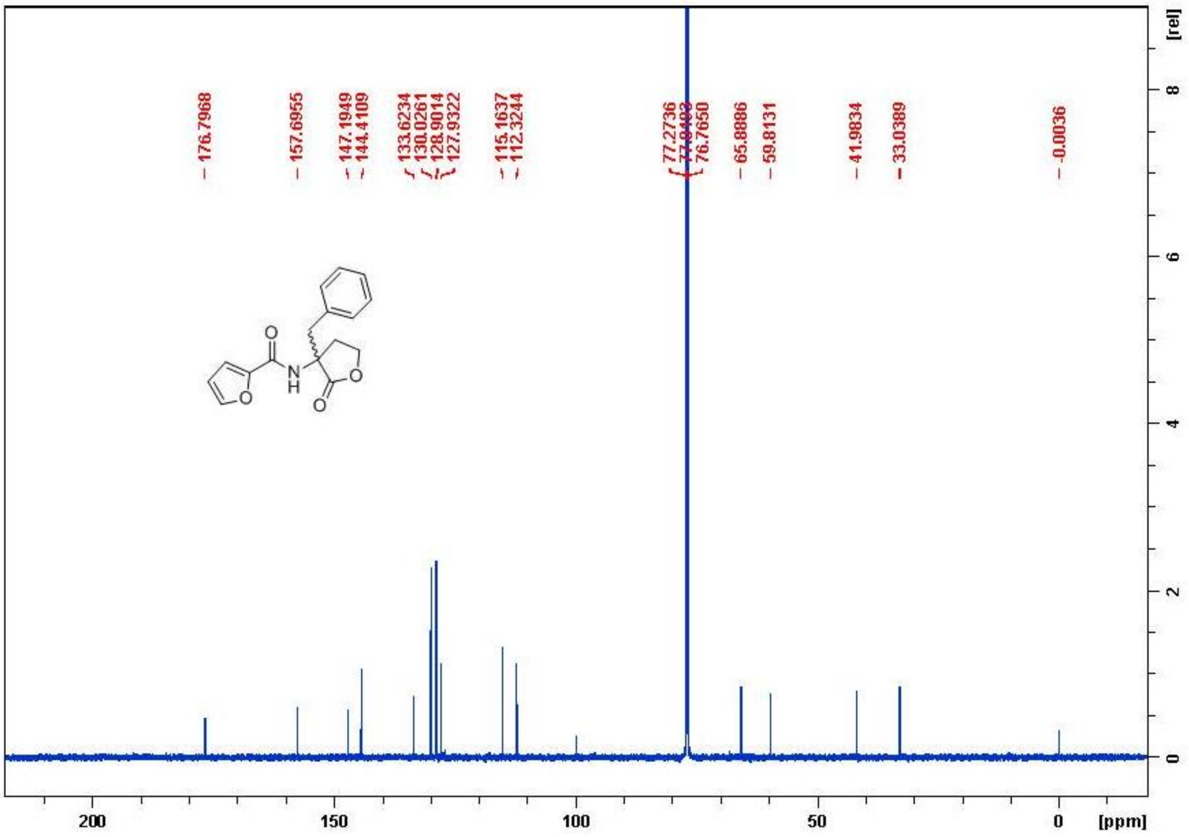

Figure S6. Carbon-13 NMR Spectrum of **19c** in $CDCl_3$

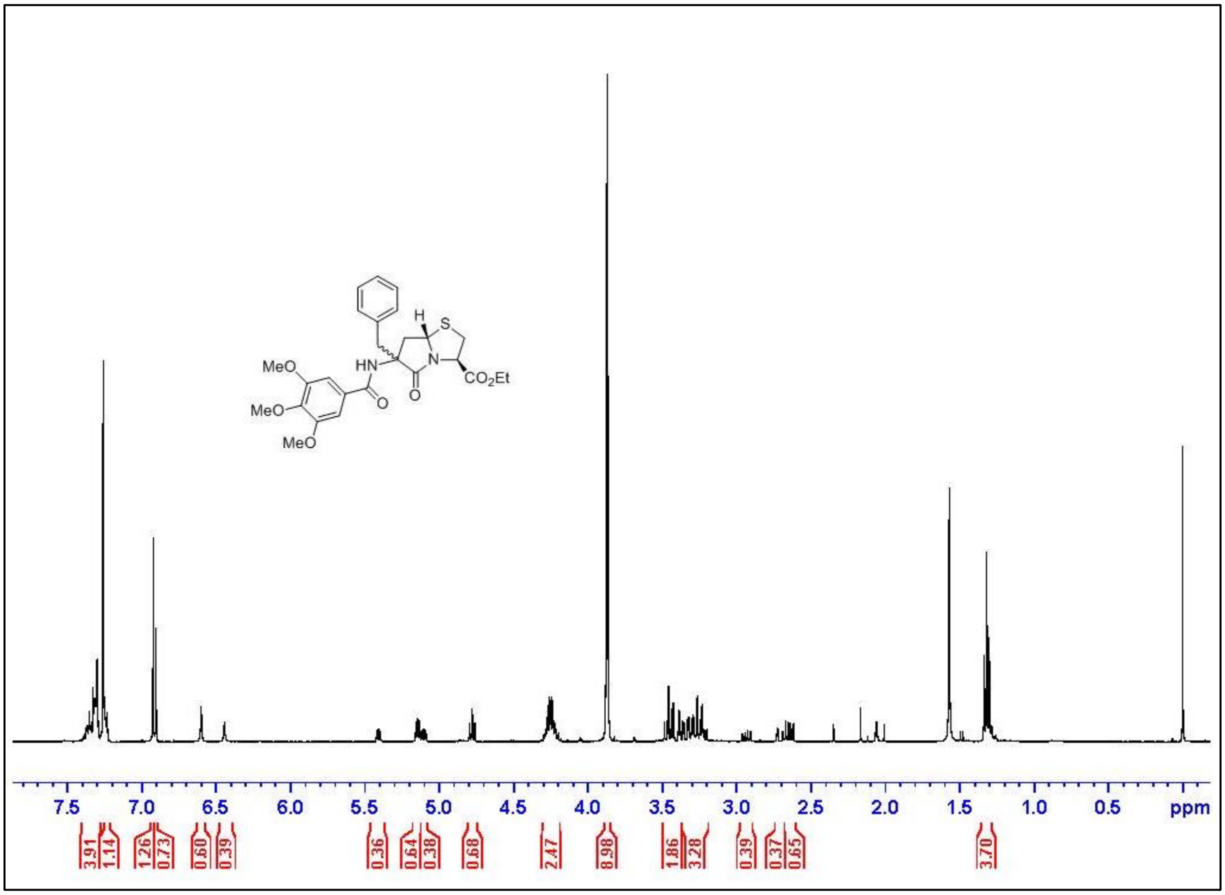

Figure S7. Proton NMR Spectrum of **α-21c** and **β-21c** in $CDCl_3$

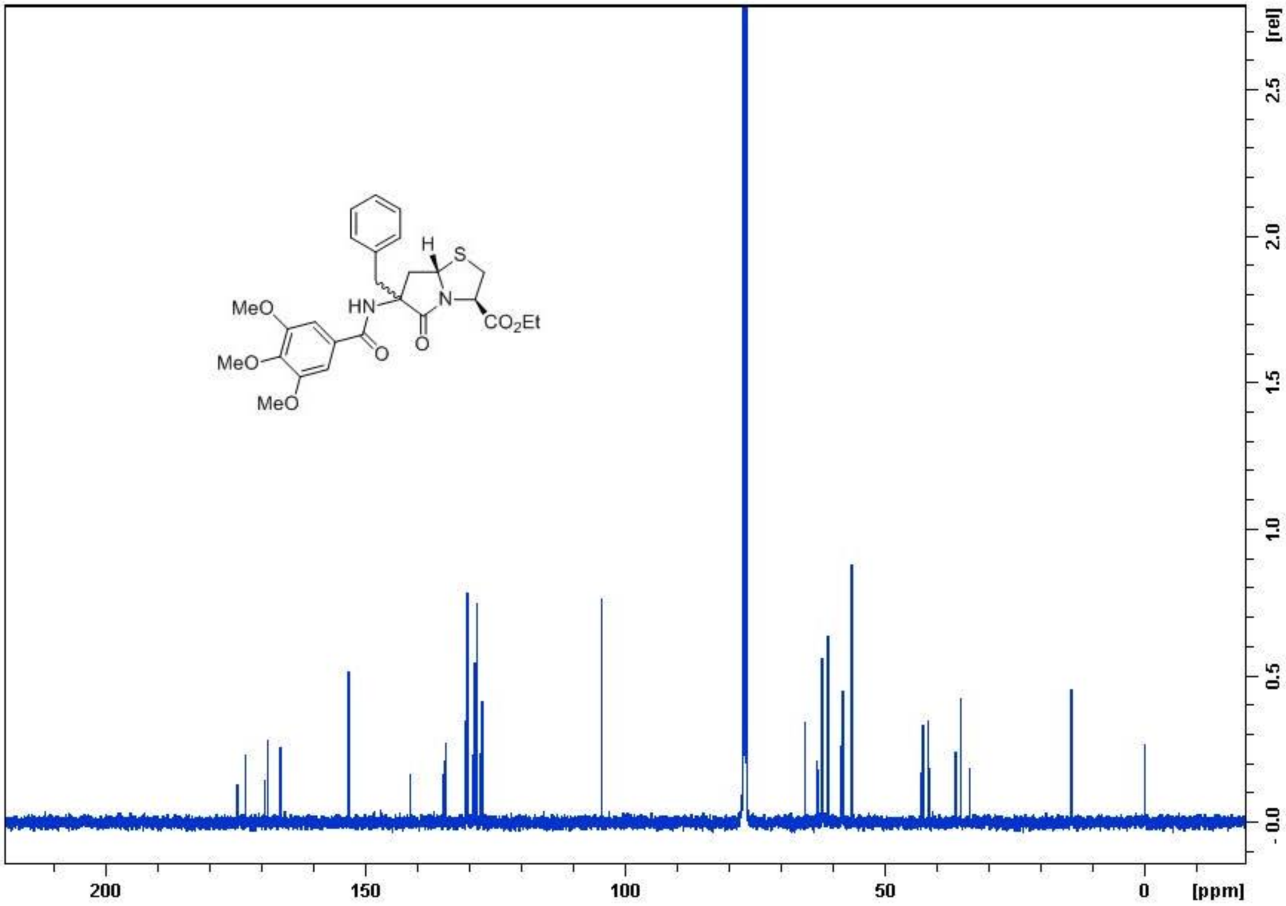

Figure S8. Carbon-13 NMR Spectrum of **α-21c** and **β-21c** in $CDCl_3$

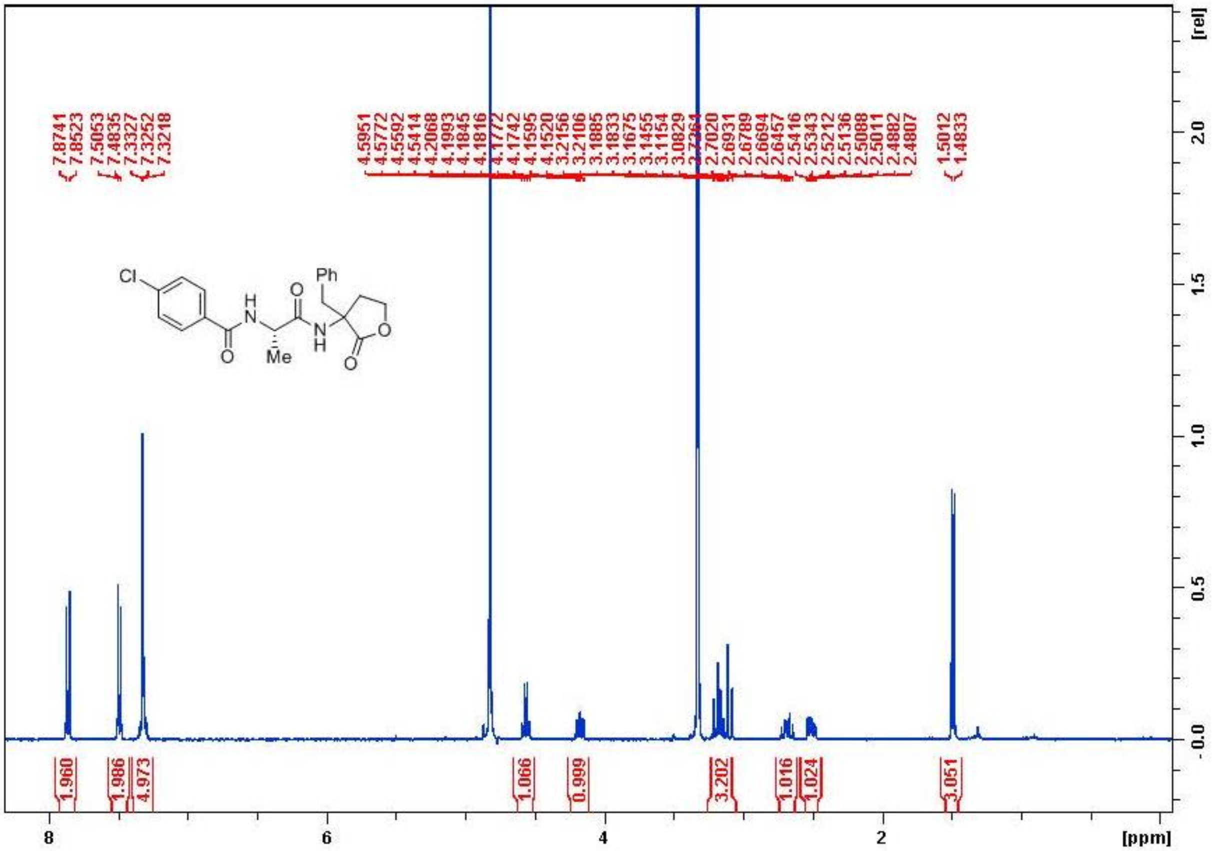

Figure S9. Proton NMR Spectrum of **24c** (earlier R_t) in CD_3OD

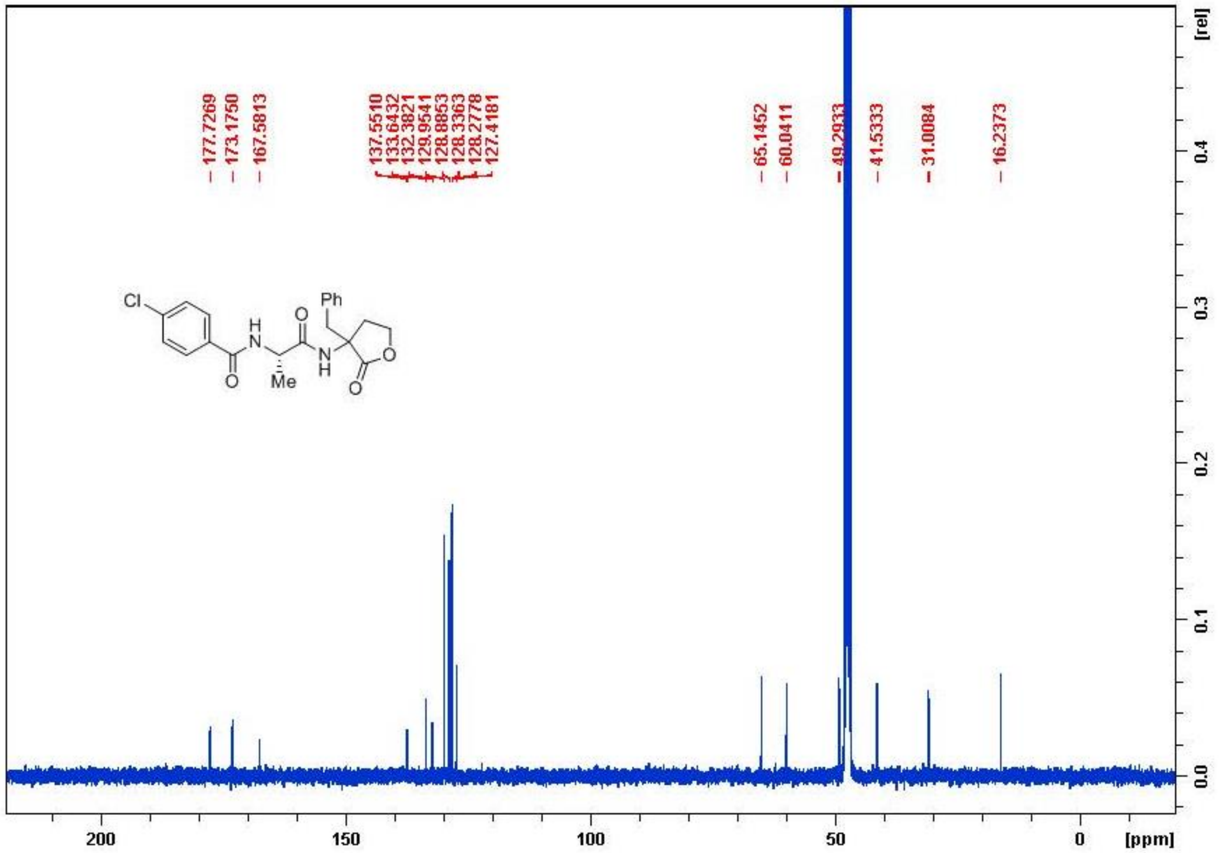

Figure S10. Carbon-13 NMR Spectrum of **24c** (earlier R_t) in CD_3OD

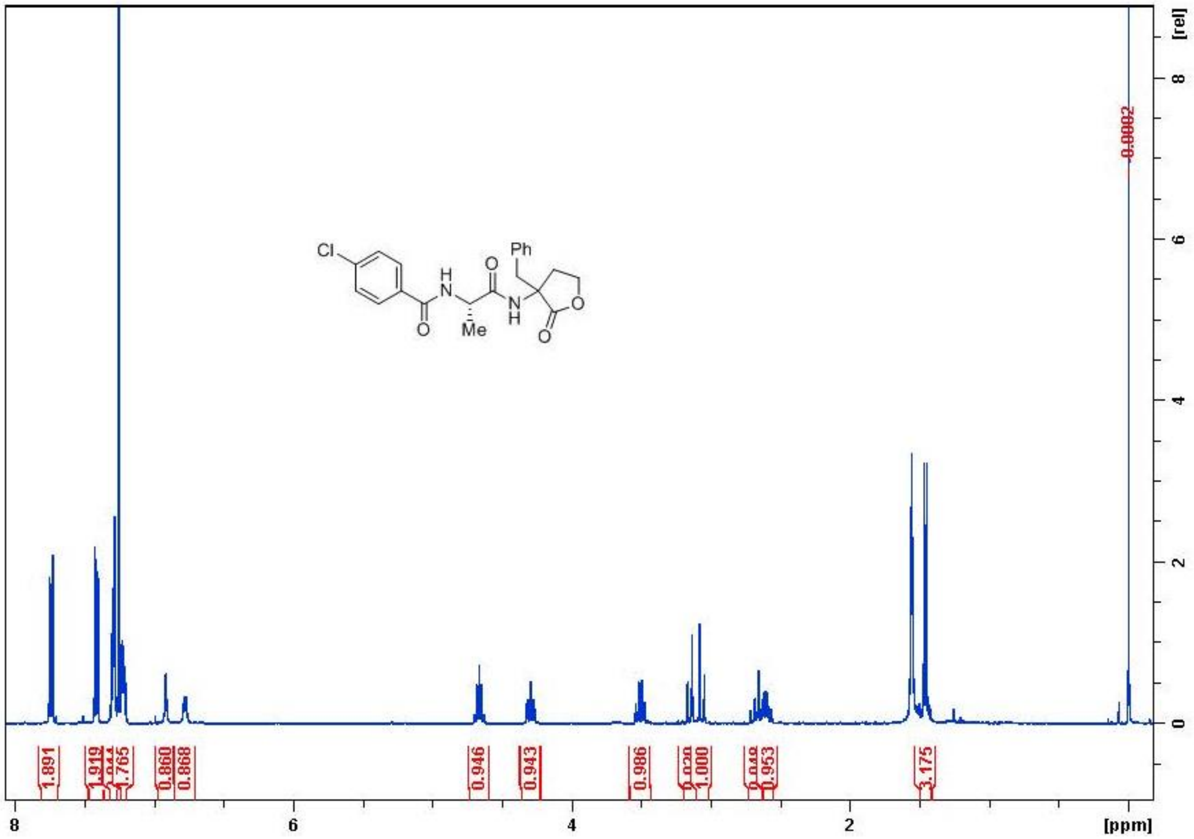

Figure S11. Proton NMR Spectrum of **24c** (later R_t) in $CDCl_3$

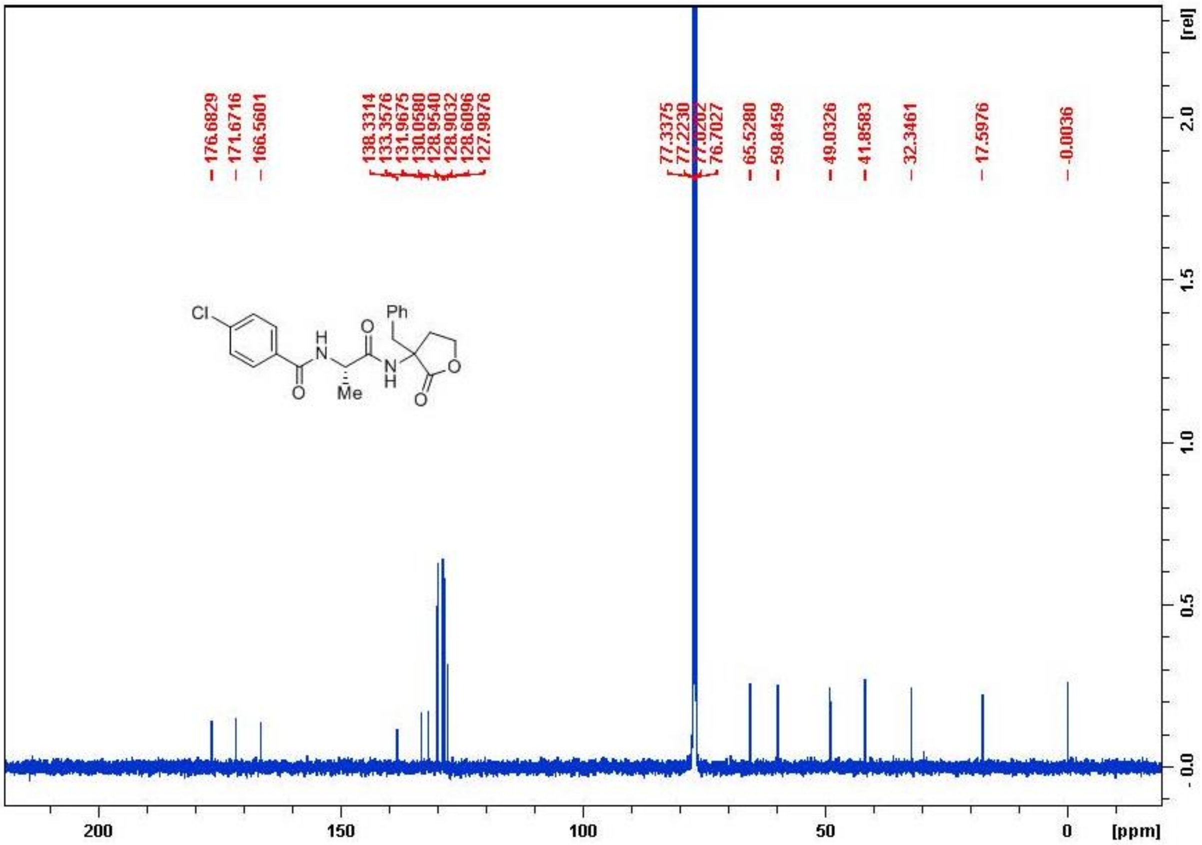

Figure S12. Carbon-13 NMR Spectrum of **24c** (later R_t) in $CDCl_3$

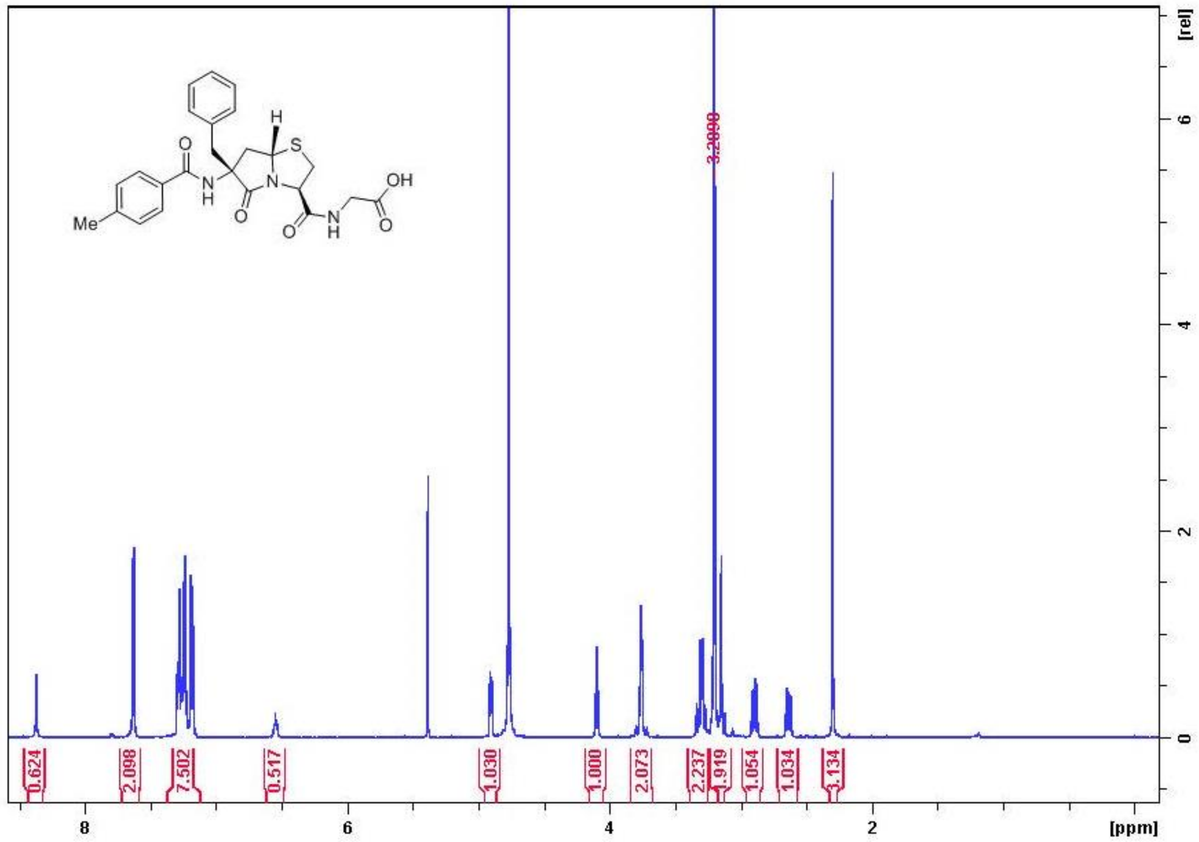

Figure S13. Proton NMR Spectrum of **β-26c** in CD_3OD

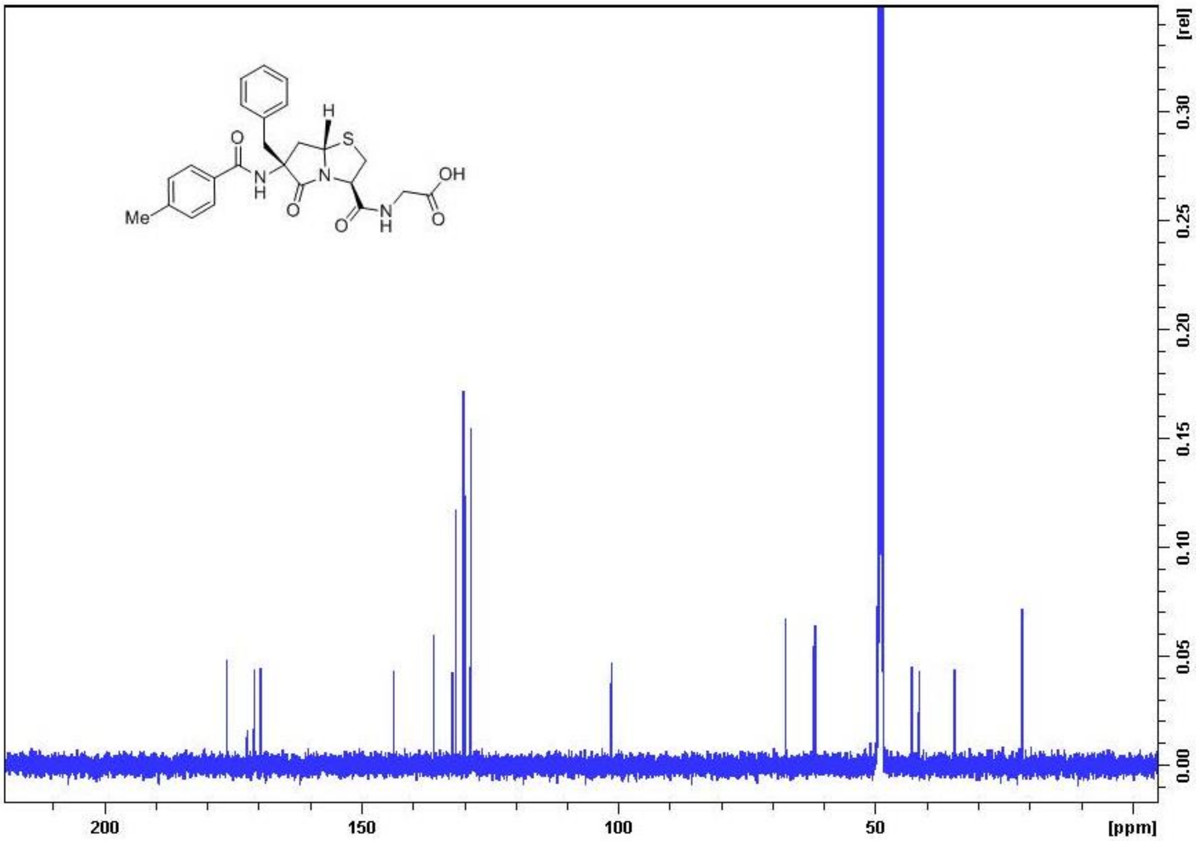

Figure S14. Carbon-13 NMR Spectrum of **β-26c** in CD_3OD

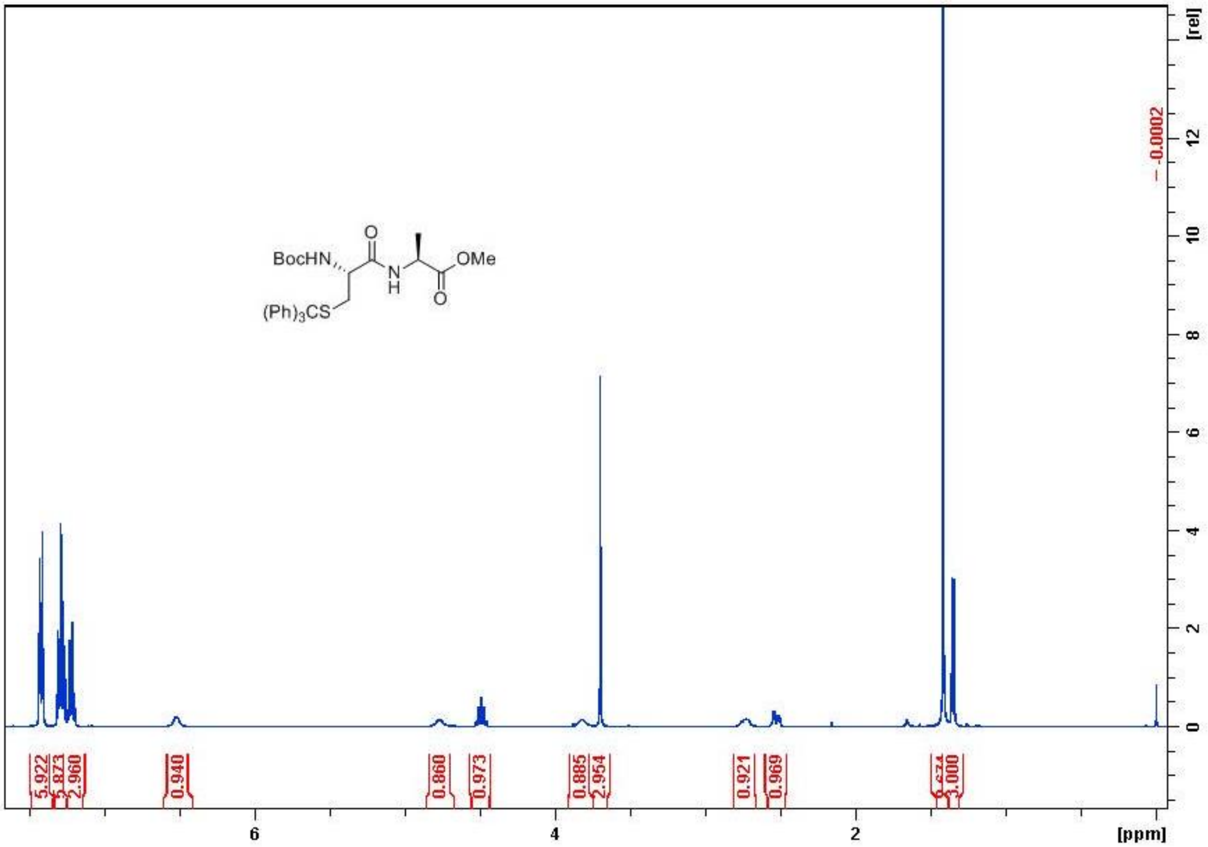

Figure S15. Proton NMR Spectrum of **31** in $CDCl_3$

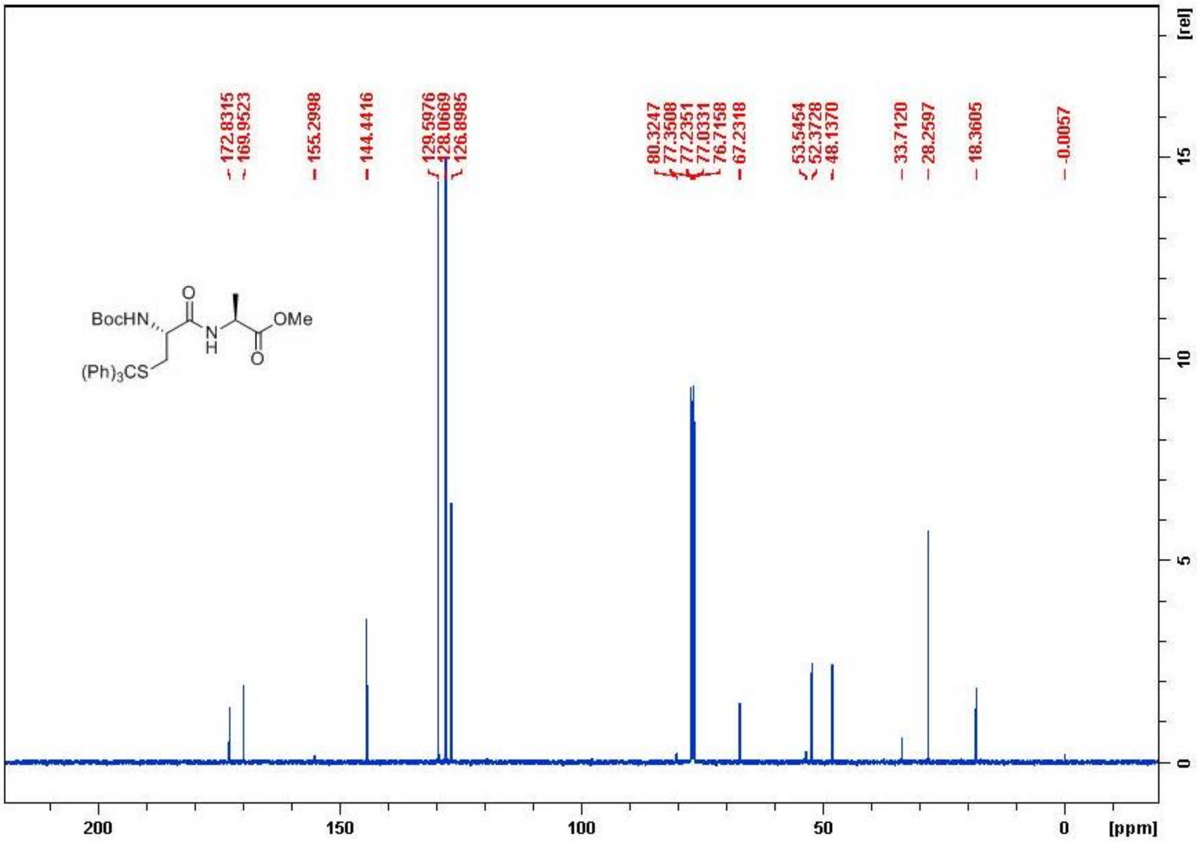

Figure S16. Carbon-13 NMR Spectrum of **31** in $CDCl_3$

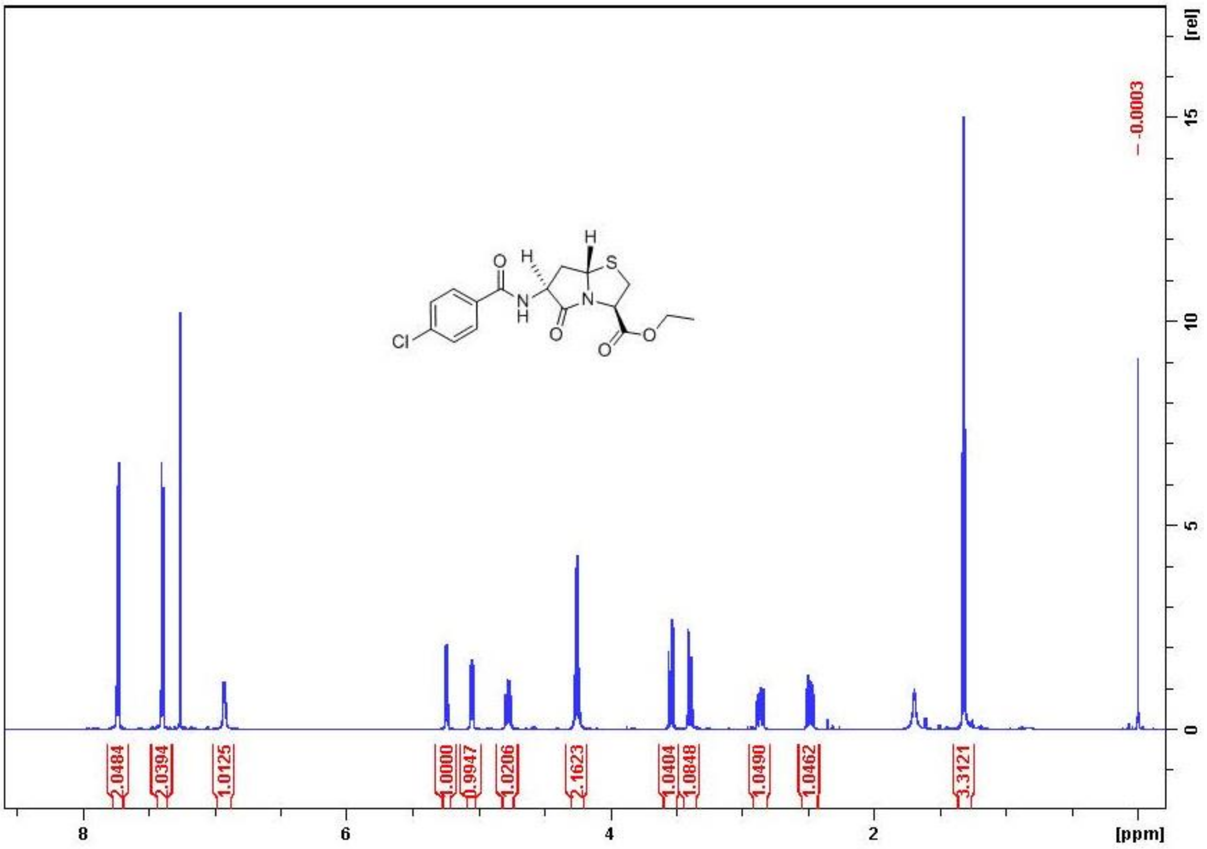

Figure S17. Proton NMR Spectrum of **α-33a** in $CDCl_3$

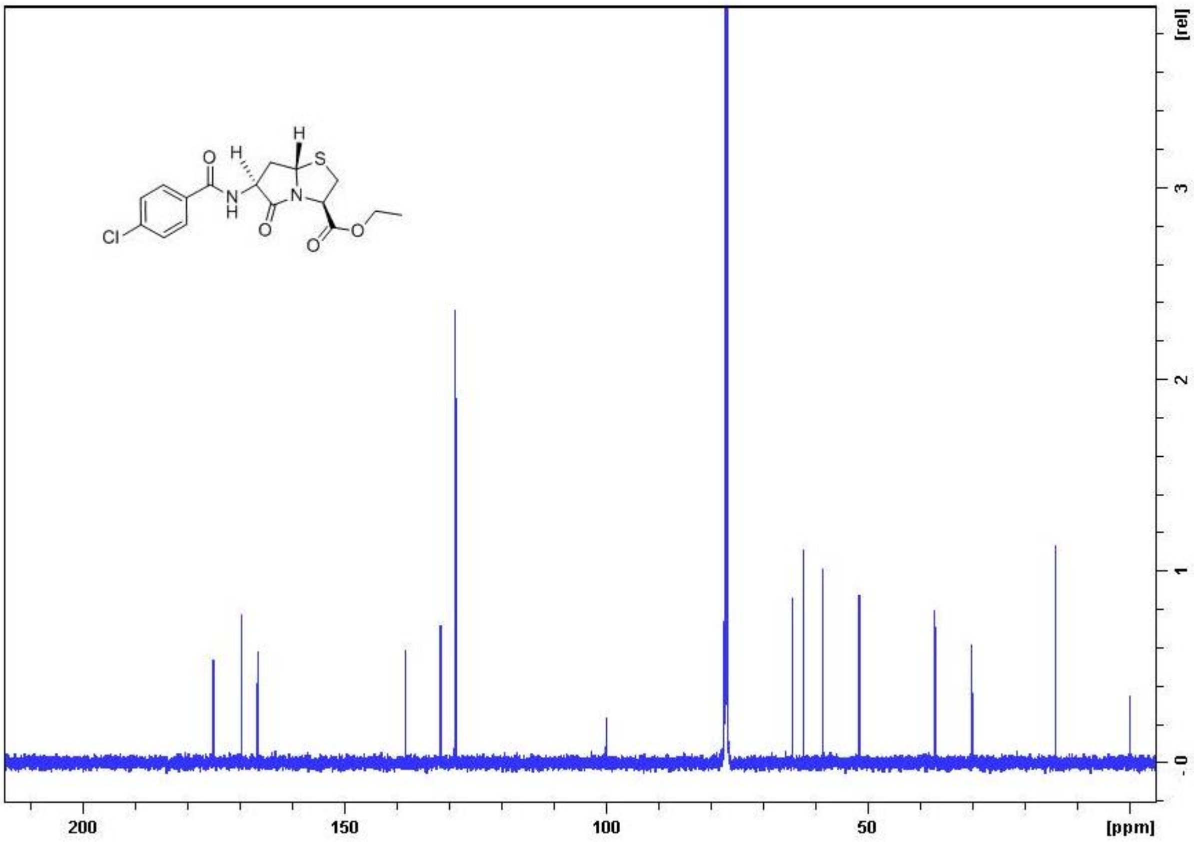

Figure S18. Carbon-13 NMR Spectrum of **α-33a** in $CDCl_3$

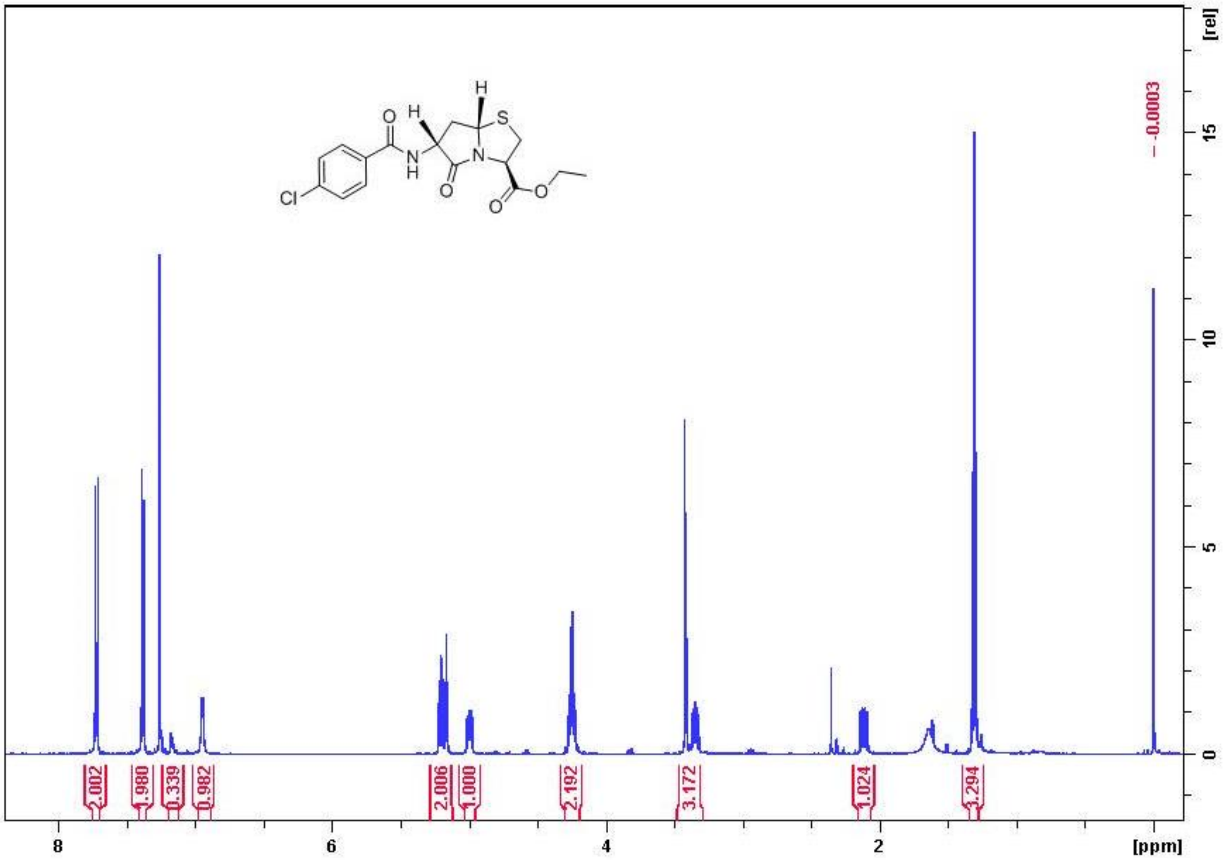

Figure S19. Proton NMR Spectrum of **β-33a** in $CDCl_3$

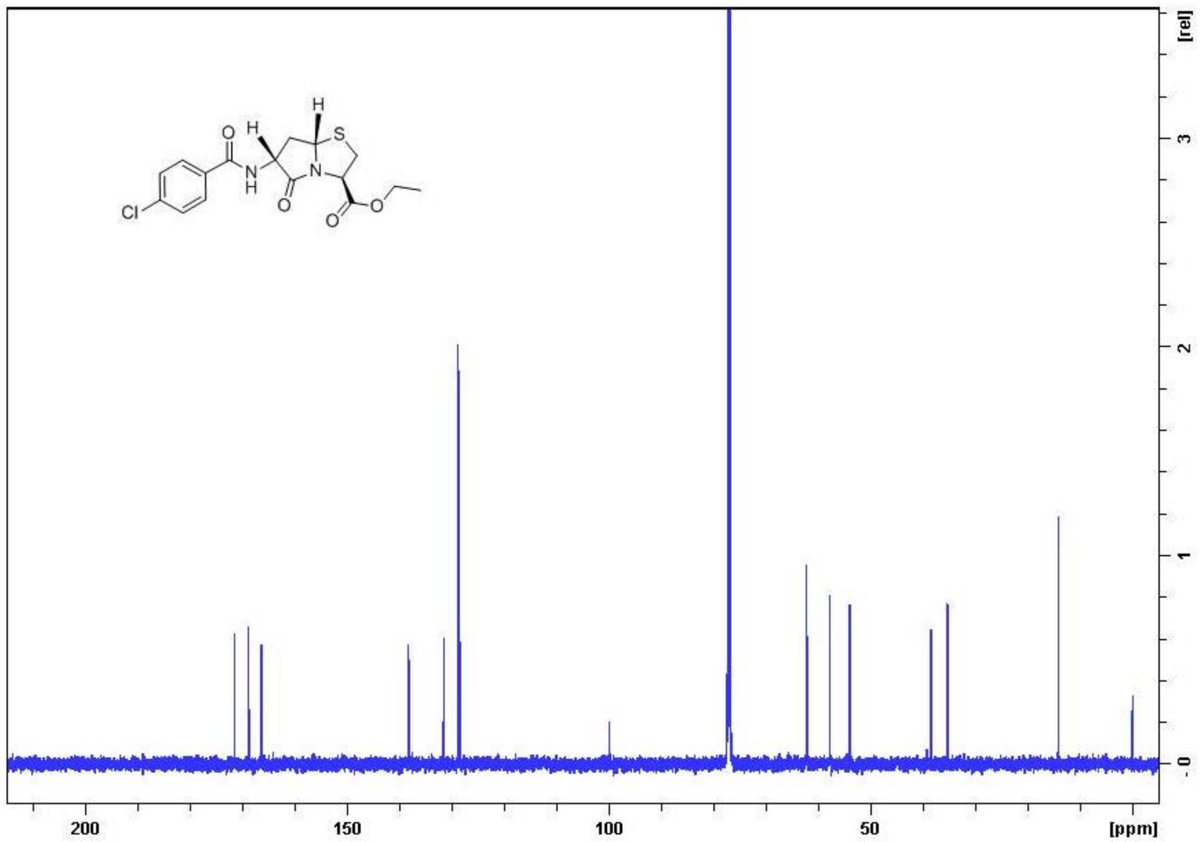

Figure S20. Carbon-13 NMR Spectrum of **β-33a** in $CDCl_3$

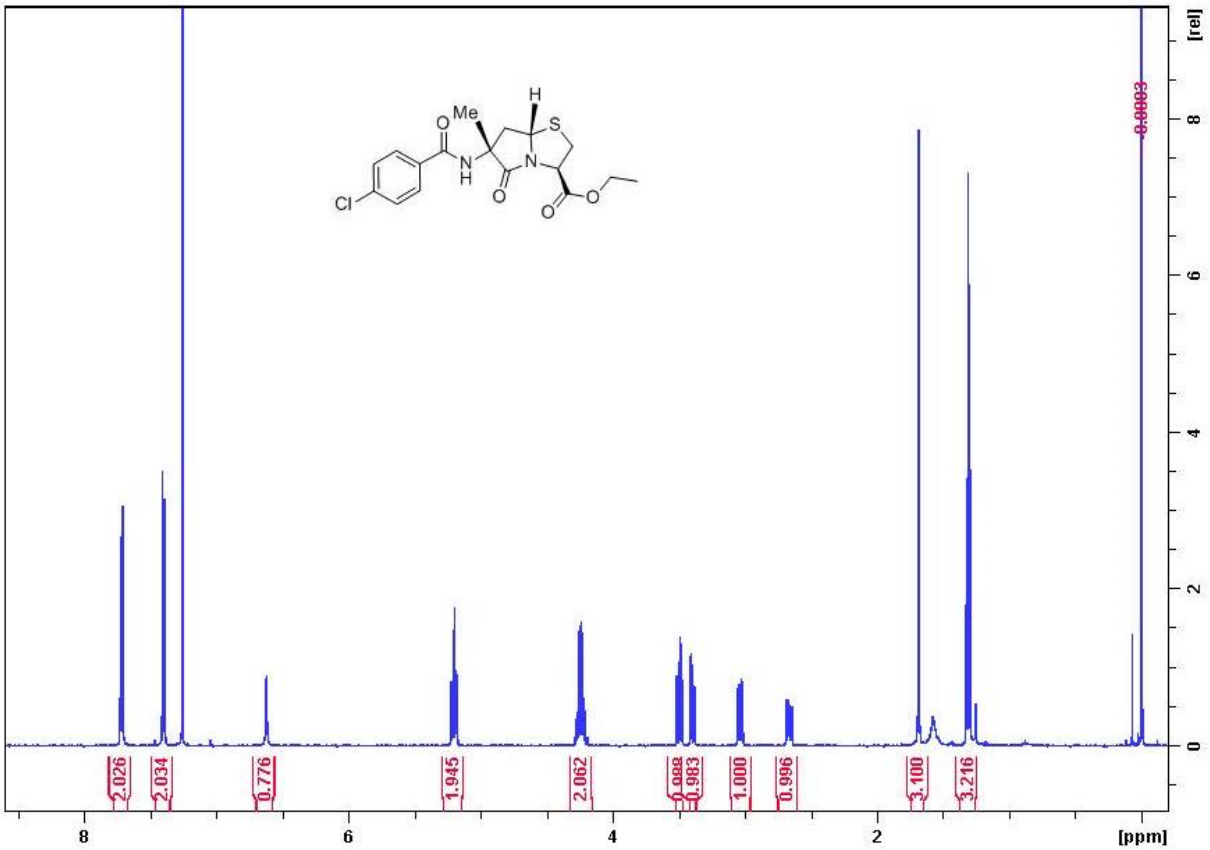

Figure S21. Proton NMR Spectrum of **β-33b** in $CDCl_3$

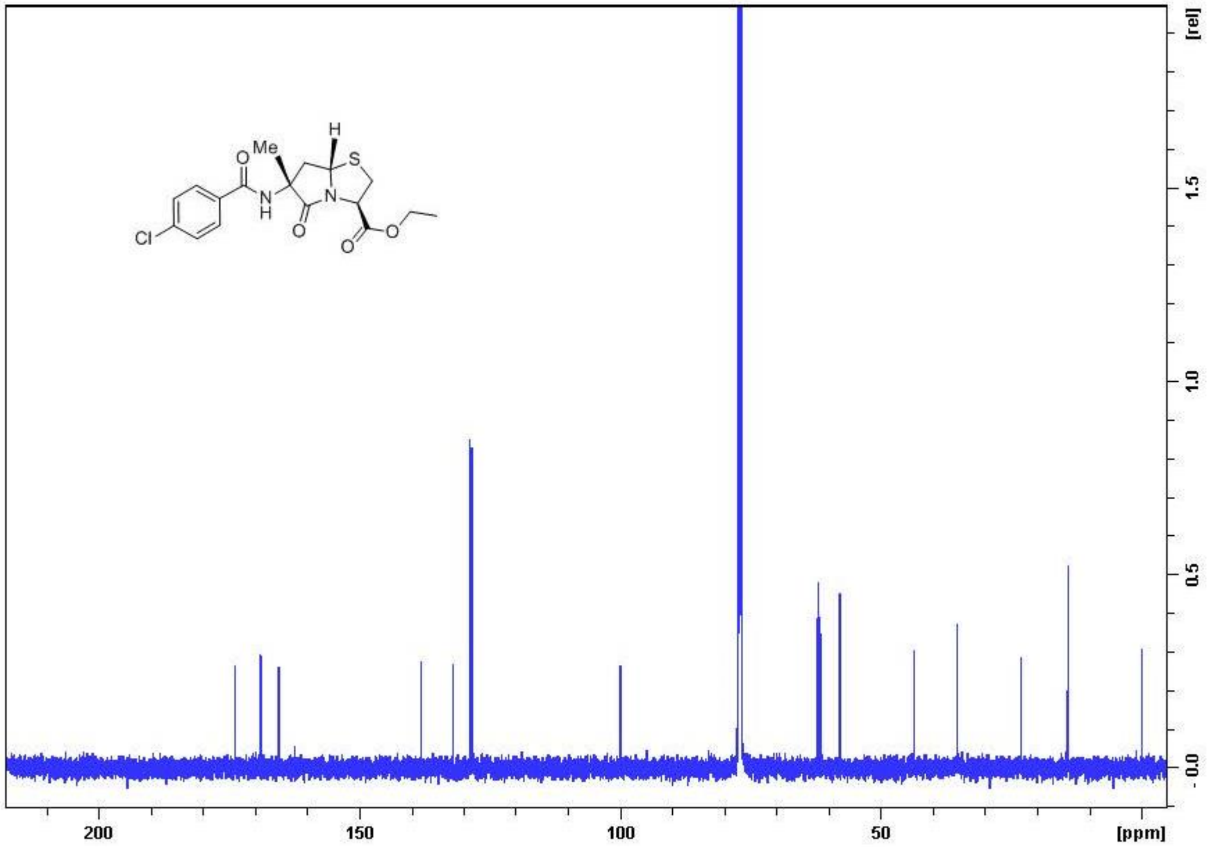

Figure S22. Carbon-13 NMR Spectrum of **β-33b** in $CDCl_3$

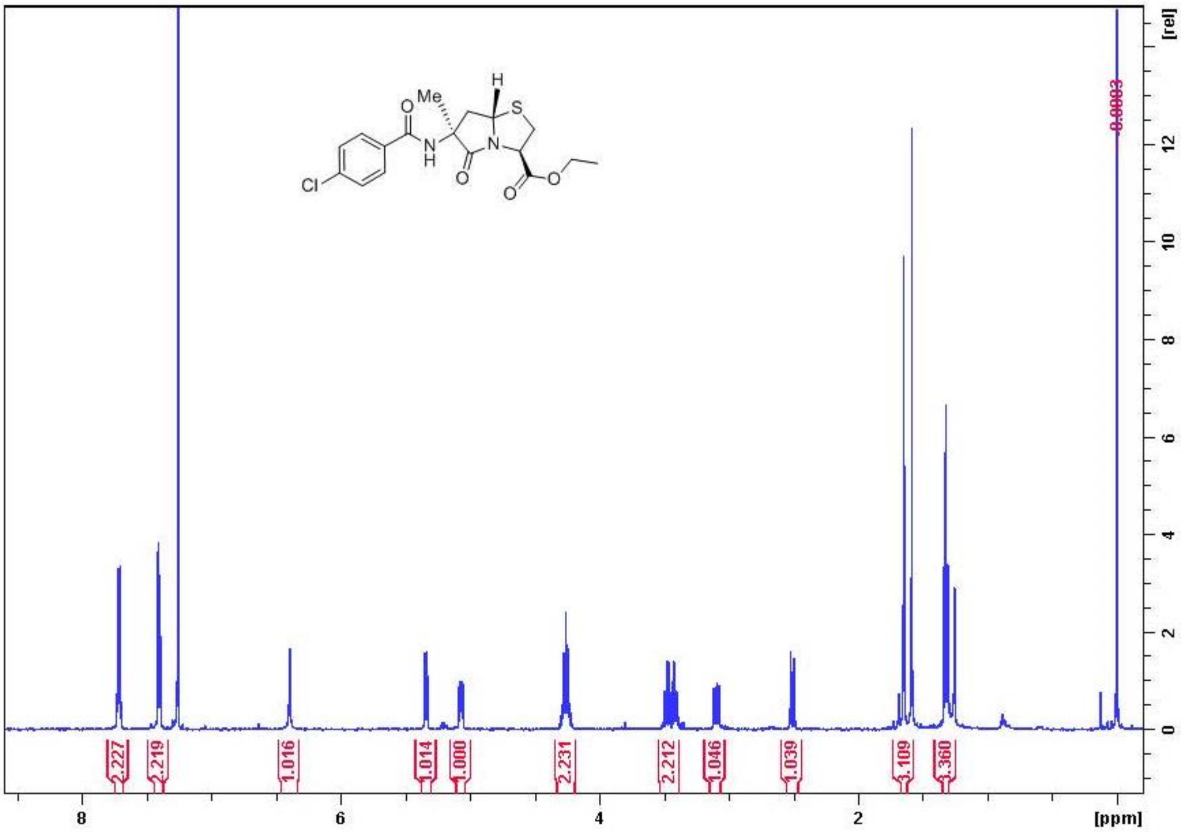

Figure S23. Proton NMR Spectrum of **α-33b** in $CDCl_3$

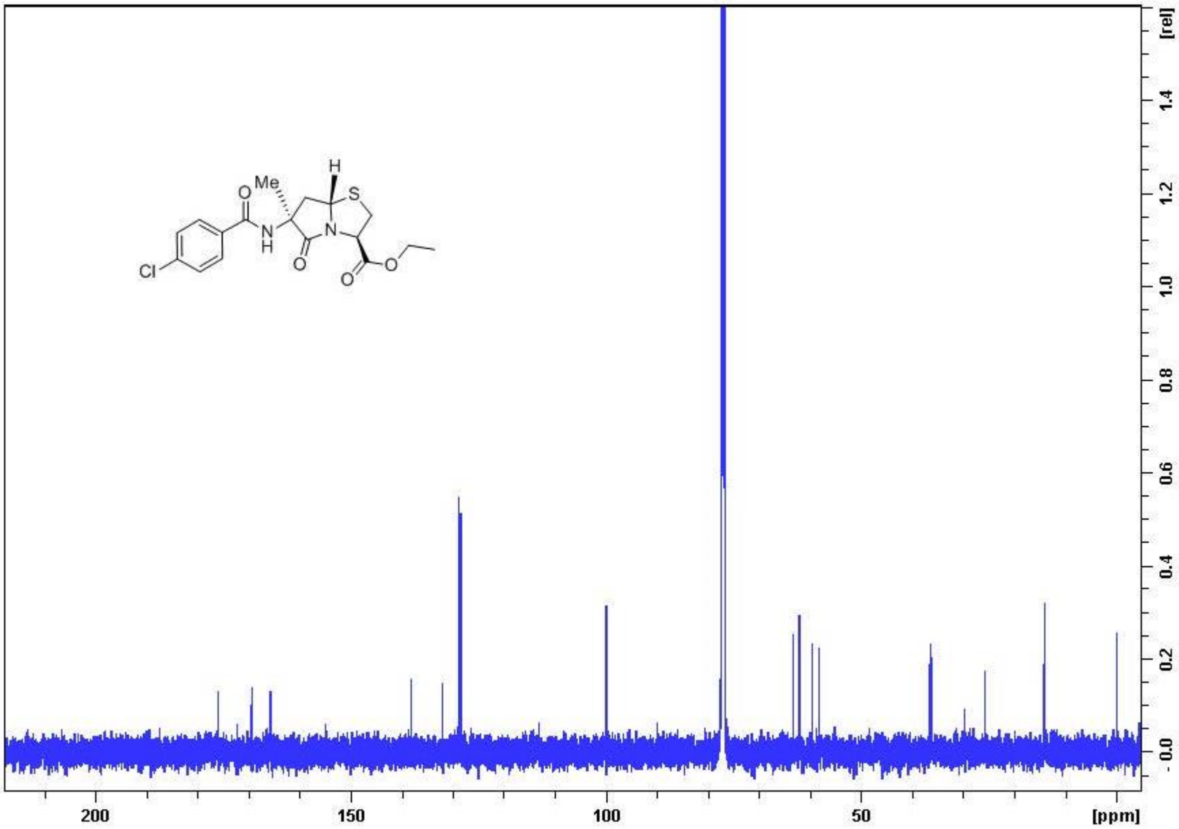

Figure S24. Carbon-13 NMR Spectrum of **α-33b** in $CDCl_3$

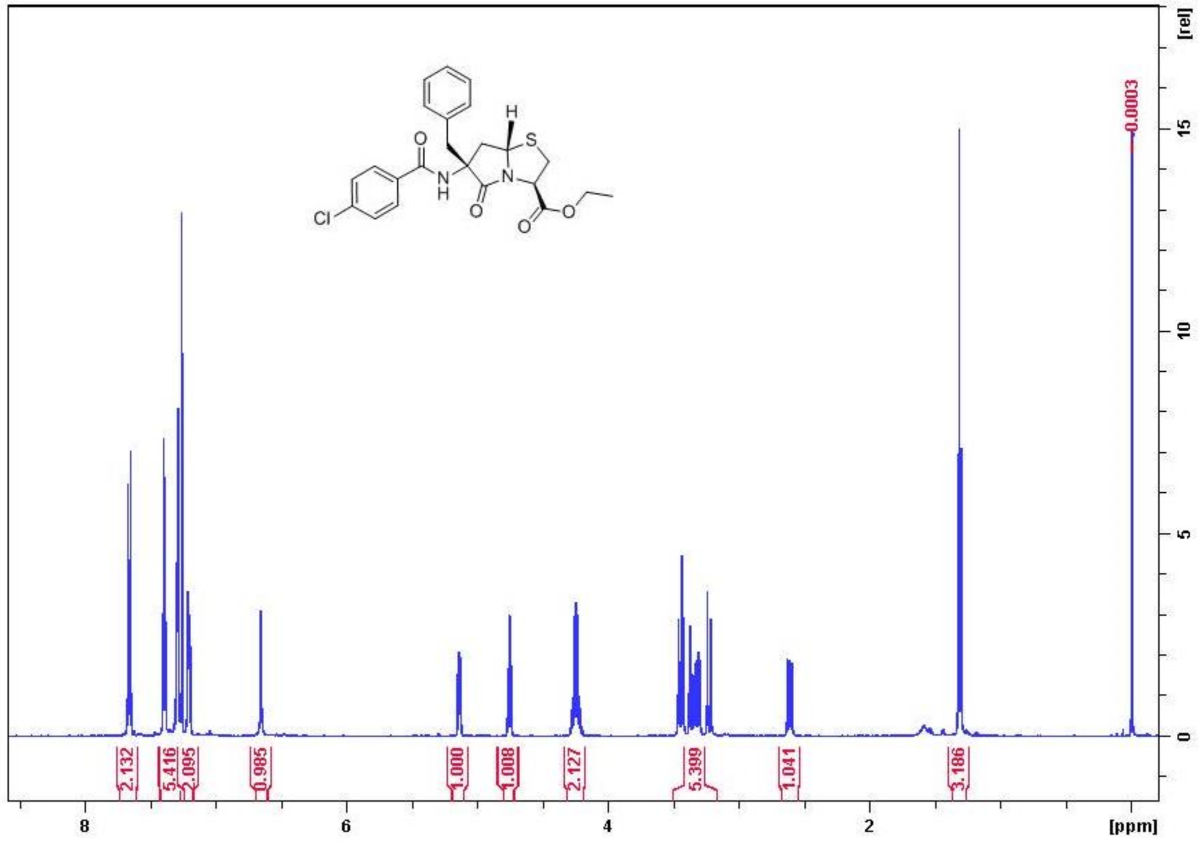

Figure S25. Proton NMR Spectrum of **β-33c** in $CDCl_3$

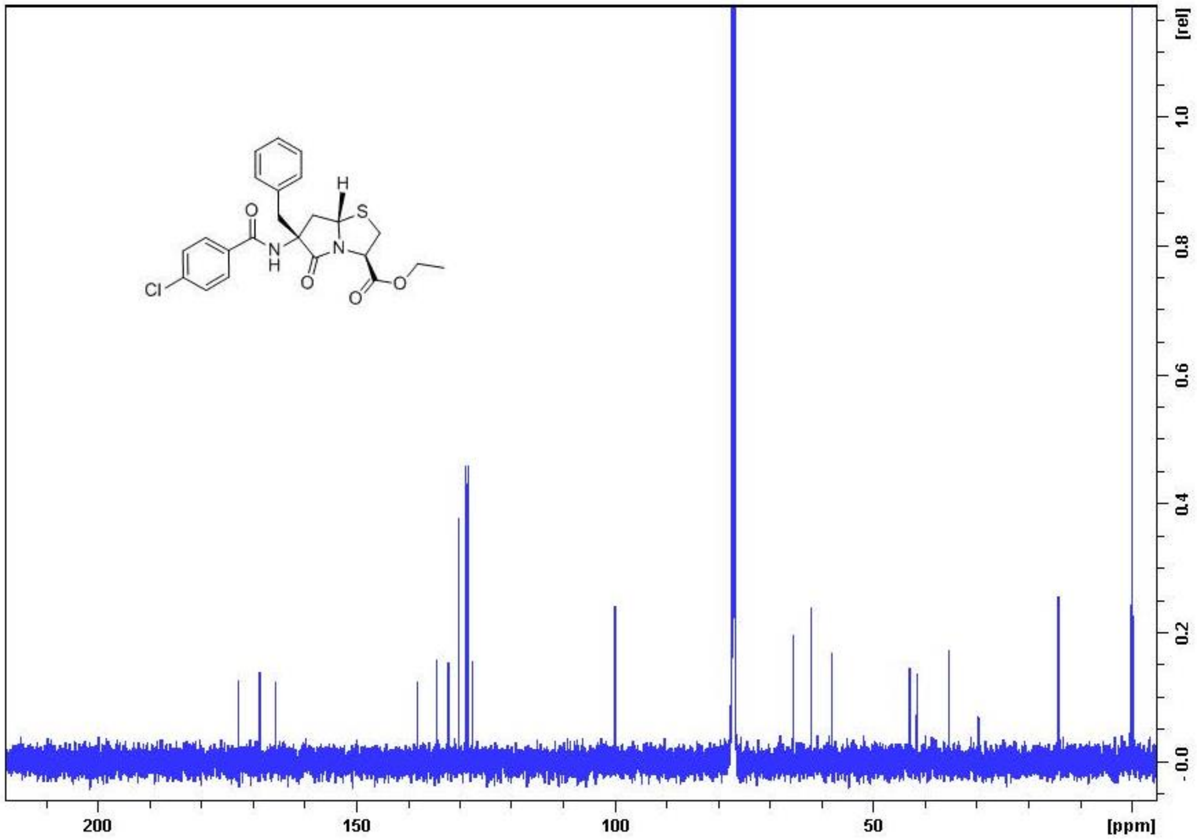

Figure S26. Carbon-13 NMR Spectrum of **β-33c** in $CDCl_3$

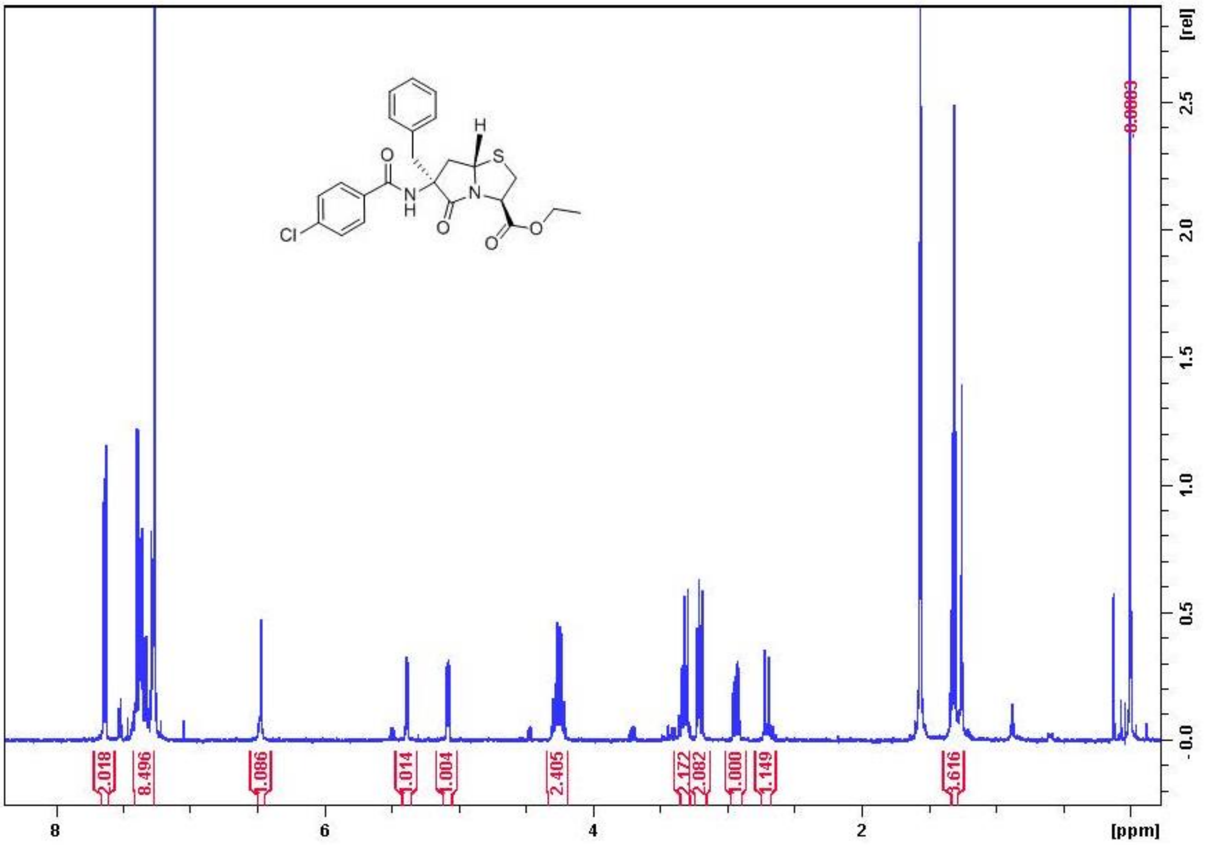

Figure S27. Proton NMR Spectrum of **α-33c** in $CDCl_3$

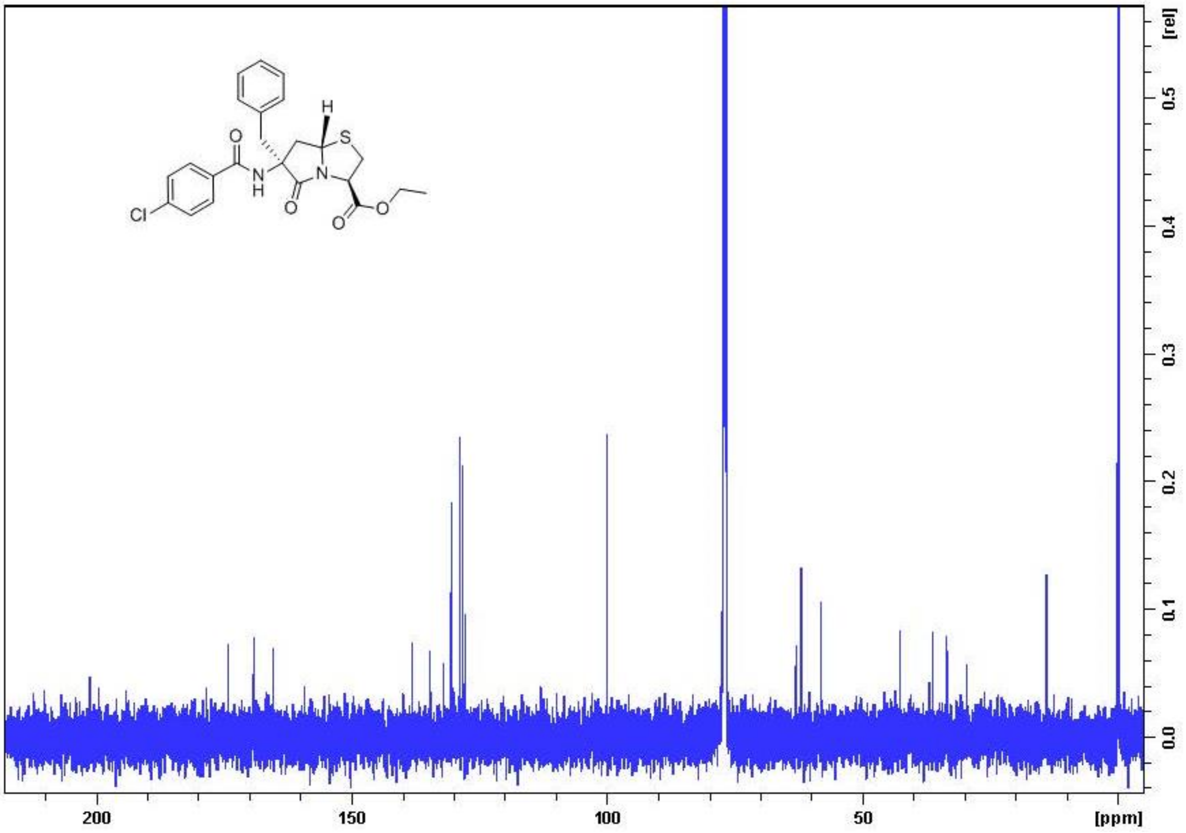

Figure S28. Carbon-13 NMR Spectrum of **α-33c** in $CDCl_3$

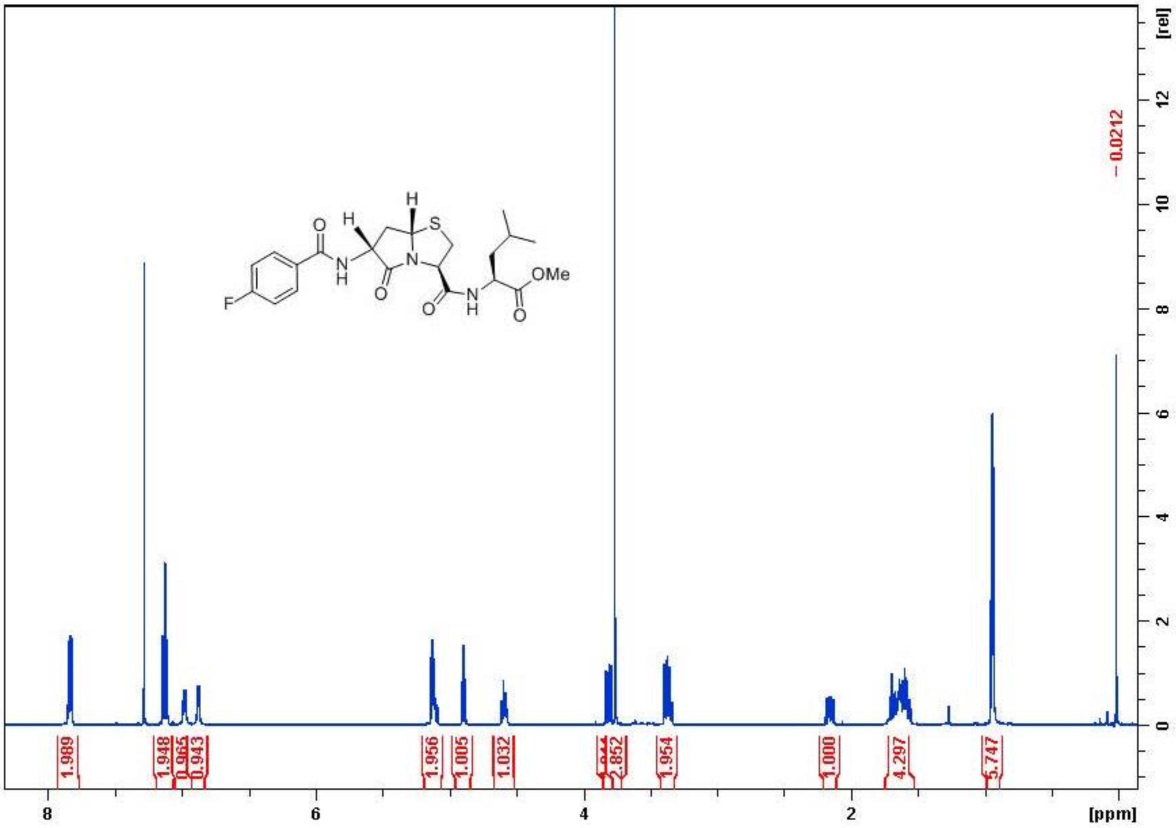

Figure S29. Proton NMR Spectrum of **β-34a** in CDCl3

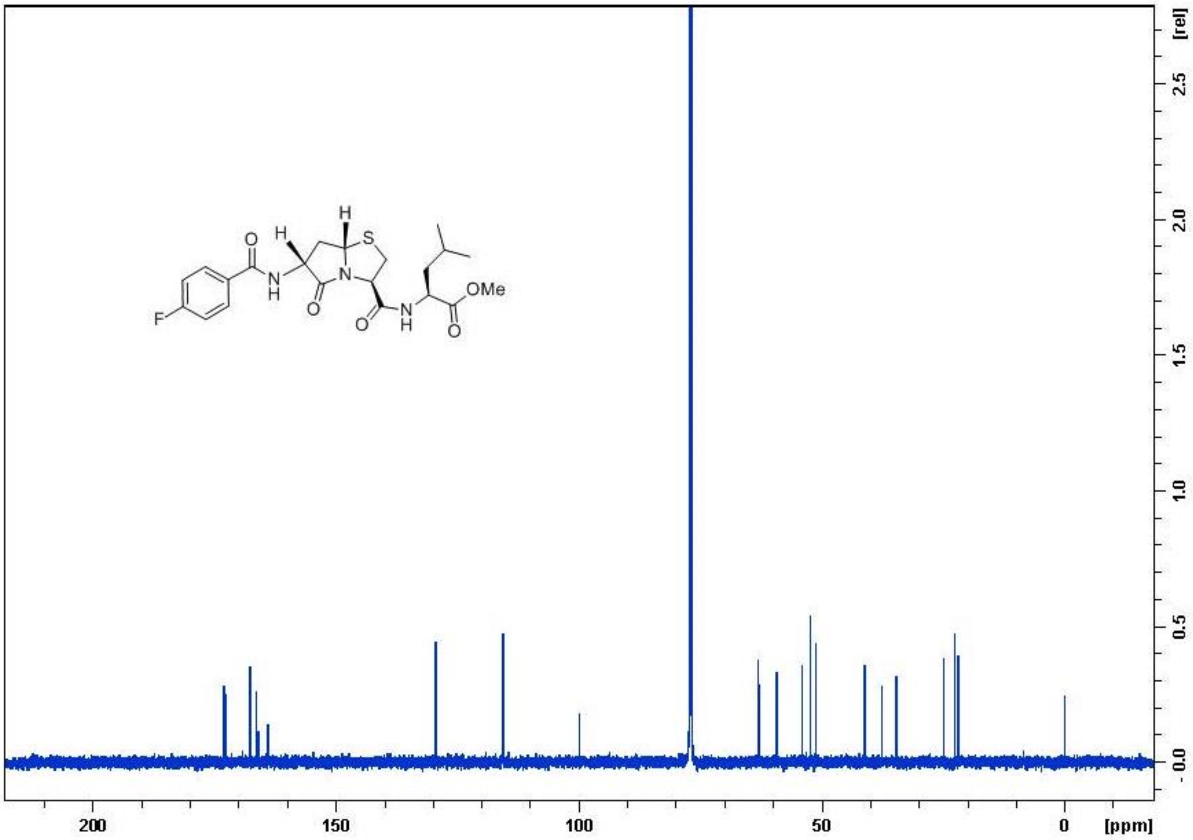

Figure S30. Carbon-13 NMR Spectrum of **β-34a** in $CDCl_3$

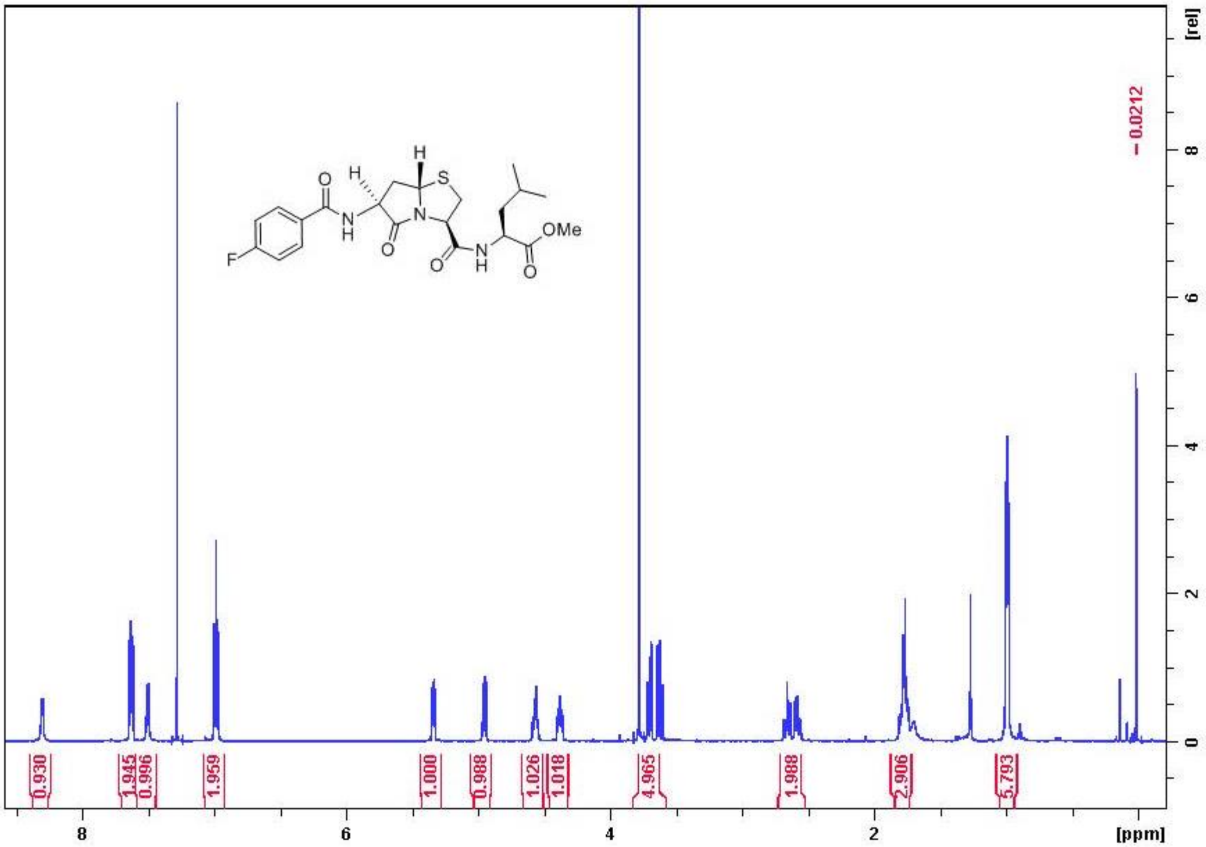

Figure S31. Proton NMR Spectrum of **α-34a** in $CDCl_3$

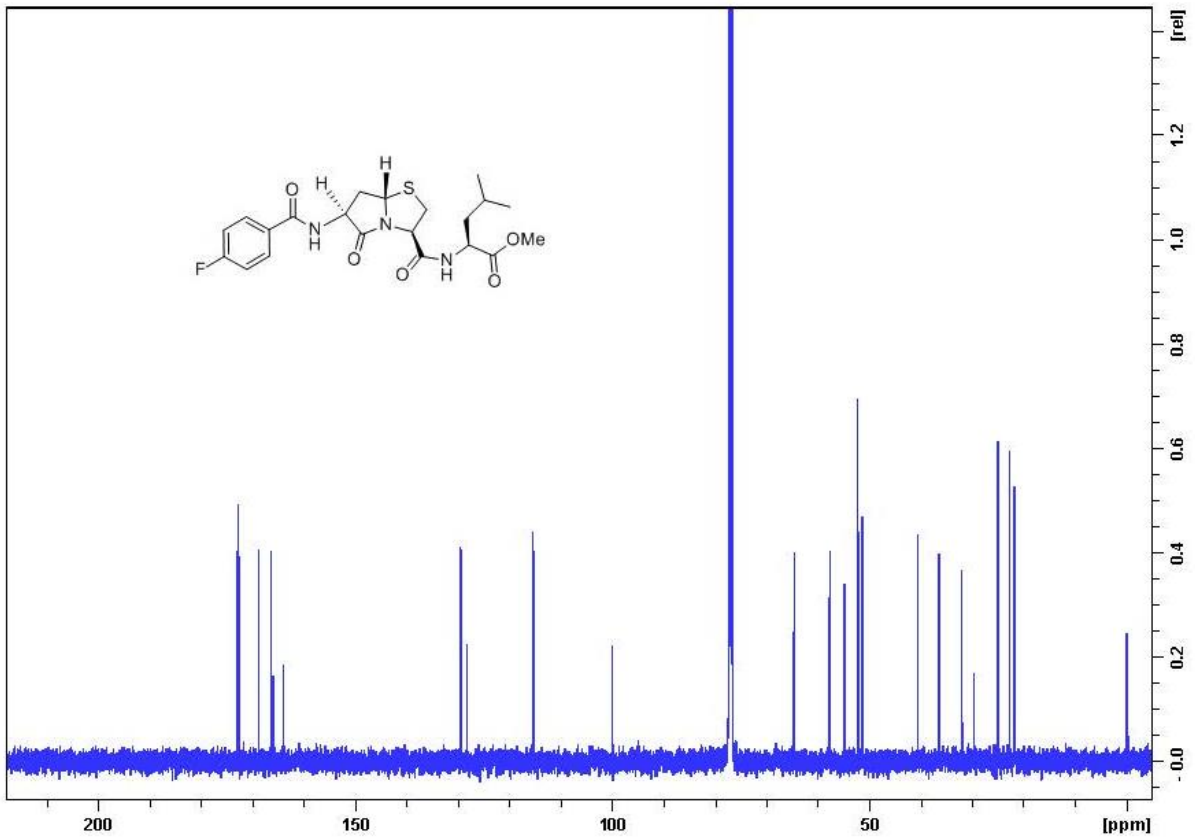

Figure S32. Carbon-13 NMR Spectrum of **α-34a** in $CDCl_3$

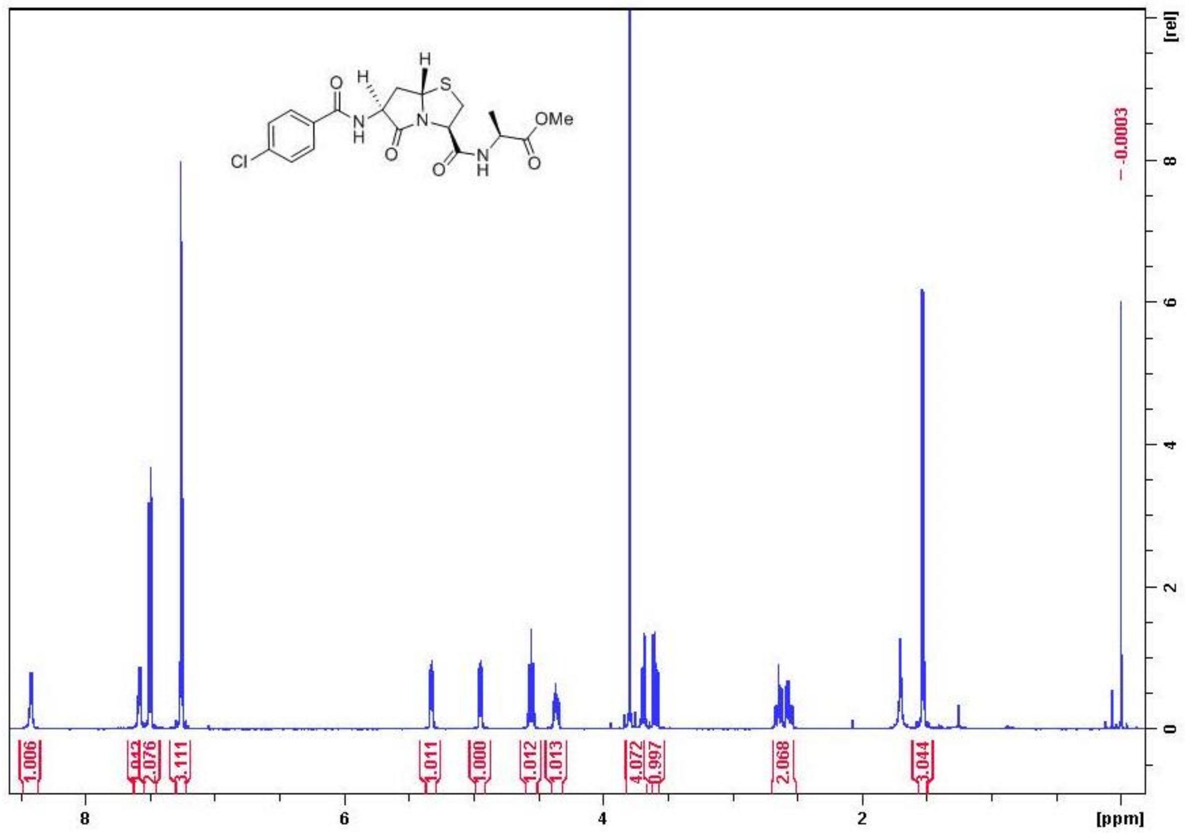

Figure S33. Proton NMR Spectrum of **α-35a** in $CDCl_3$

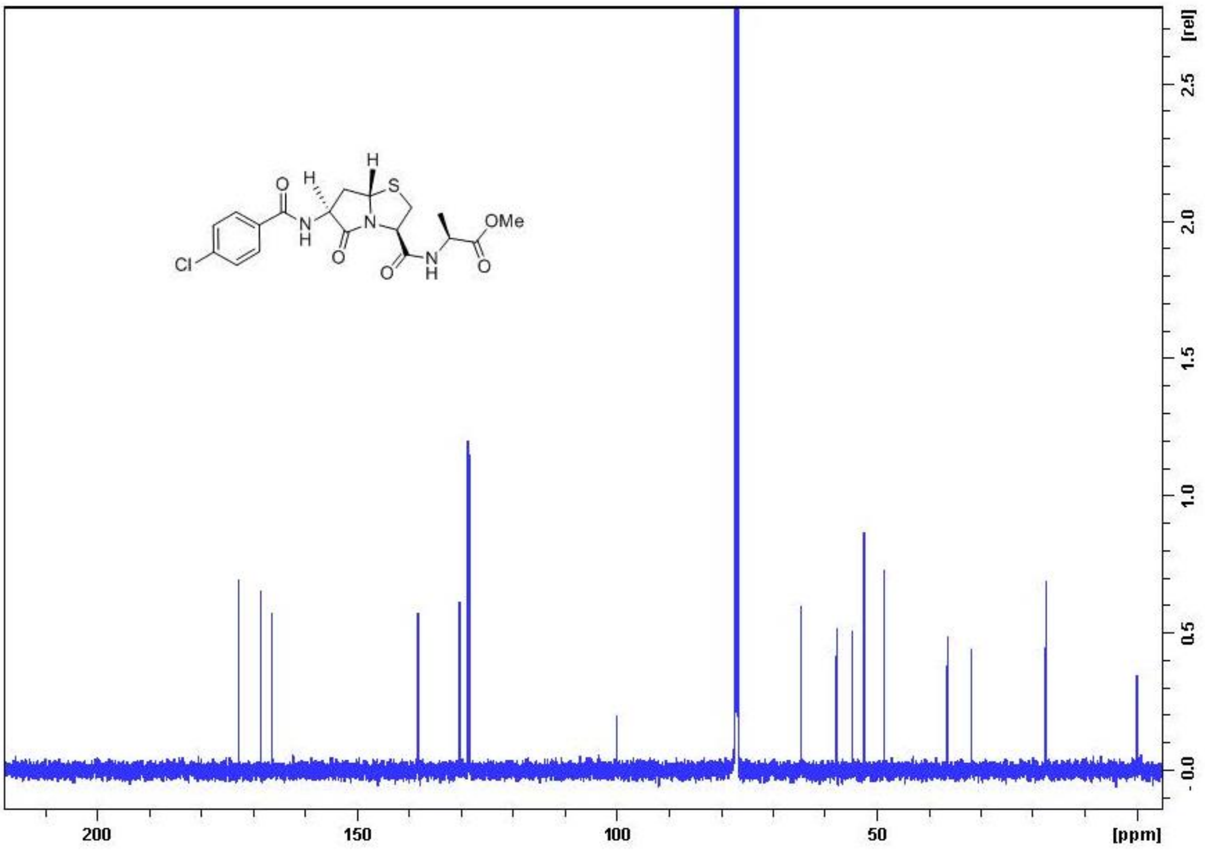

Figure S34. Carbon-13 NMR Spectrum of **α-35a** in $CDCl_3$

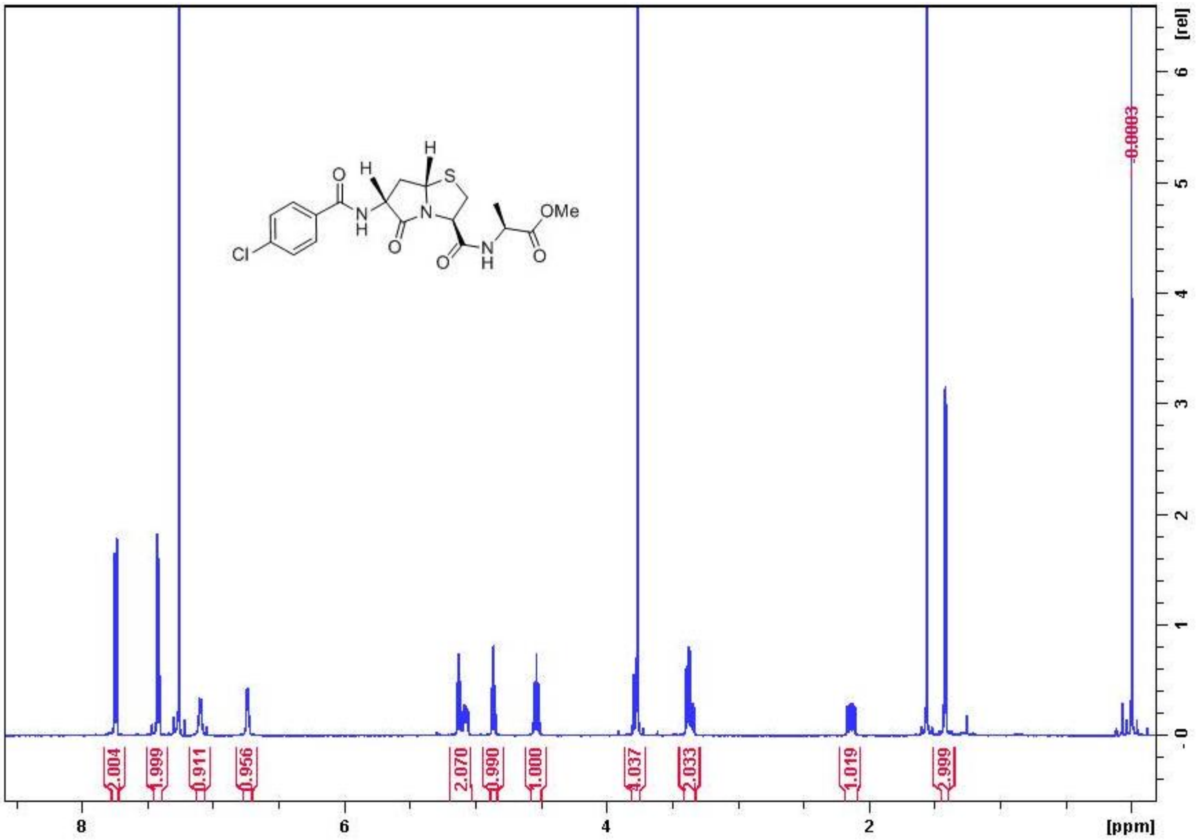

Figure S35. Proton NMR Spectrum of **β-35a** in $CDCl_3$

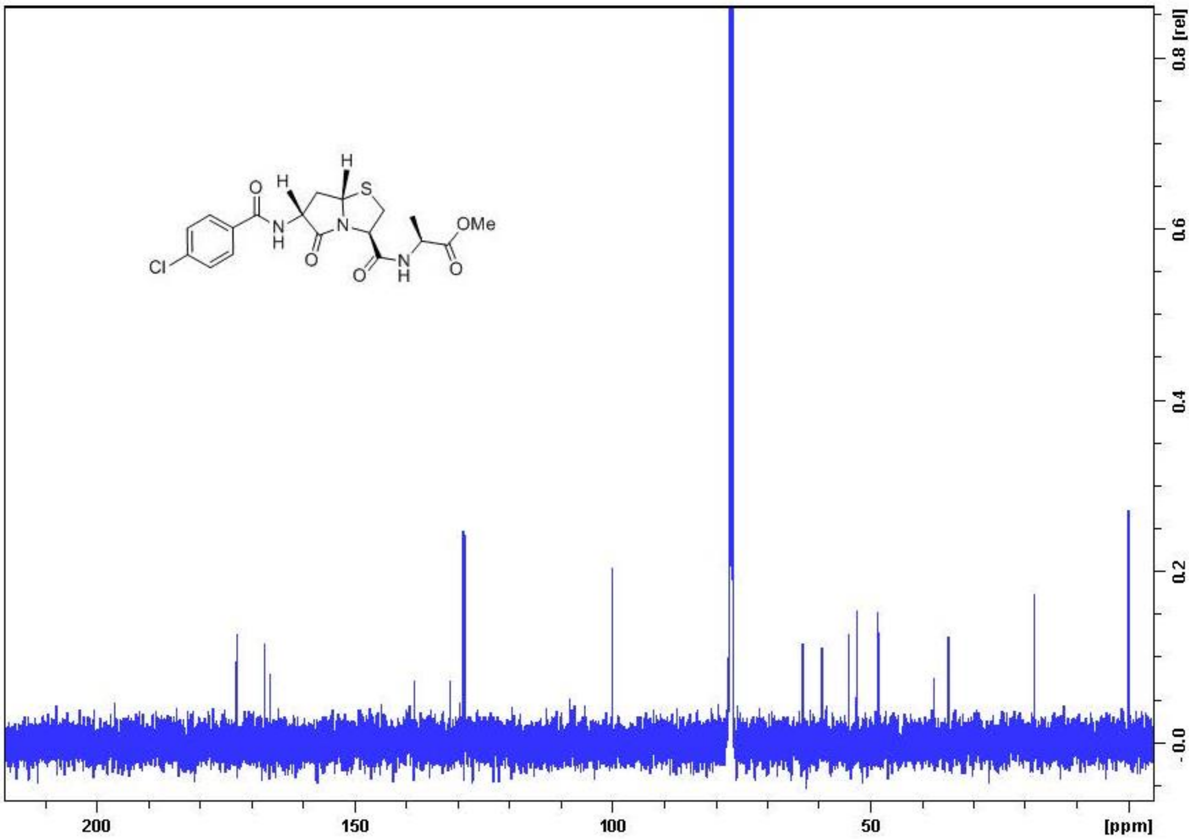

Figure S36. Carbon-13 NMR Spectrum of **β-35a** in $CDCl_3$

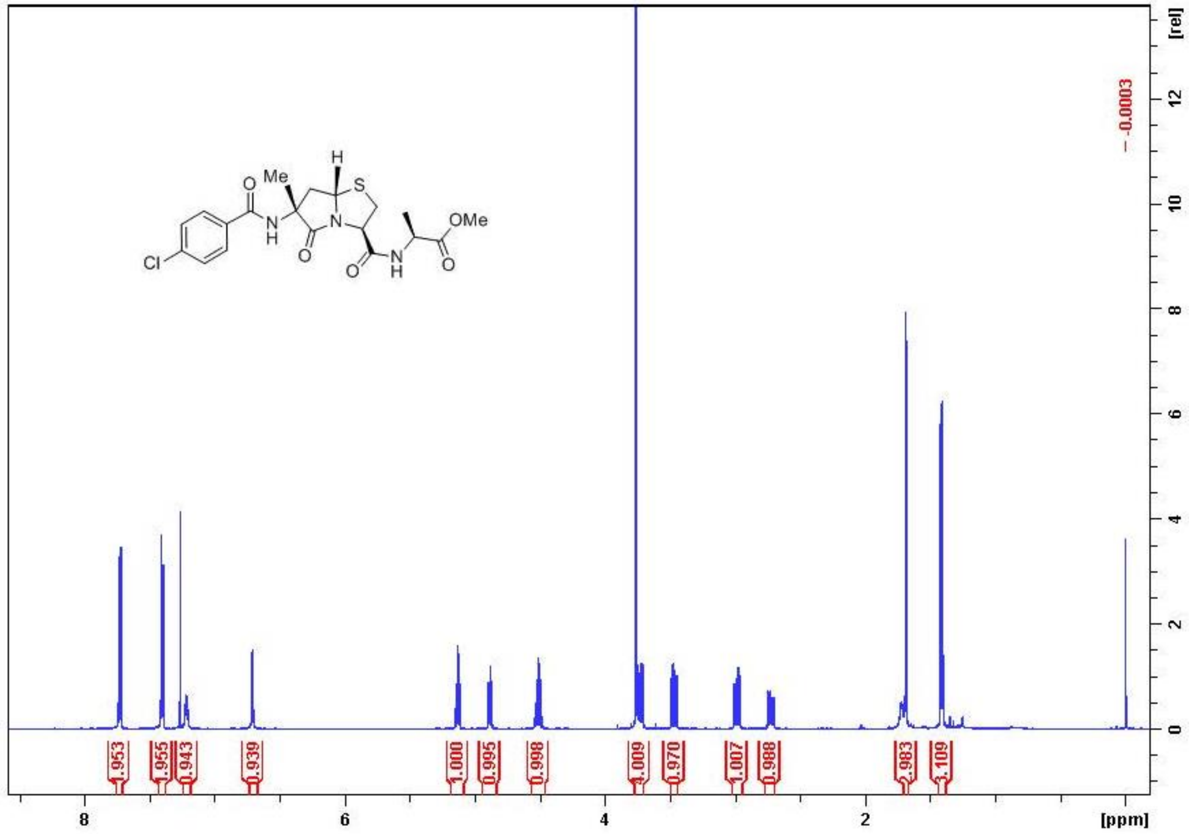

Figure S37. Proton NMR Spectrum of **β-35b** in $CDCl_3$

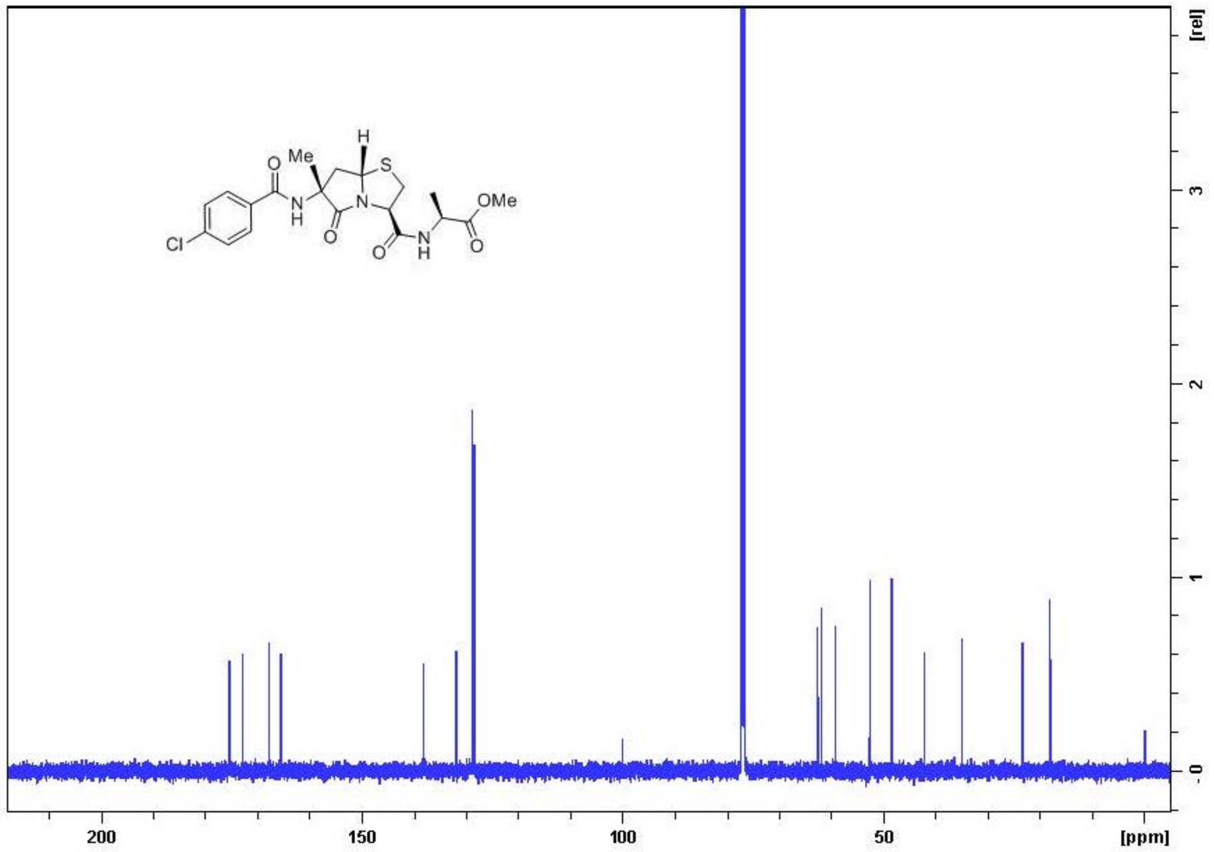

Figure S38. Carbon-13 NMR Spectrum of **β-35b** in $CDCl_3$

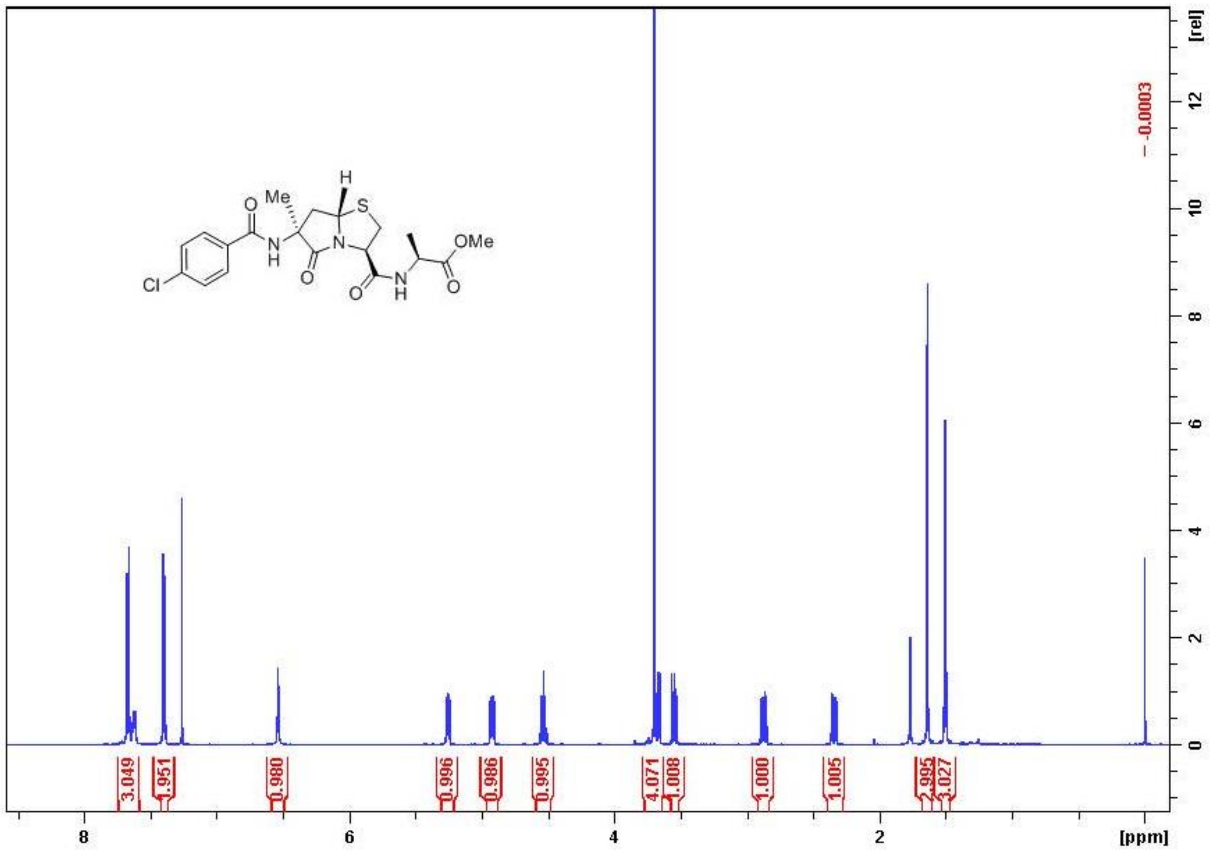

Figure S39. Proton NMR Spectrum of **α-35b** in $CDCl_3$

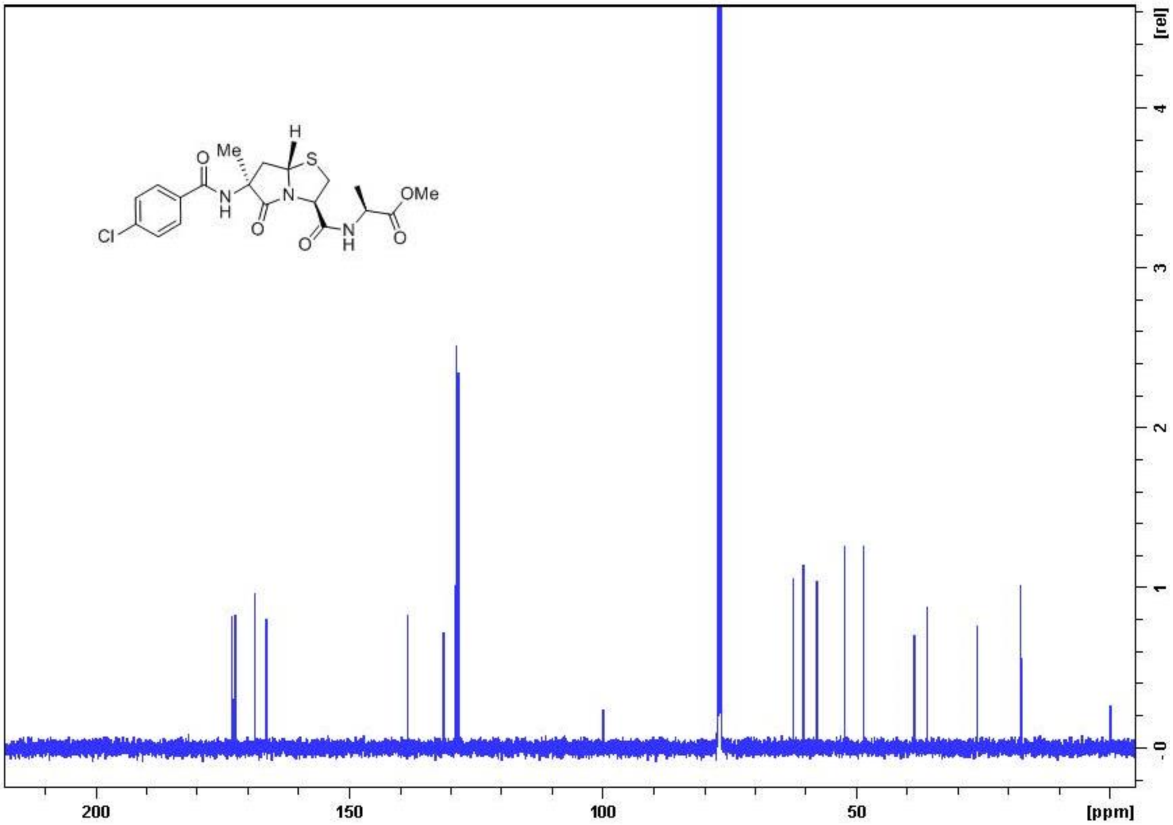

Figure S40. Carbon-13 NMR Spectrum of **α-35b** in $CDCl_3$

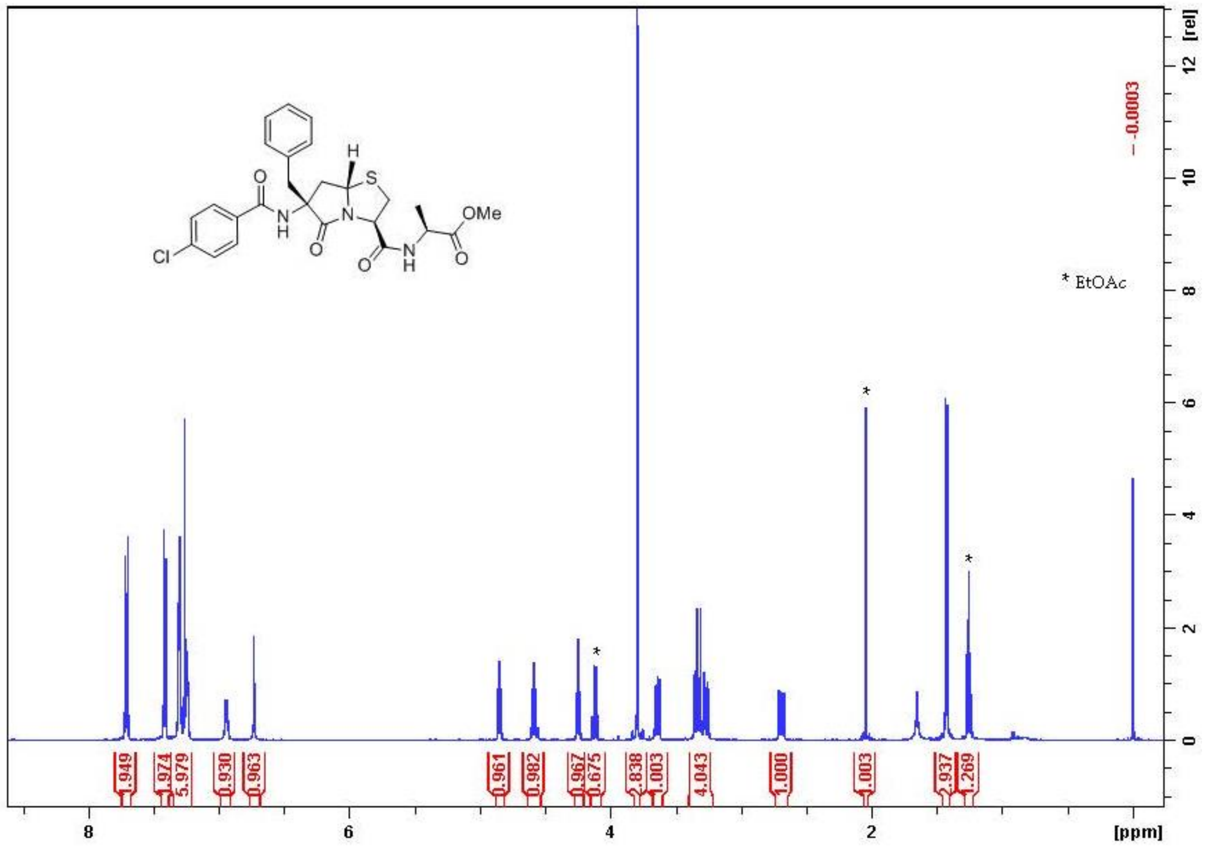

Figure S41. Proton NMR Spectrum of **β-35c** in $CDCl_3$

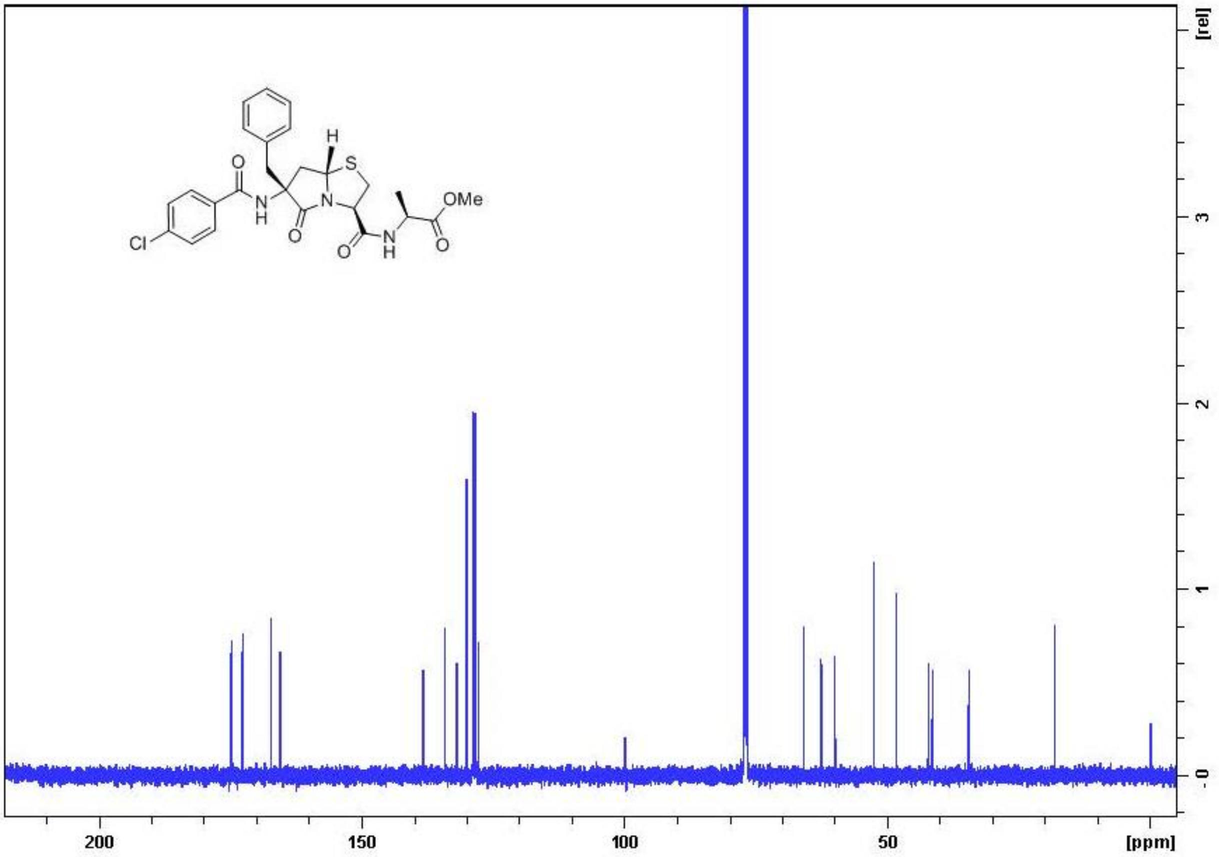

Figure S42. Carbon-13 NMR Spectrum of **β-35c** in $CDCl_3$

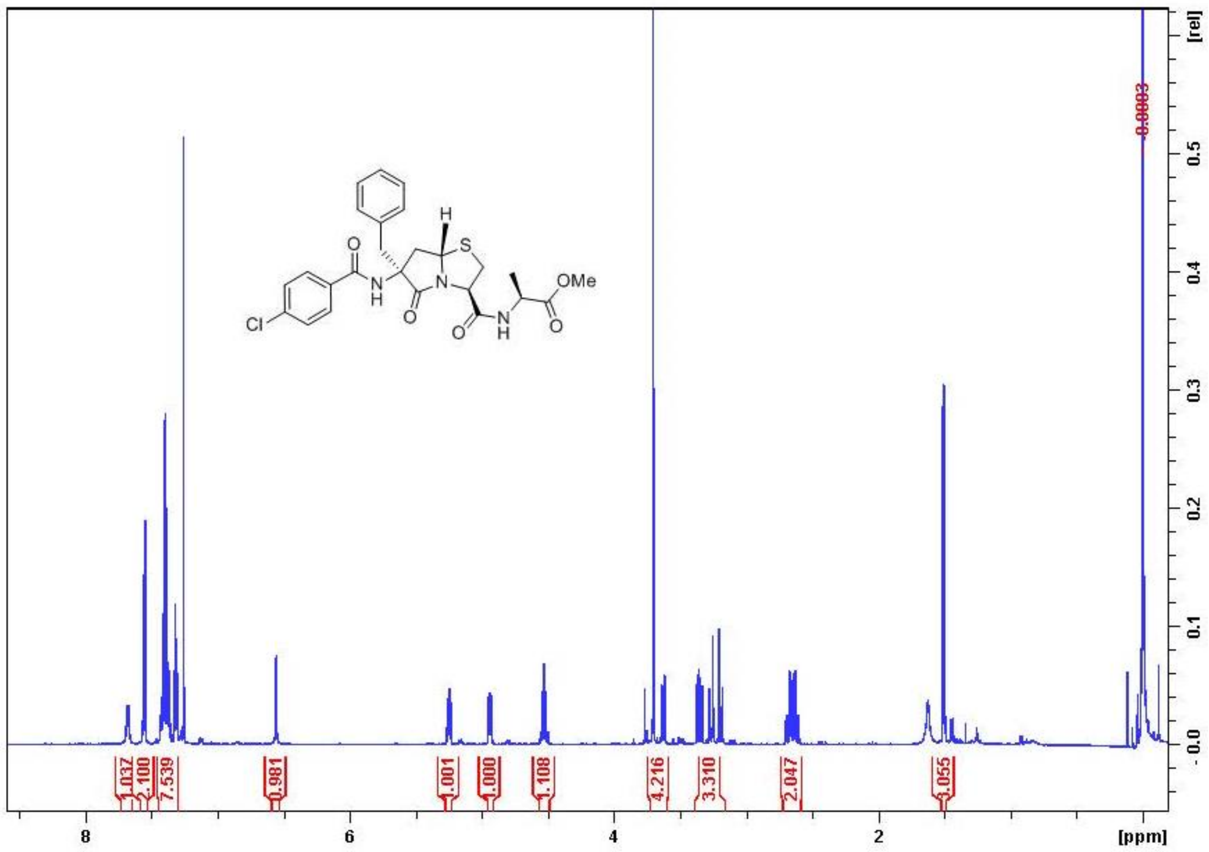

Figure S43. Proton NMR Spectrum of **α-35c** in $CDCl_3$

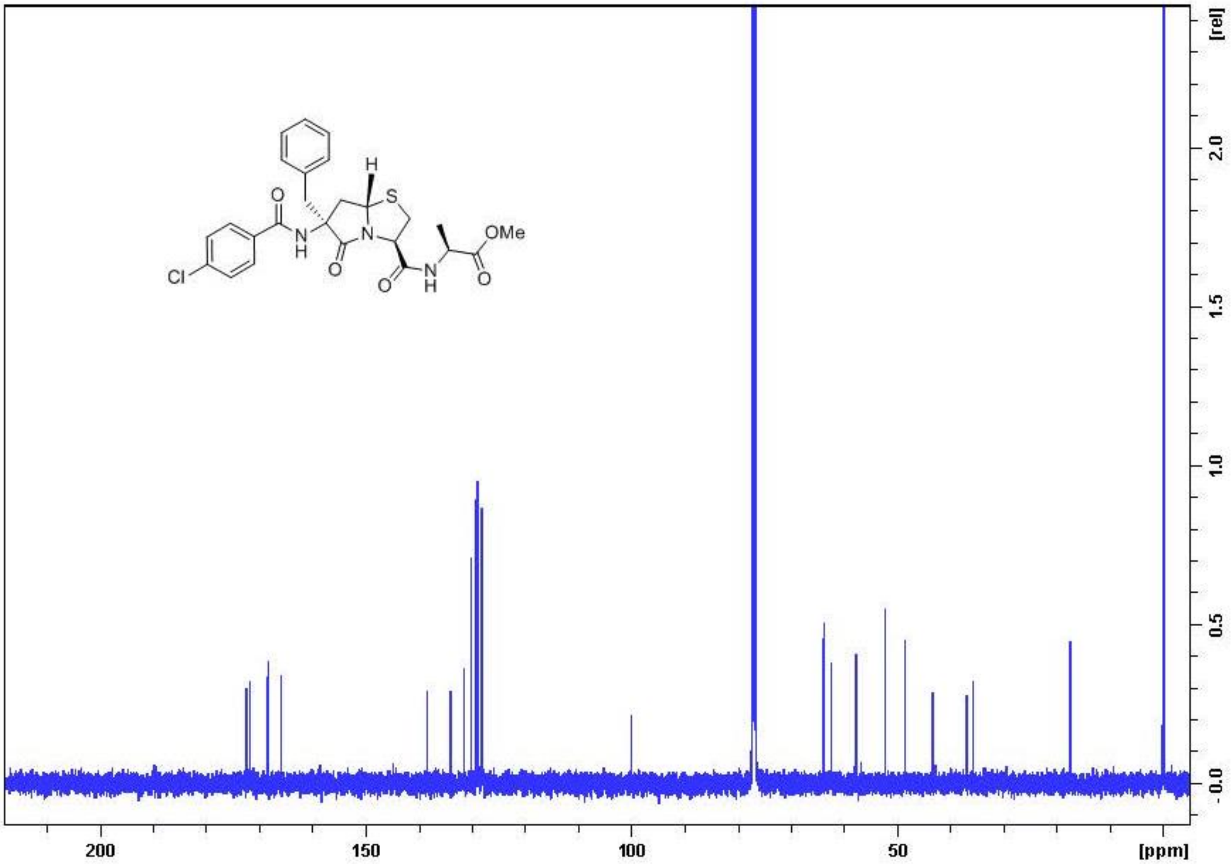

Figure S44. Carbon-13 NMR Spectrum of **α-35c** in $CDCl_3$

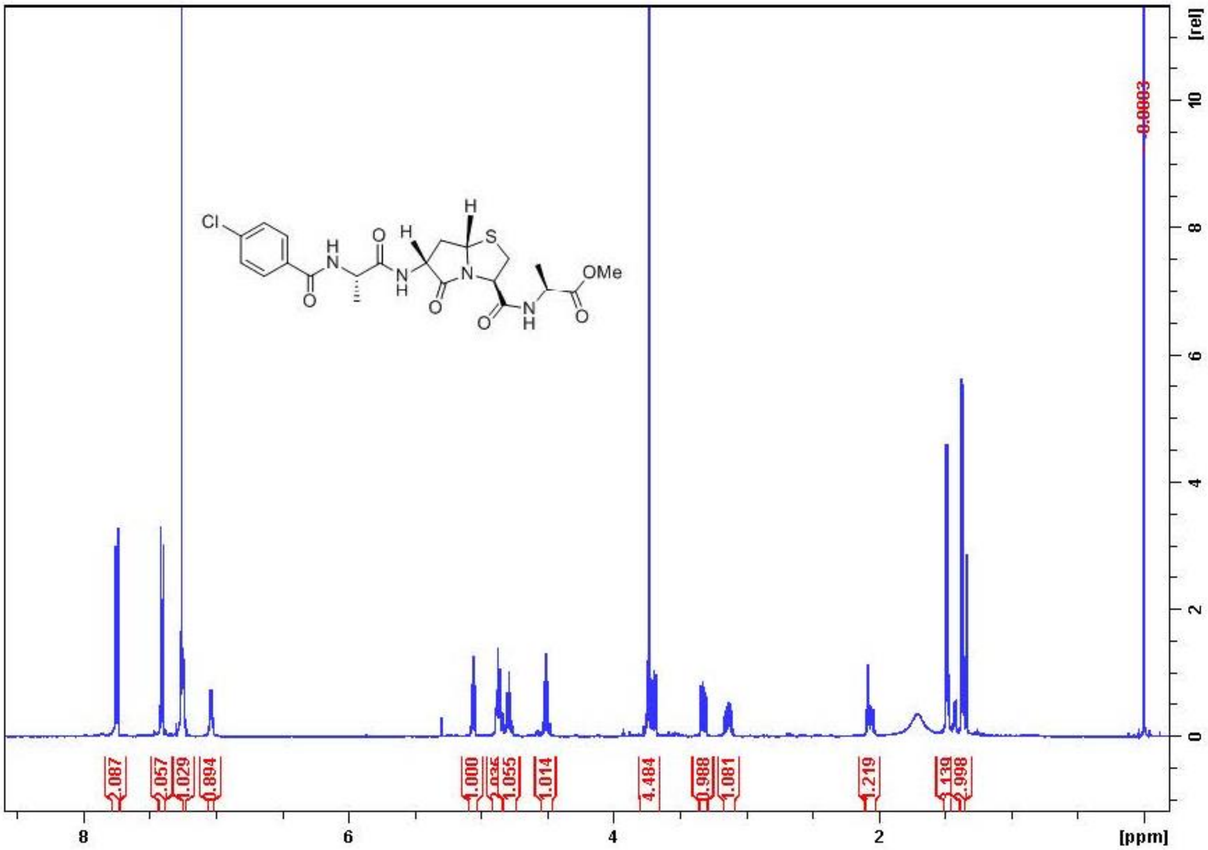

Figure S45. Proton NMR Spectrum of **β-36a** in $CDCl_3$

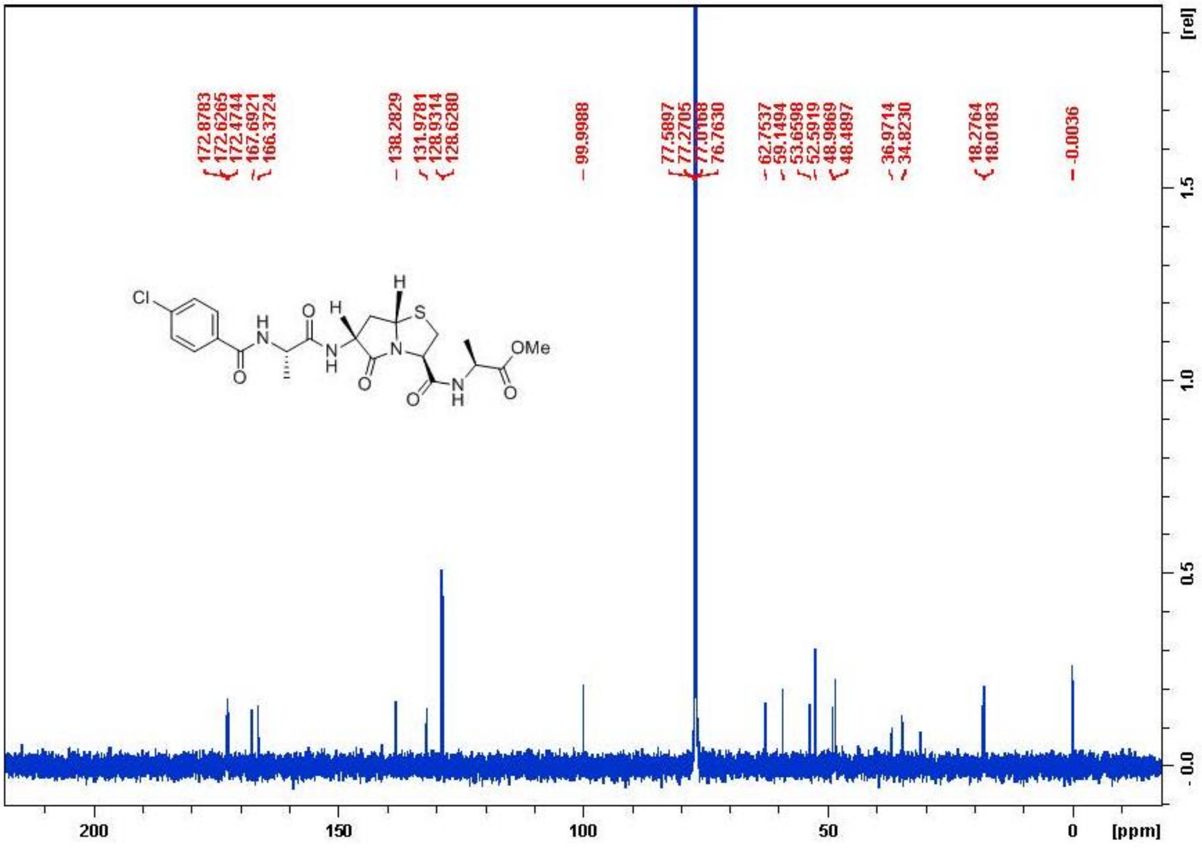

Figure S46. Carbon-13 NMR Spectrum of **β-36a** in $CDCl_3$

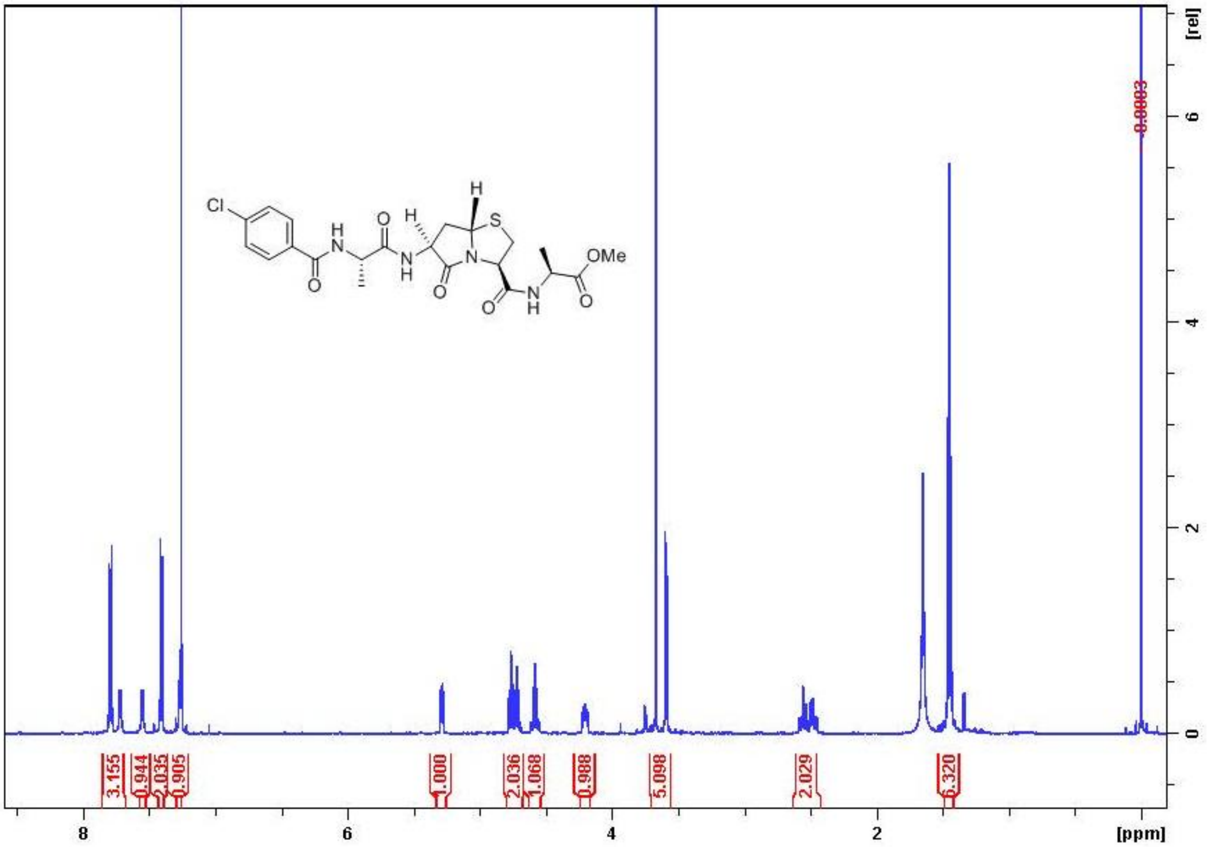

Figure S47. Proton NMR Spectrum of **α-36a** in $CDCl_3$

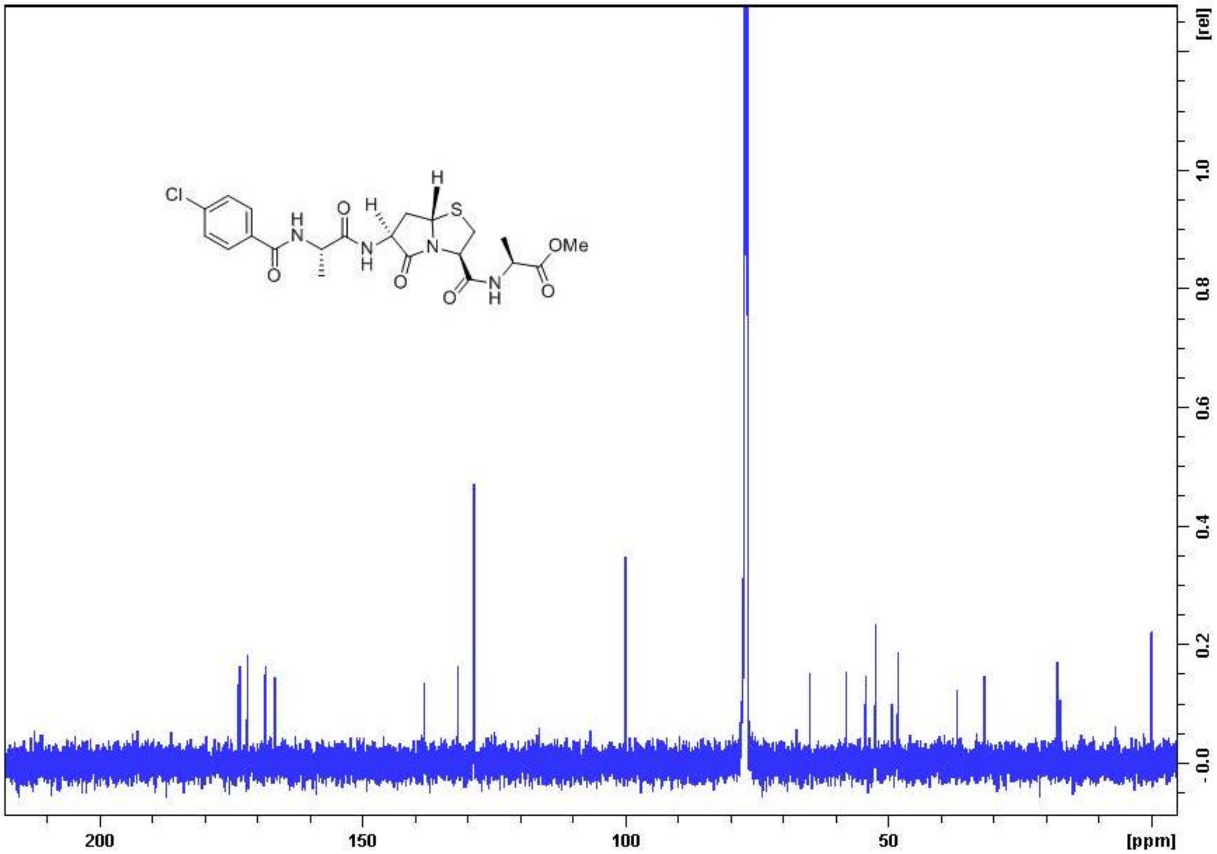

Figure S48. Carbon-13 NMR Spectrum of **α-36a** in $CDCl_3$

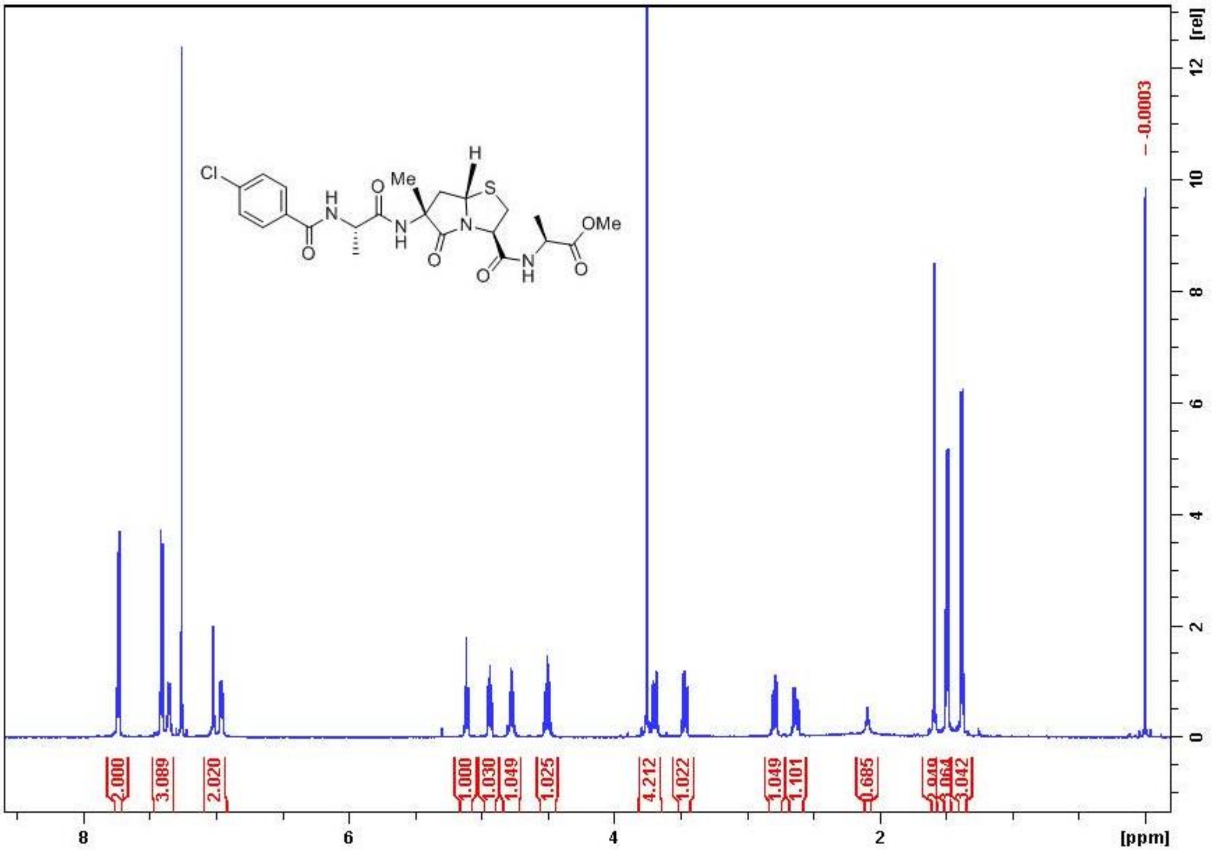

Figure S49. Proton NMR Spectrum of **β-36b** in $CDCl_3$

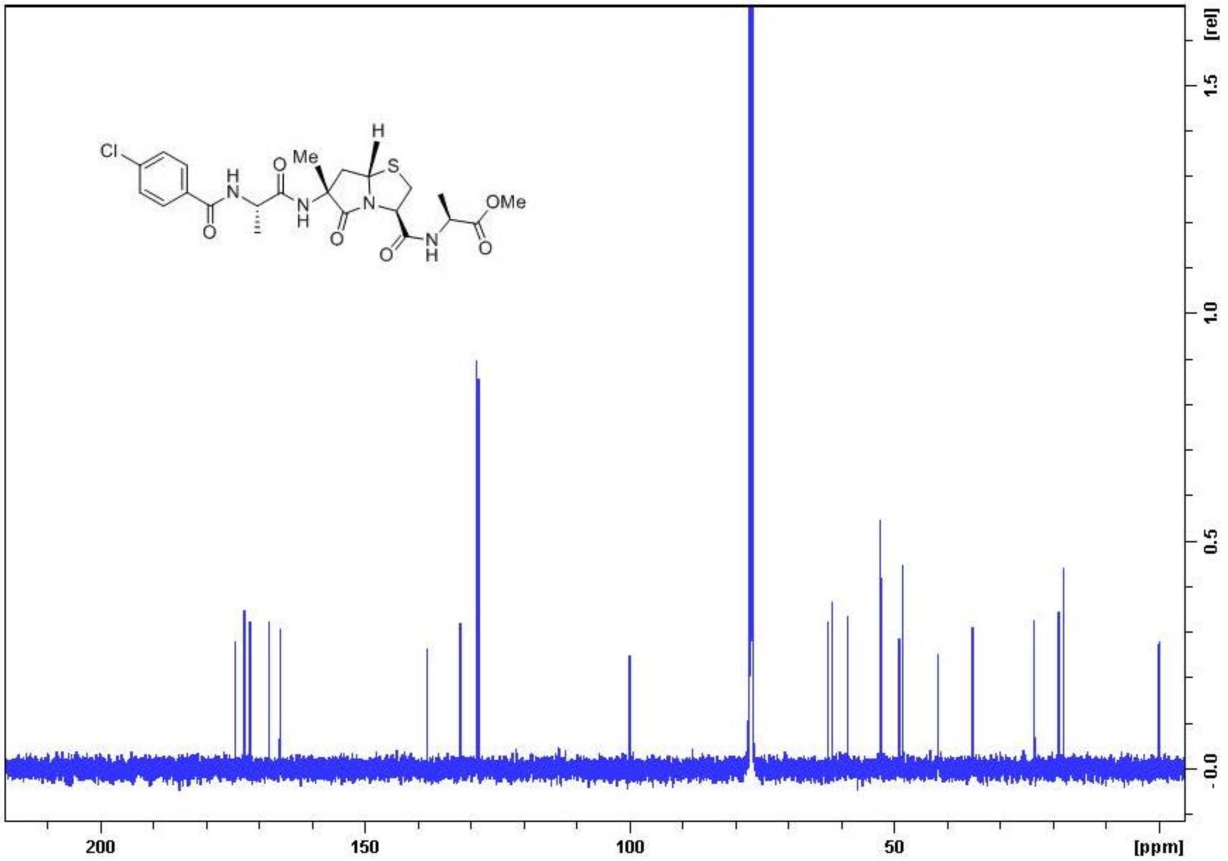

Figure S50. Carbon-13 NMR Spectrum of **β-36b** in $CDCl_3$

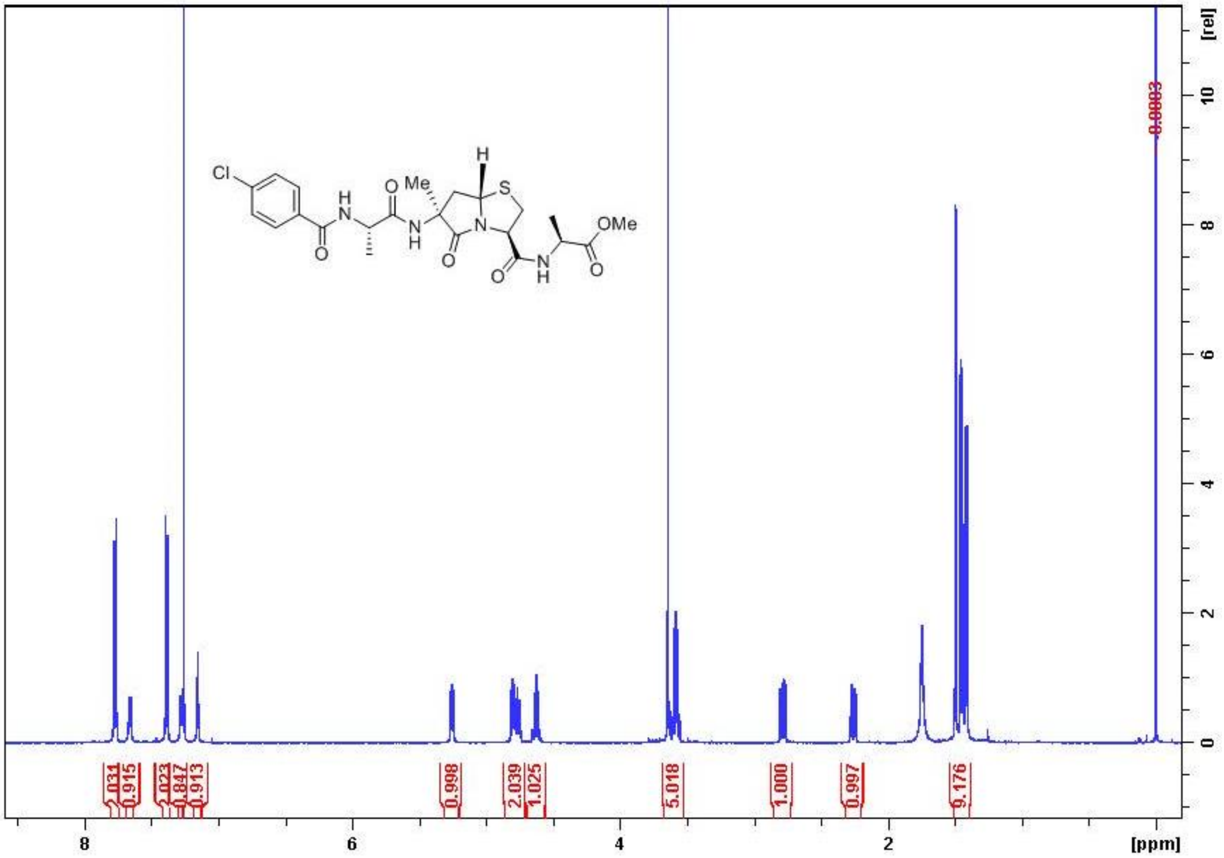

Figure S51. Proton NMR Spectrum of **α-36b** in $CDCl_3$

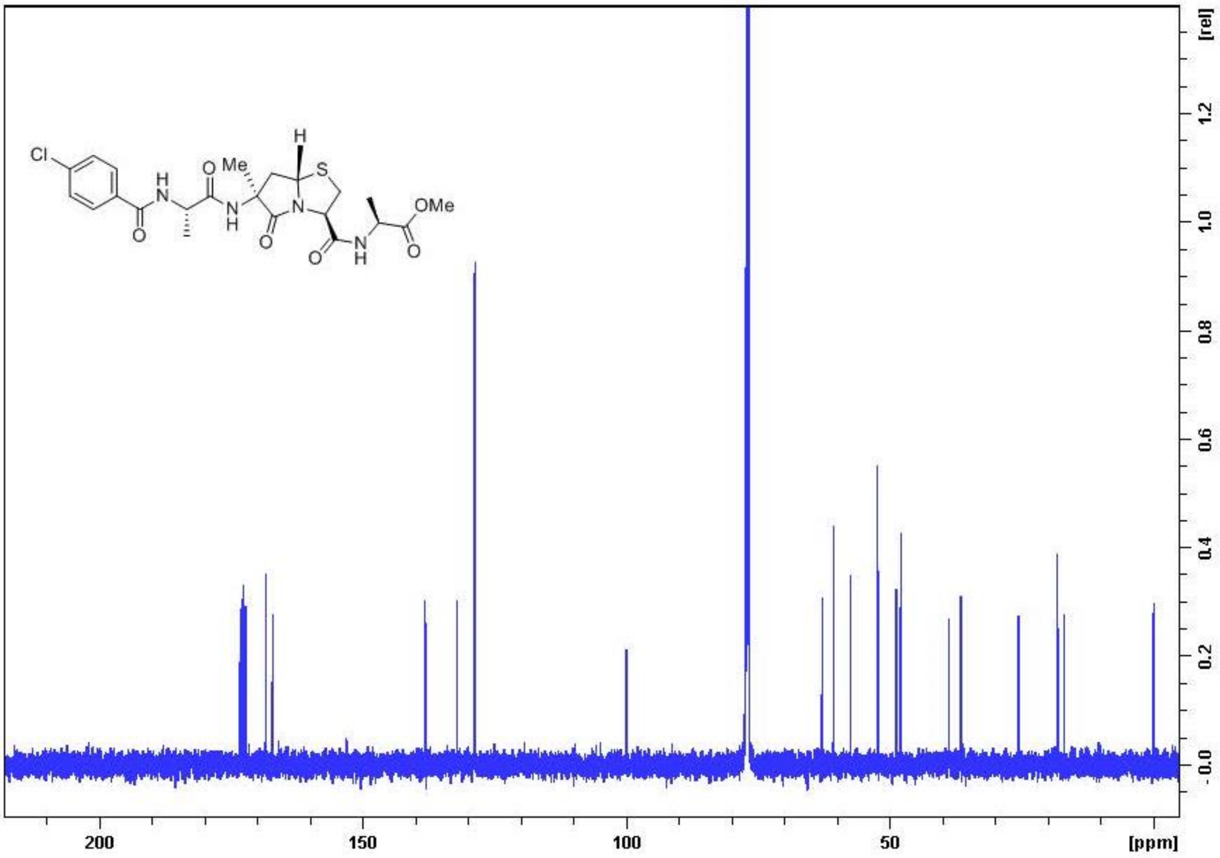

Figure S52. Carbon-13 NMR Spectrum of **α-36b** in $CDCl_3$

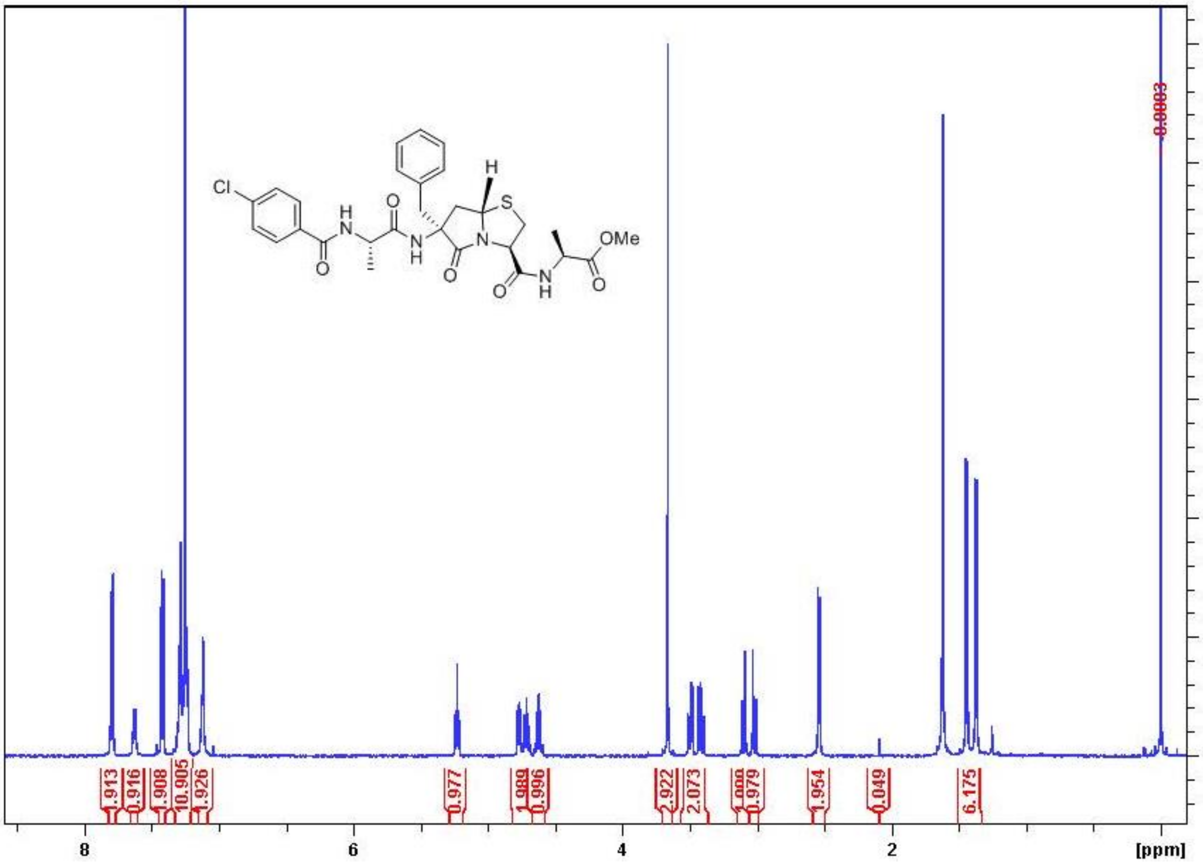

Figure S53. Proton NMR Spectrum of **α-36c** in $CDCl_3$

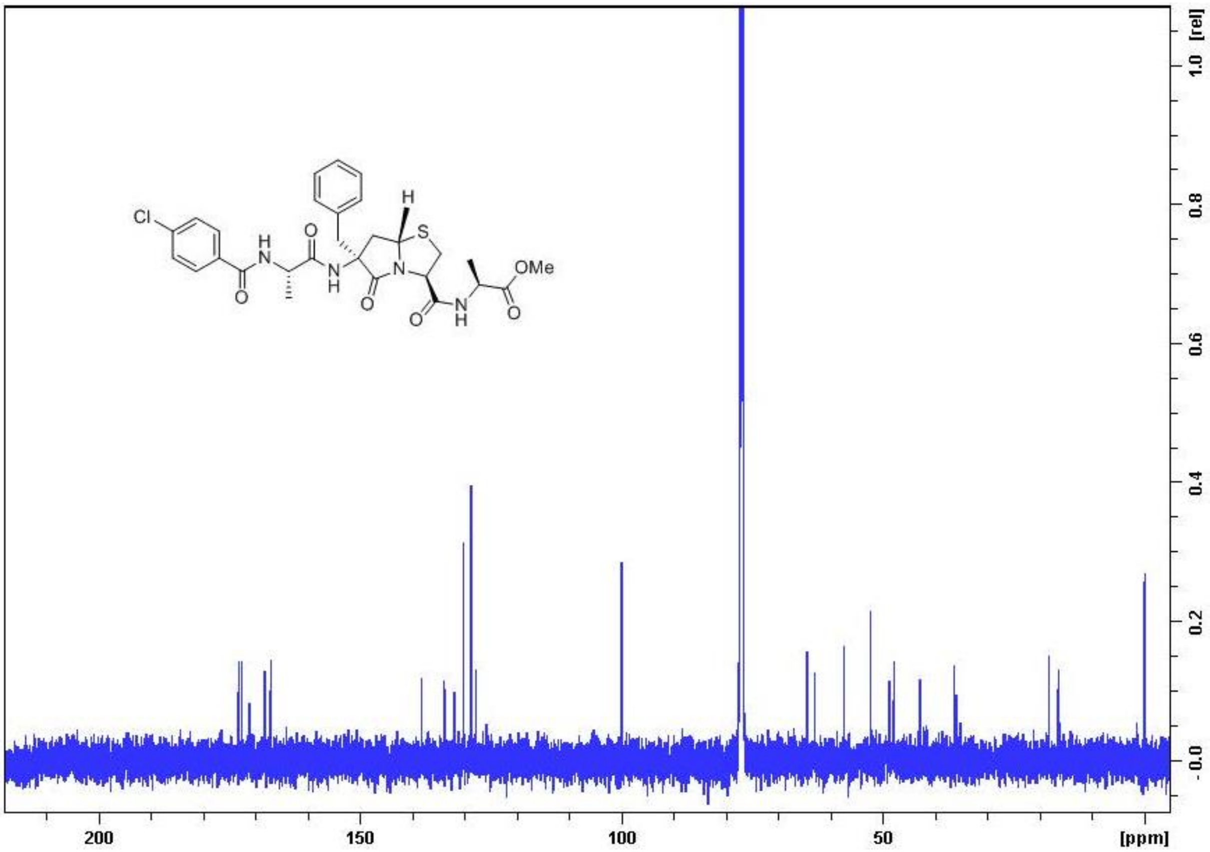

Figure S54. Carbon-13 NMR Spectrum of **α-36c** in $CDCl_3$

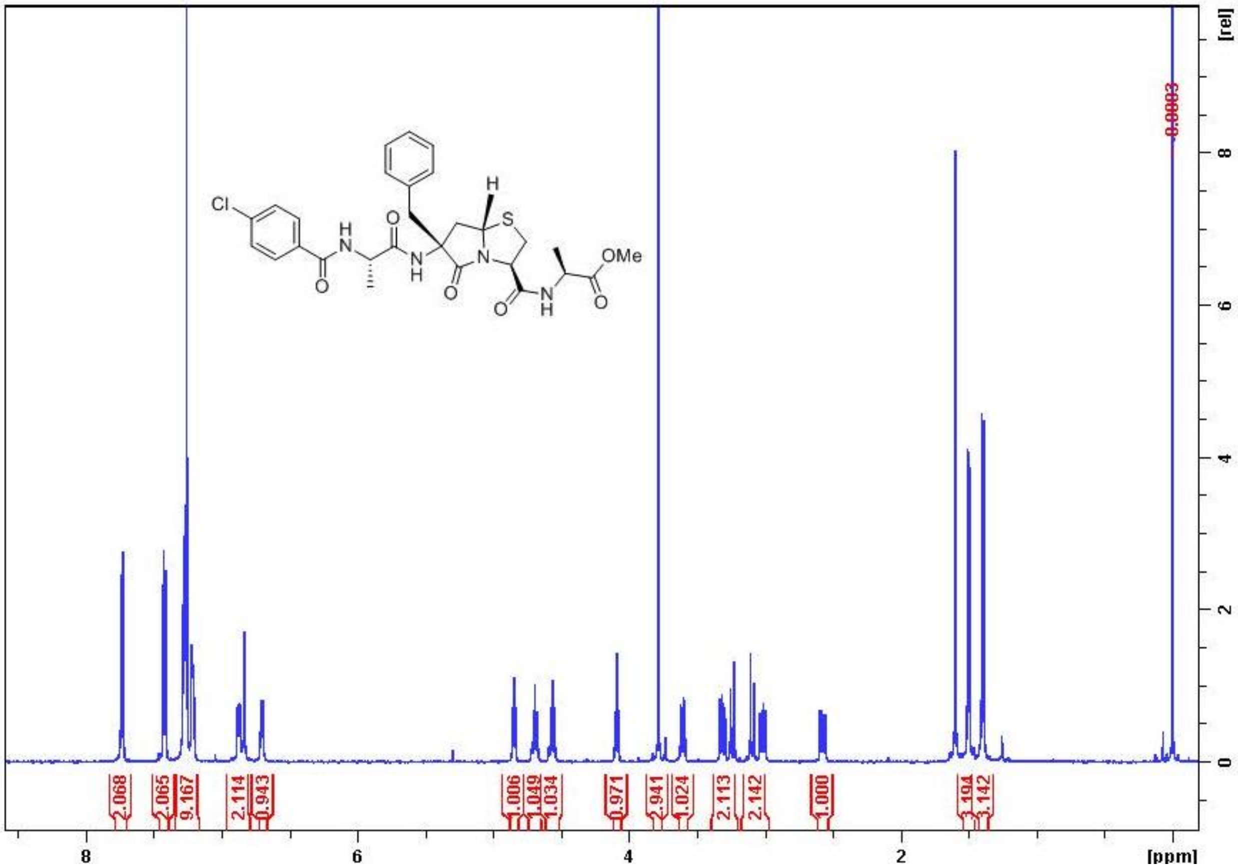

Figure S55. Proton NMR Spectrum of **β-36c** in $CDCl_3$

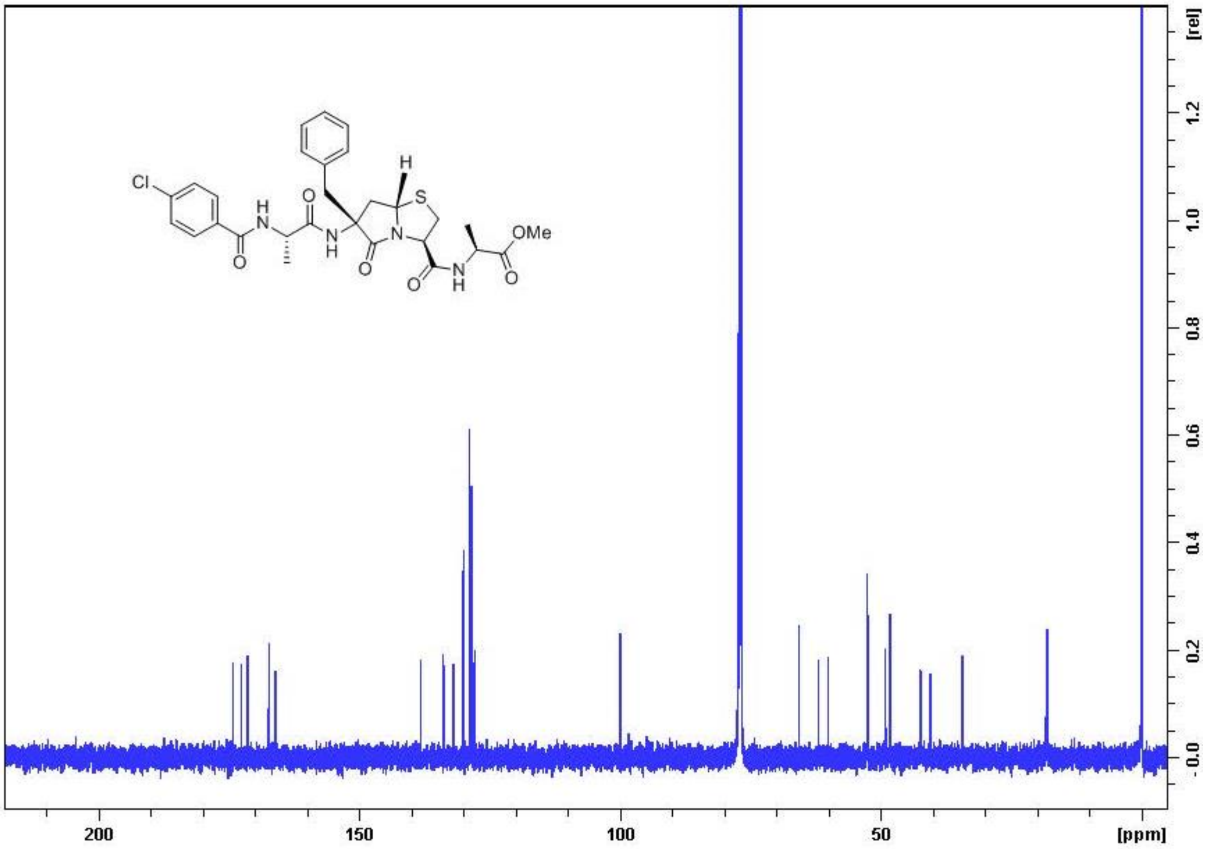

Figure S56. Carbon-13 NMR Spectrum of **β-36c** in $CDCl_3$

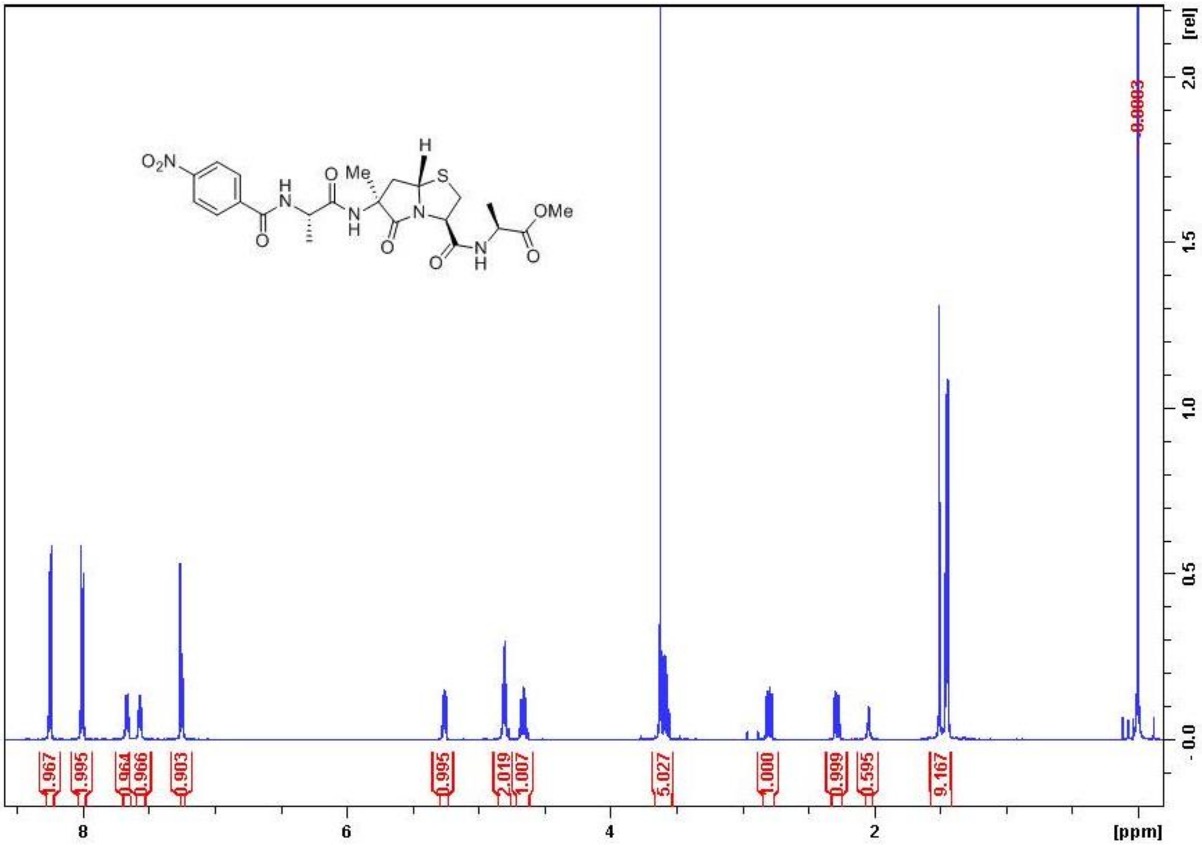

Figure S57. Proton NMR Spectrum of **α-30b** in $CDCl_3$

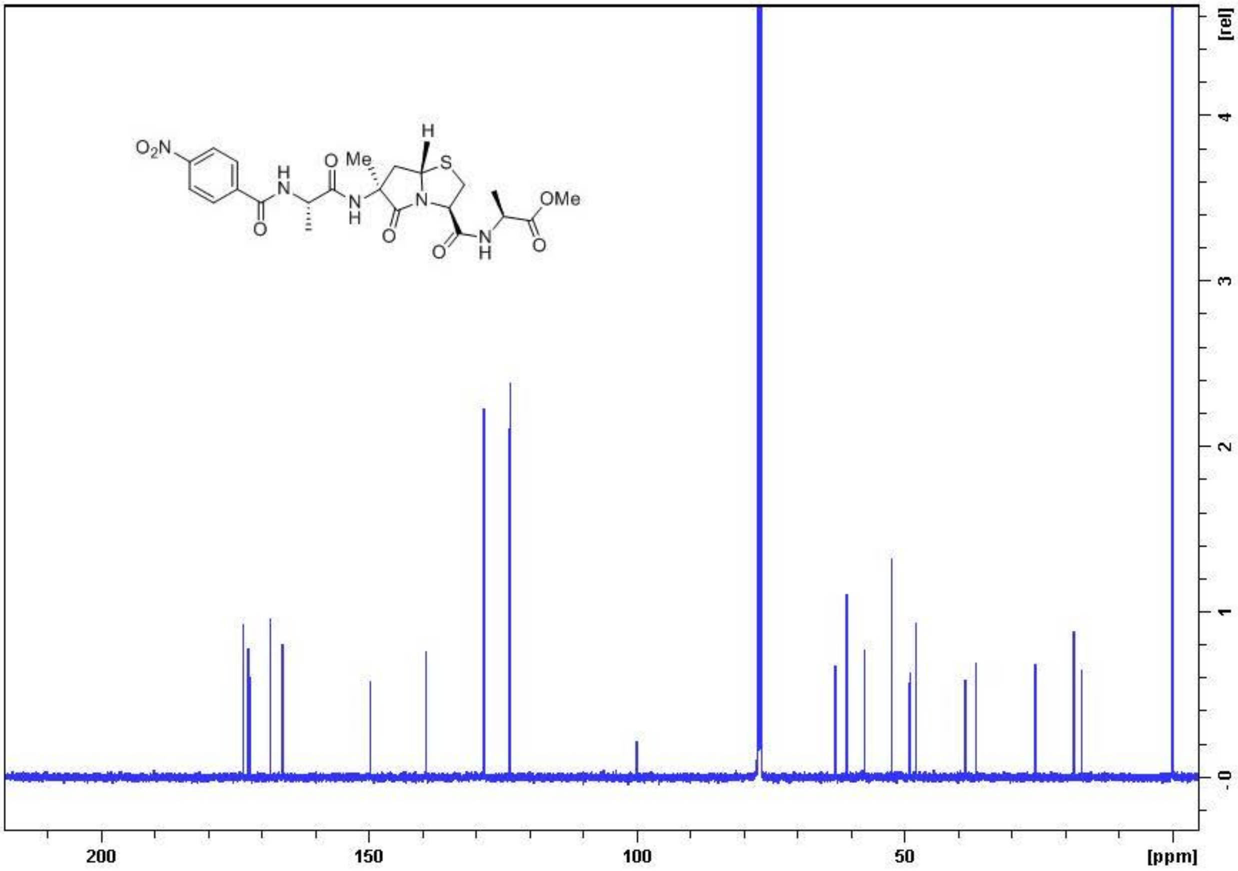

Figure S58. Carbon-13 NMR Spectrum of **α-30b** in $CDCl_3$

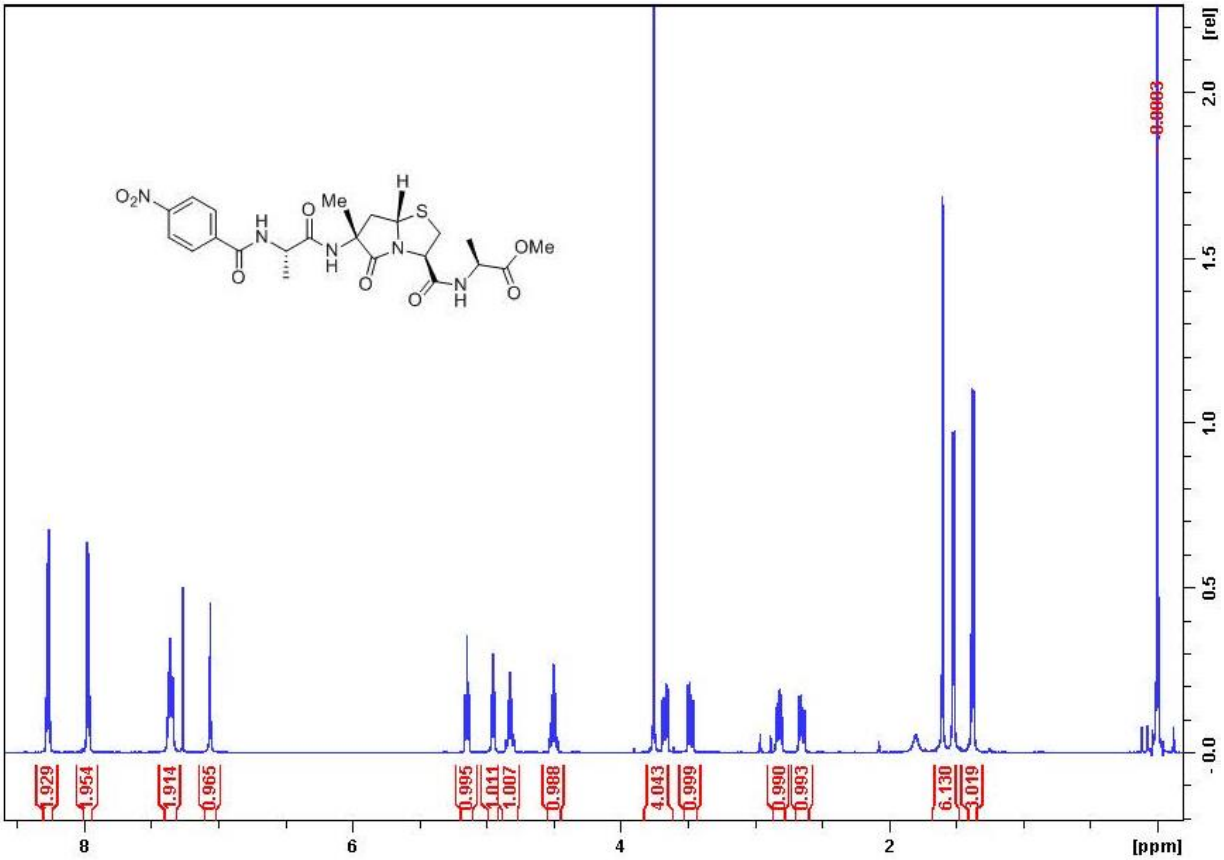

Figure S59. Proton NMR Spectrum of **β-30b** in $CDCl_3$

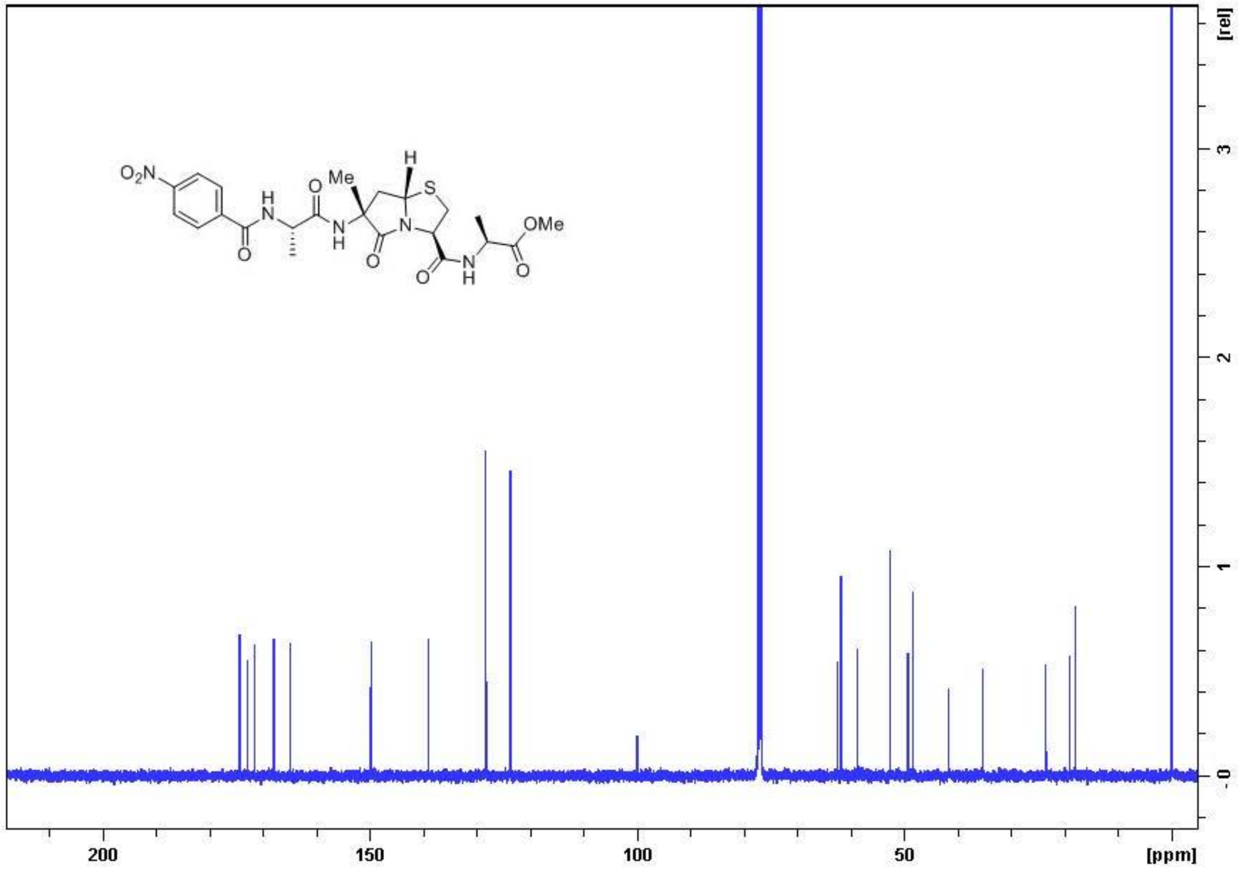

Figure S60. Carbon-13 NMR Spectrum of **β-30b** in $CDCl_3$

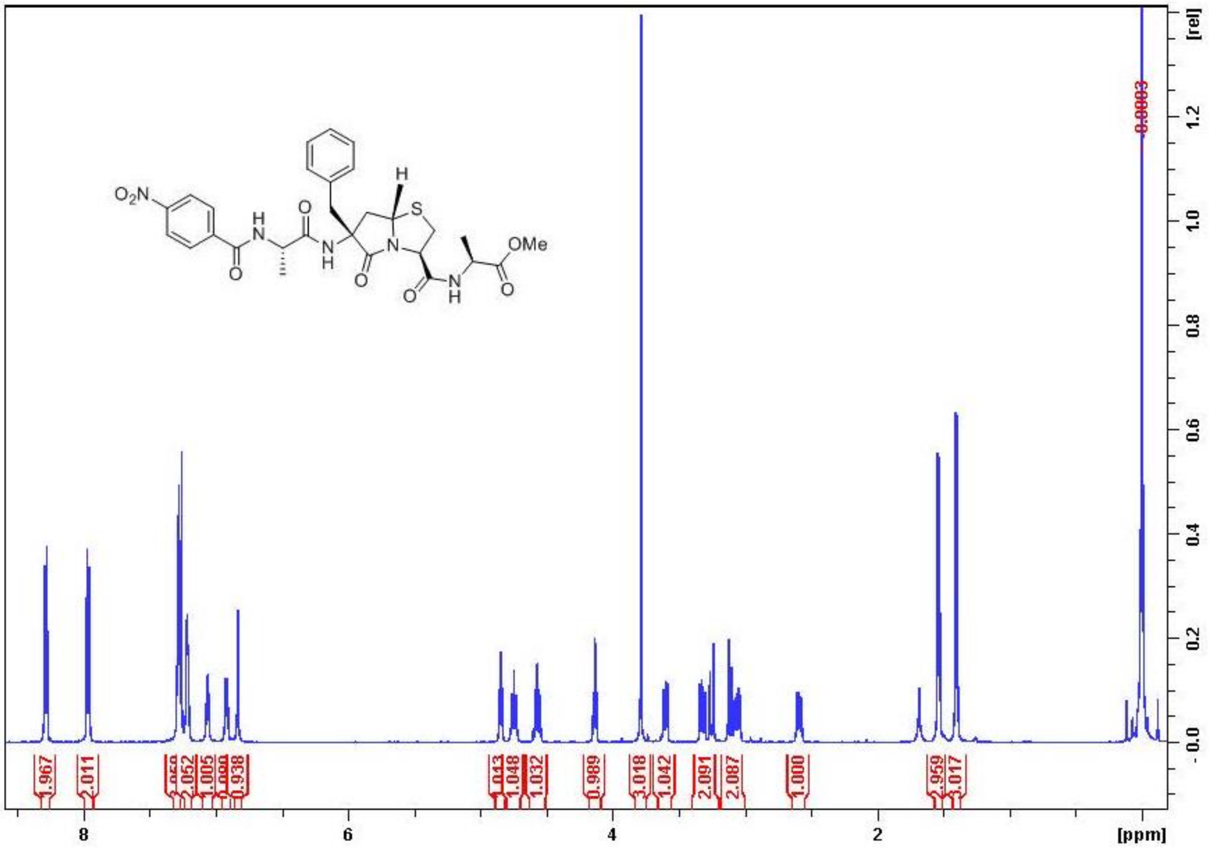

Figure S61. Proton NMR Spectrum of **β-30c** in $CDCl_3$

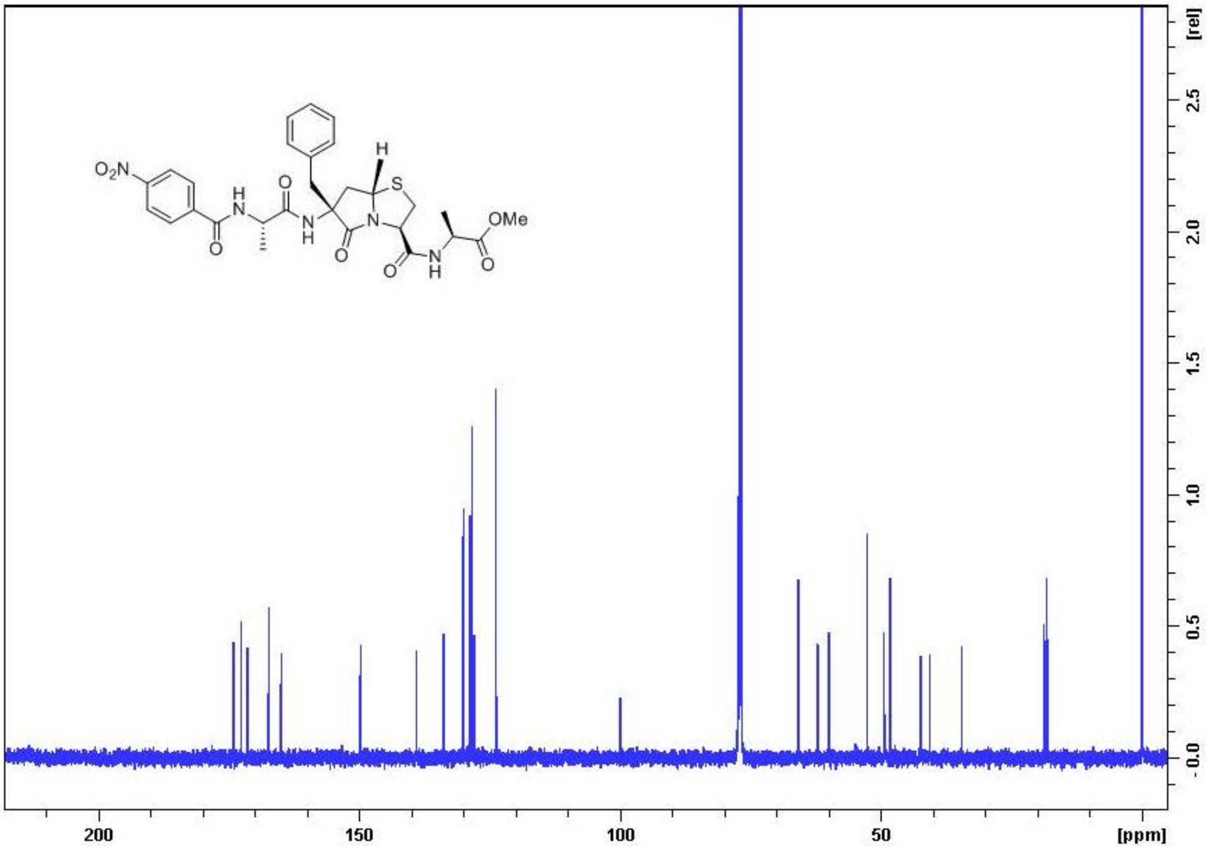

Figure S62. Carbon-13 NMR Spectrum of **β-30c** in $CDCl_3$

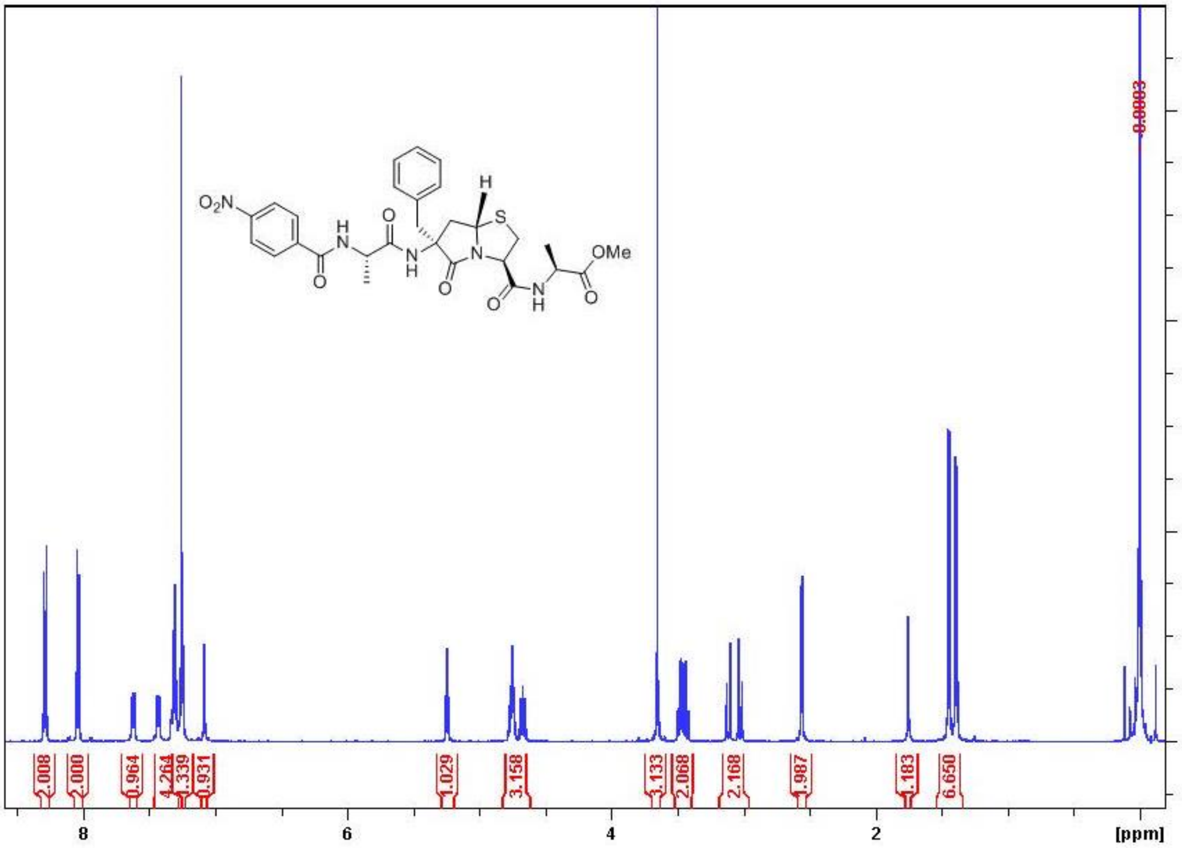

Figure S63. Proton NMR Spectrum of **α-30c** in $CDCl_3$

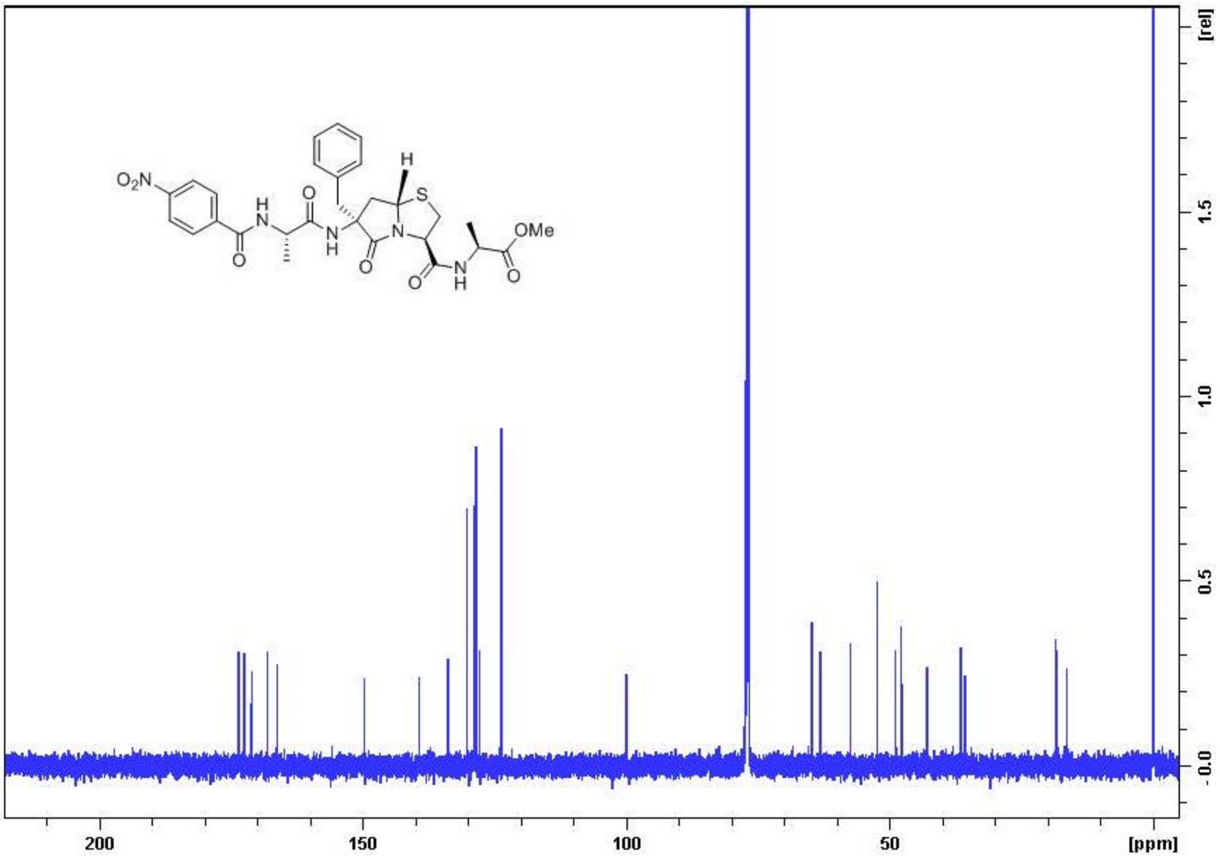

Figure S64. Carbon-13 NMR Spectrum of **α-30c** in $CDCl_3$

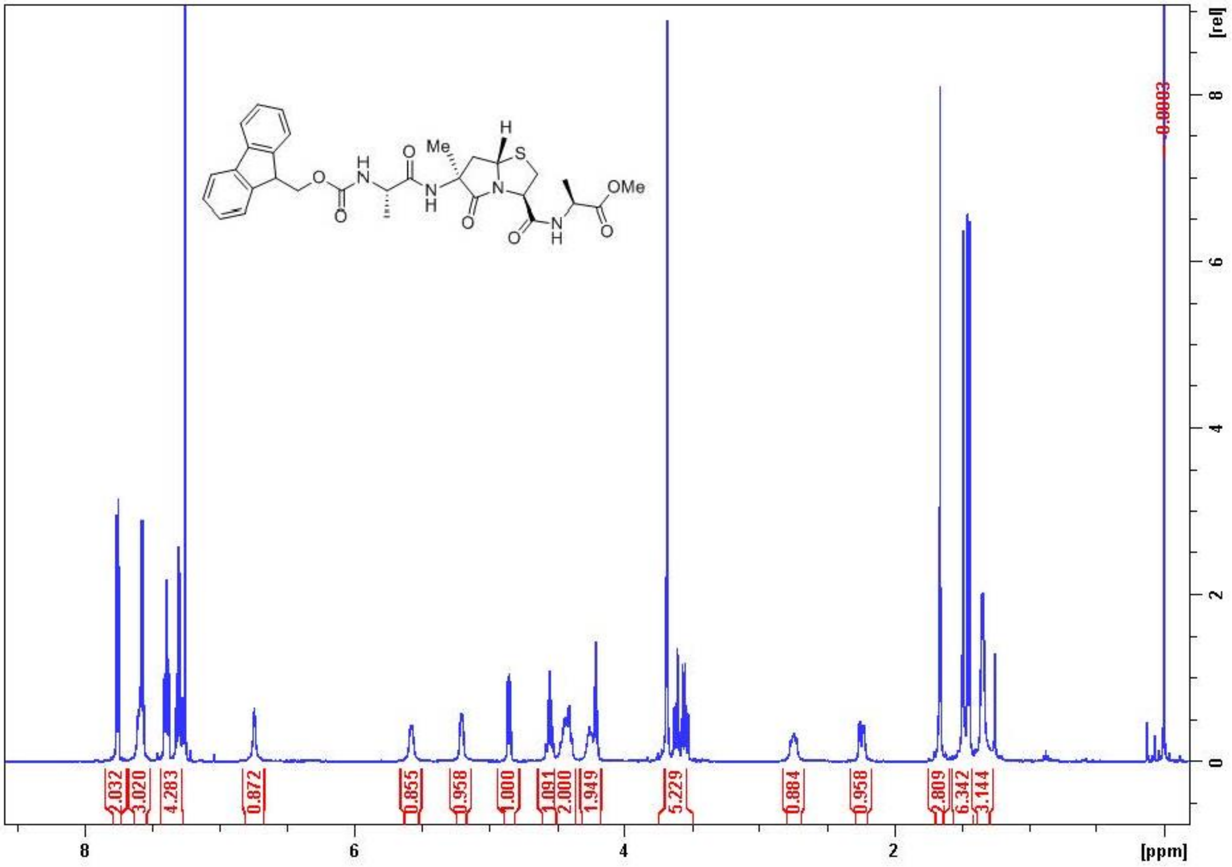

Figure S65. Proton NMR Spectrum of **α-37b** in $CDCl_3$

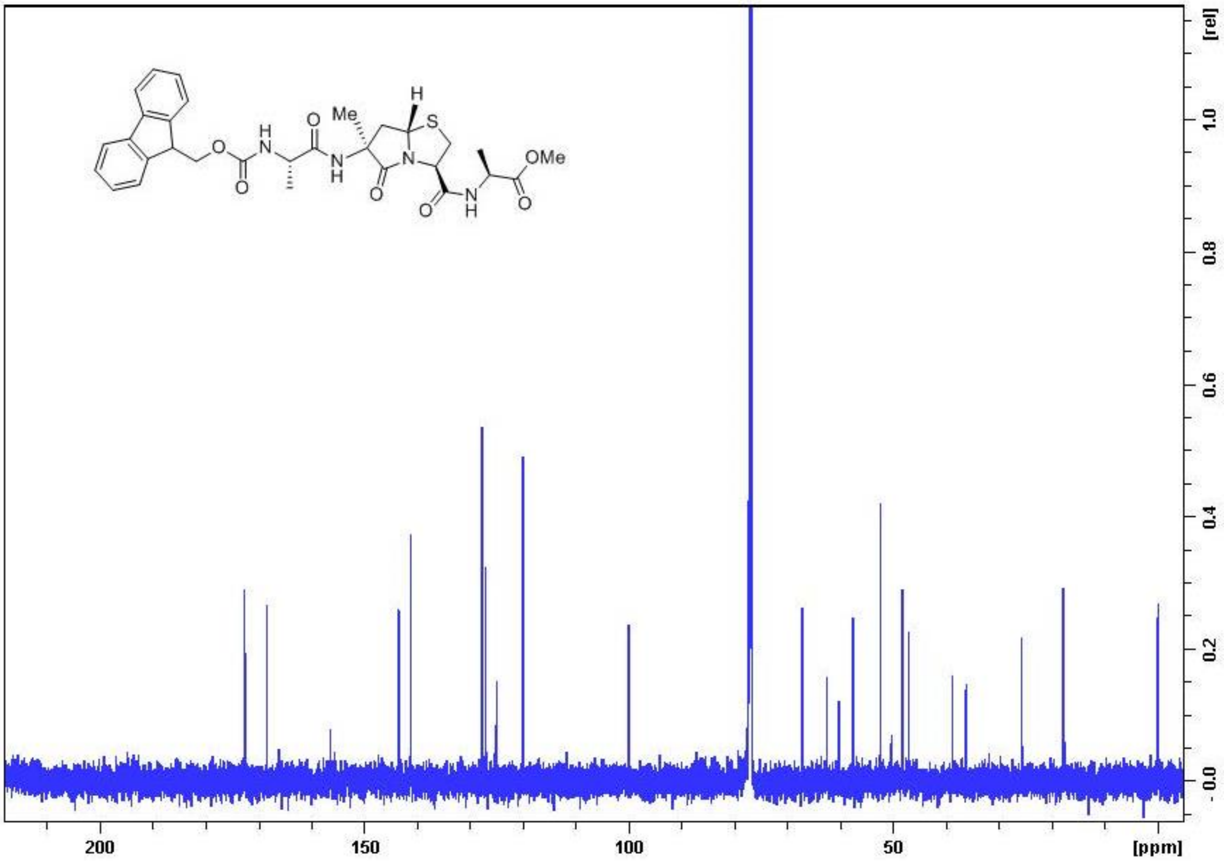

Figure S66. Carbon-13 NMR Spectrum of **α-37b** in $CDCl_3$

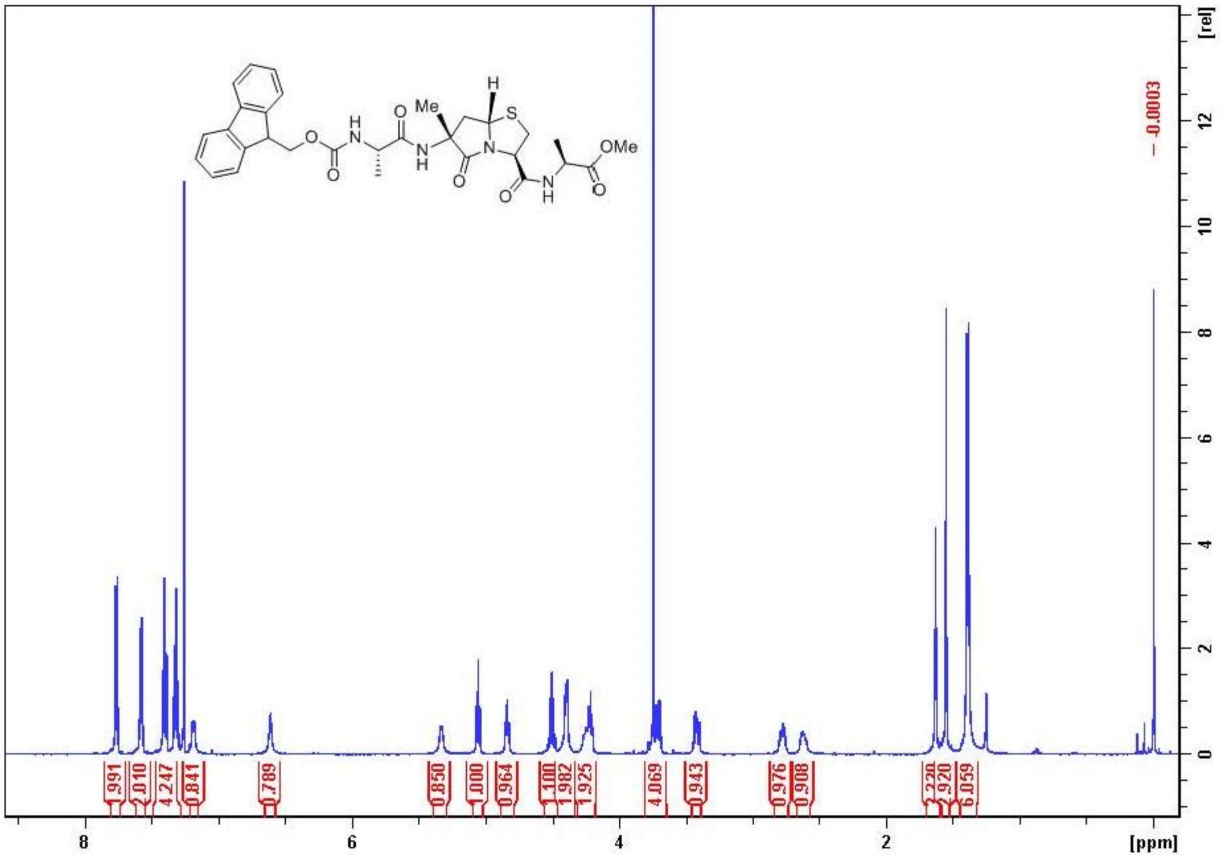

Figure S67. Proton NMR Spectrum of **β-37b** in $CDCl_3$

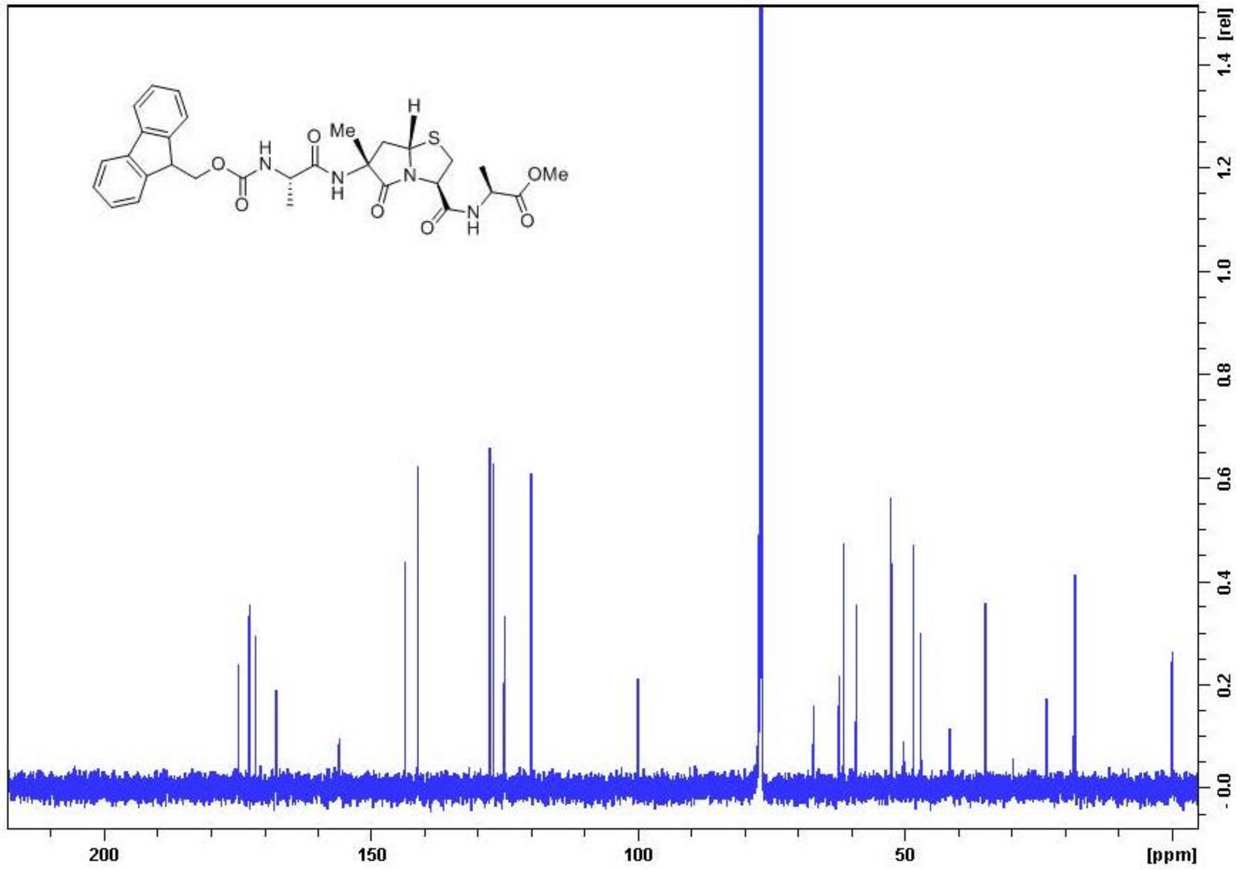

Figure S68. Carbon-13 NMR Spectrum of **β-37b** in $CDCl_3$

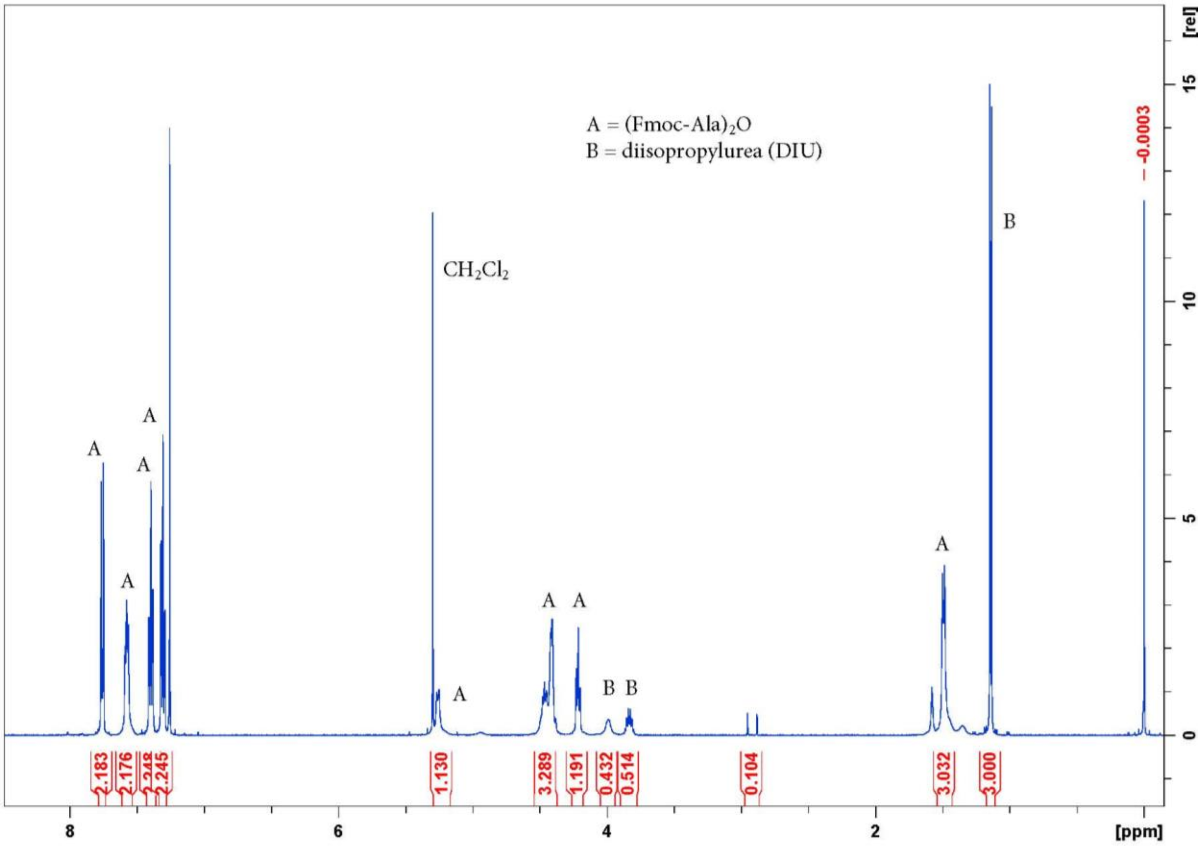

Figure S69. Proton NMR Spectrum of (Fmoc-Ala)$_2$O in $CDCl_3$ with Diisopropylurea (DIU)

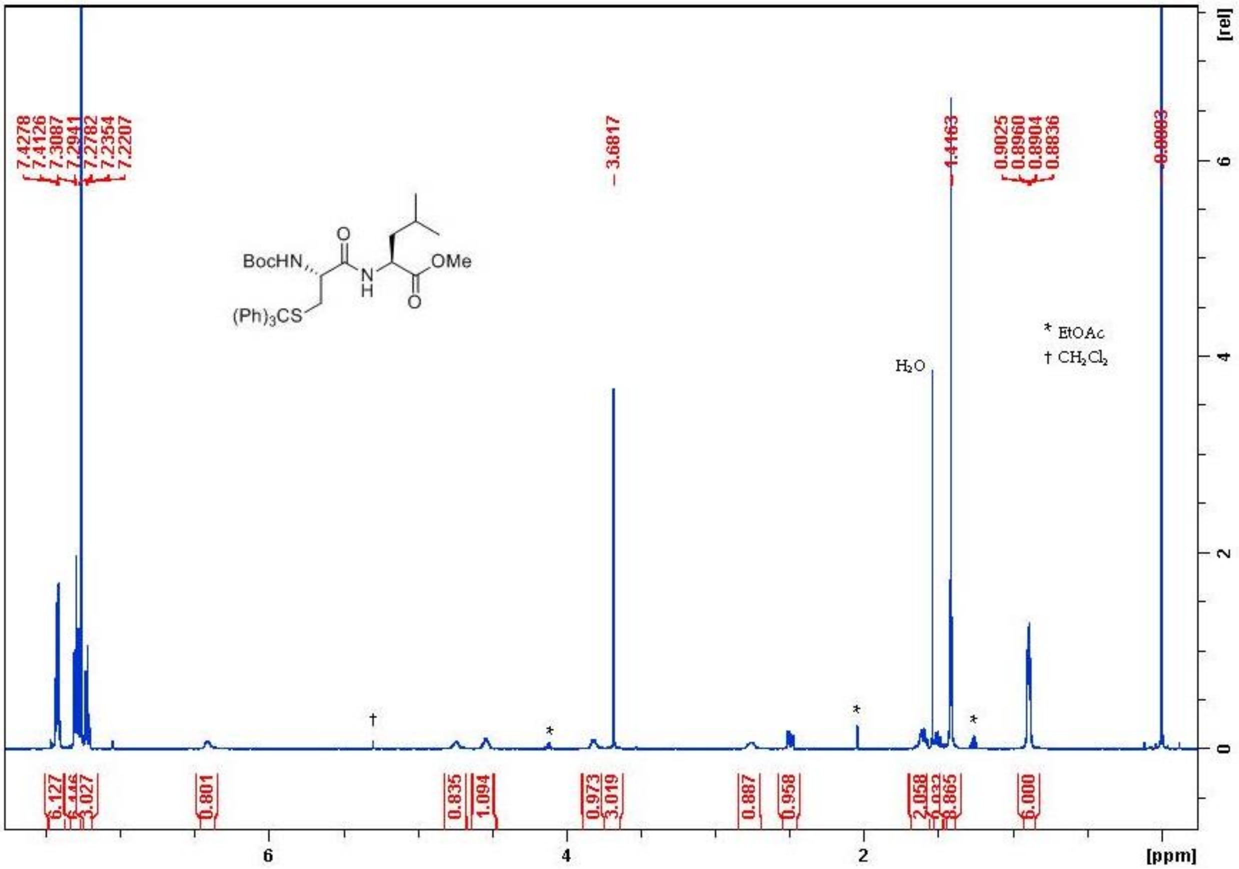

Figure S70. Proton NMR Spectrum of Boc-Cys(Trt)-Leu-OMe in $CDCl_3$

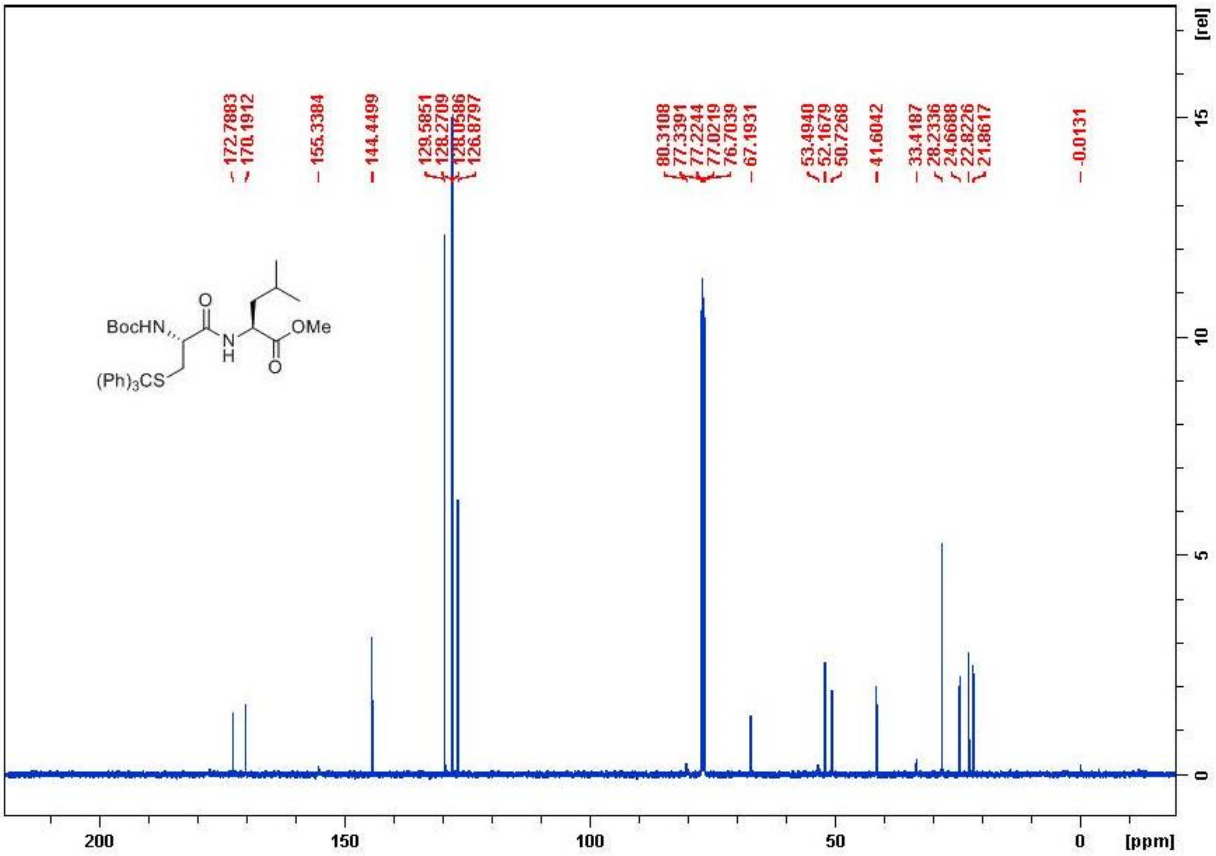

Figure S71. Carbon-13 NMR Spectrum of Boc-Cys(Trt)-Leu-OMe in $CDCl_3$
